# Gut microbiota-mediated secondary bile acid alleviates *Staphylococcus aureus*-induced mastitis through the TGR5-cAMP-PKA-NF-κB/NLRP3 pathways in mice

**DOI:** 10.1038/s41522-023-00374-8

**Published:** 2023-02-08

**Authors:** Caijun Zhao, Keyi Wu, Haoyang Hao, Yihong Zhao, Lijuan Bao, Min Qiu, Yuhong He, Zhaoqi He, Naisheng Zhang, Xiaoyu Hu, Yunhe Fu

**Affiliations:** Department of Clinical Veterinary Medicine, College of Veterinary Medicine, Jilin University, Changchun, Jilin Province 130062 China

**Keywords:** Bacteriology, Pathogens

## Abstract

Although emerging evidence shows that gut microbiota-mediated metabolic changes regulate intestinal pathogen invasions, little is known about whether and how gut microbiota-mediated metabolites affect pathogen infection in the distal organs. In this study, untargeted metabolomics was performed to identify the metabolic changes in a subacute ruminal acidosis (SARA)-associated mastitis model, a mastitis model with increased susceptibility to *Staphylococcus aureus* (*S. aureus*). The results showed that cows with SARA had reduced cholic acid (CA) and deoxycholic acid (DCA) levels compared to healthy cows. Treatment of mice with DCA, but not CA, alleviated *S. aureus*-induced mastitis by improving inflammation and the blood-milk barrier integrity in mice. DCA inhibited the activation of NF-κB and NLRP3 signatures caused by *S. aureus* in the mouse mammary epithelial cells, which was involved in the activation of TGR5. DCA-mediated TGR5 activation inhibited the NF-κB and NLRP3 pathways and mastitis caused by *S. aureus* via activating cAMP and PKA. Moreover, gut-dysbiotic mice had impaired TGR5 activation and aggravated *S. aureus*-induced mastitis, while restoring TGR5 activation by spore-forming bacteria reversed these changes. Furthermore, supplementation of mice with secondary bile acids producer *Clostridium scindens* also activated TGR5 and alleviated *S. aureus*-induced mastitis in mice. These results suggest that impaired secondary bile acid production by gut dysbiosis facilitates the development of *S. aureus*-induced mastitis and highlight a potential strategy for the intervention of distal infection by regulating gut microbial metabolism.

## Introduction

The gut microbiota has been proven to play an important role in maintaining host physiological homeostasis and regulating disease outcomes including infectious disease^1,2^. Mounting evidence reveals that gut microbiota-mediated metabolites are one of the most common mechanisms of host-microbiota interaction and thus participate in the pathogenesis of pathogen invasion^3^. It has been shown that gut dysbiosis-derived metabolites can promote pathogen colonization in multiple manners. For example, gut microbiota-derived 1,2-propanediol regulated the metabolic pathways and increased the virulence expression of *Citrobacter rodentium*, enabling metabolic adaptation and pathogenicity^4^. Microbiota-liberated host sugars, such as sialic acid and fucose, promoted the postantibiotic expansion of *Salmonella typhimurium* and *Clostridium difficile* by serving as preferential energy sources^5^. Tovaglieri et al., also found that gut microbiota-derived metabolites including 4-methyl benzoic acid, 3,4-dimethylbenzoic acid, hexanoic acid and heptanoic acid aggravated enterohemorrhagic *Escherichia coli*-induced intestinal barrier injury by promoting the expression of flagellin^6^. However, it has also been shown that commensal microbiota-derived beneficial components limit pathogen invasion and subsequently improve outcomes of disease. Sun X et al. showed that commensal anaerobic microbiota-produced deoxycholic acid (DCA), a secondary bile acid derived from cholic acid (CA), reduces *Campylobacter jejuni* (*C. jejuni*)-induced colitis, while the depletion of secondary bile acids (BAs)-producing bacteria by clindamycin exacerbates this colitis^7^. In addition, prior gut infection increases the resistance of the microbiota to subsequent infection by increasing taurine, a metabolite derived from BA metabolism, which is then converted to sulfide and inhibits pathogen respiration^8^. Another well-known gut microbiota-derived metabolite that regulates infectious disease is short-chain fatty acids (SCFAs). Commensal *Bacteroides*-produced propionate increases intestinal colonization resistance to *Salmonella* by disrupting intracellular pH homeostasis^9^. Butyrate protects mice against *Clostridium difficile*-induced colitis by improving the intestinal barrier and limiting bacterial translocation by stabilizing the hypoxia-inducible factor 1α^10^. The regulation of gut microbiota structure by depleting commensal *Bacteroides vulgatus* facilitated intestinal *Vibrio cholerae* (*V. cholerae*) pathogenesis in mice by reducing *V. cholerae* growth-inhibitory metabolites including SCFAs but elevating metabolites that enhance *V. cholerae* proliferation including N-acetylglucosamine and gluconolactone^11^. These findings suggest that different gut microbiota-mediated metabolic profiles endow hosts with distinct susceptibilities to pathogens and subsequent outcomes of intestinal diseases. However, little is known about the effects of microbiota-derived metabolites on the pathogenesis of distal pathogen infections.

*Staphylococcus aureus* (*S. aureus*) is a formidable bacterium that can induce severe diseases and even death in humans and animals^12^. Mounting evidence has reported that *S. aureus* could be resistant to most antibiotics, which enables the appearance of new clones, including methicillin-resistant *S. aureus* (MRSA), which serves as a major cause of community-associated infections and lacks safe and effective prevention and control strategies^13^. *S. aureus*-associated mastitis is one of the most common diseases in lactating women and animals, which increases the risk of breast cancer and causes huge economic losses^14,15^. Recently, mounting evidence has indicated that host factors, such as the gut microbiota, participate in the pathogenesis of pathogen-induced mastitis. For example, it has been shown that gut-dysbiotic mice have increased inflammatory responses in the mammary gland and develop exacerbated mastitis symptoms upon bacterial infection^16,17^. Moreover, modification of the gut microbiota and metabolism by fecal microbiota transplantation, probiotics, and direct metabolite supplementation alleviate pathogen-induced mastitis^16,17^. On dairy farms, *S. aureus*-associated mastitis has been associated with gastrointestinal diseases, such as subacute ruminal acidosis (SARA)^18^. A previous study indicated that cows experiencing SARA and challenged intramammarily with lipopolysaccharide (LPS) experienced stronger metabolic disturbances and altered innate immunity^19^. Our previous results also showed that SARA cows had increased susceptibility to *S. aureus*-induced mastitis^20^. These suggest the increased risk of mastitis in cows with SARA, however, the underlying mechanism remains unknown. Previous studies have shown that different dietary patterns affect the microbial composition and metabolic profile in the mammary gland^21,22^, leading to increased susceptibility to mammary diseases^22^. Consumption of the Mediterranean diet increased beneficial microbiota-associated metabolites, including BAs and tryptophan-derived aryl hydrocarbon receptor (AhR) ligands, compared with the Western Diet^21^. These metabolites have been linked to altered susceptibility to bacterial infections and subsequent inflammatory responses^7,17^. However, whether microbiota-associated metabolic changes in the mammary gland of SARA cows are responsible for the increased susceptibility to *S. aureus* is poorly understood along with the underlying mechanism.

In this study, using untargeted metabolomics, we showed that SARA cows had distinct metabolic profiles in their milk compared with heathy cows, especially reduced CA and DCA levels. Consumption of DCA, but not CA, ameliorated *S. aureus*-induced mastitis in mice. The underlying mechanism was involved in the Takeda G protein-coupled receptor 5 (TGR5)-mediated cyclic adenosine monophosphate (cAMP)-protein kinase A (PKA) pathways, which inhibited the activation of proinflammatory NF-κB and NLRP3 in the mammary gland. Gut-dysbiotic mice caused by vancomycin had reduced TGR5 activation in the mammary gland, leading increased susceptibility to *S. aureus*-induced mastitis. Conversely, treatment of mice with spore-forming bacteria (SFB) restored TGR5 activation and alleviated *S. aureus*-induced mastitis. Moreover, commensal *Clostridium scindens* (*C. scindens*) with the capacity of DCA producing also attenuated mastitis caused by *S. aureus* in mice. These results indicate that gut microbiota-mediated DCA contributes to protecting against pathogen-induced mastitis, indicating that regulating gut microbiota and its metabolism can be a potential strategy for the intervention of distal pathogen infection.

## Results

### High concentrate diet-associated SARA reduces BA levels in the mammary glands of cows

**Fig. 1:**
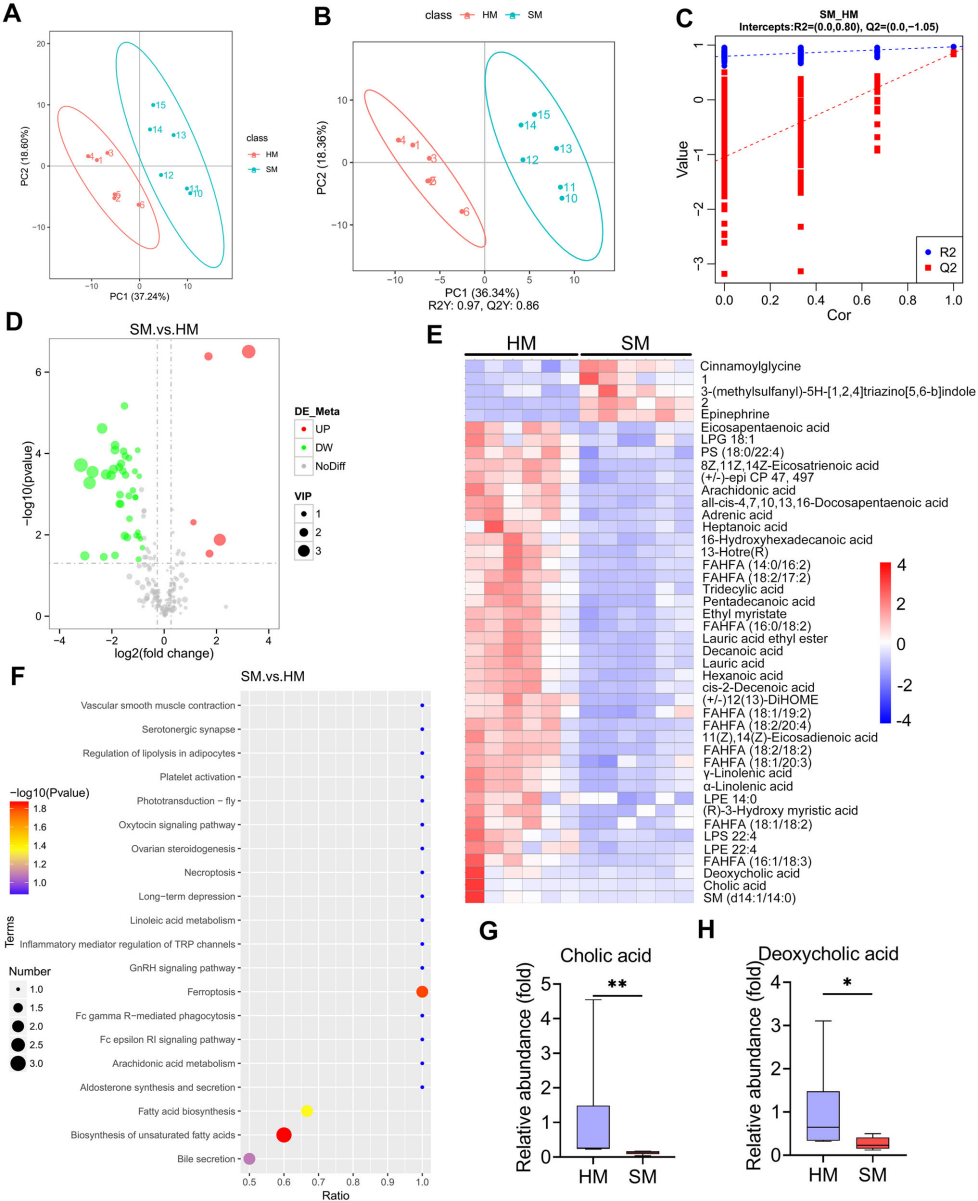
The metabolic profiles of the milk samples from the healthy and SARA cows. **A** PCA score plots for milk samples (*n* = 6). **B** PLS-DA score plots for the milk samples (*n* = 6). **C** Cross-validation plot with a permutation test repeated 200 times. The intercepts of R2 = (0.0, 0.80) and Q2 = (0.0,–1.05), indicate that the PLS-DA model is not overfitting. **D** Volcano plots indicate the results of the pairwise comparisons of metabolites in the healthy and SARA cows. The vertical and horizontal dashed lines indicate the threshold for the twofold abundance difference and the *p* = 0.05 threshold, respectively. Student’s *t*-test was performed for the comparison. The significant metabolites are presented in red (upregulated) or green (downregulated). **E** The hierarchical cluster analysis of the different milk metabolites between healthy and SARA cows. 1. 1-methyl-N-(3-methyl-5-cinnolinyl)-1H-imidazole-4-sulfonamide; 2. N-(1,1-Dioxotetrahydro-1H-1λ6-thiophen-3-yl)-4-methoxybenzamide. **F** The pathway enrichment analysis of significantly elevated metabolites in the SARA samples according to the KEGG pathway. The relative levels of cholic acid (**G**) and deoxycholic acid (**H**) in the healthy and SARA samples were determined. Data are presented as boxplots, with the center line representing the median, the boundary of the whiskers representing the minimum and maximum values of the dataset, and the boundary of the box representing the 25th and 75th percentile of the dataset (G and H). Mann-Whitney *U* test was performed (**G**, **H**). *p < 0.05, **p < 0.01. PLS-DA, partial least squares discrimination analysis; VIP, variable importance in the projection.

Our previous results showed that SARA cows had increased susceptibility to *S. aureus*-induced mastitis^20^. To investigate the underlying metabolic changes, untargeted metabolomics was performed on milk samples from healthy and SARA cows. To acquire stable and accurate metabolome results, data quality control (QC) was performed through the Pearson correlation analysis. The Pearson correlation of the ruminal QC samples was high, suggesting reliable data quality (Supplementary Fig. 1A). Moreover, the QC samples clustered tightly together in the principal component analysis (PCA) score plots (Supplementary Fig. 1B, C), which further confirmed quality of the data. A total of 213 annotated metabolites were identified in all the milk samples by using the HMDB annotation, Kyoto Encyclopedia of Genes and Genomes (KEGG), and LIPID MAPS (Supplementary Fig. 2A–C, Supplementary Table 1). PCA showed that the milk samples from the cows with SARA (SM) were significantly separated from the healthy cows (HM) (PC1 was 37.24% and PC2 was 18.60%, Fig. 1A). Moreover, distinct clusters from the SM compared with the HM were confirmed by the partial least squares discrimination analysis (PLS-DA) score plots (*p* < 0.001, Fig. 1B). The stability and reliability of the PLS-DA were confirmed by 7-fold cross-validation (R2Y:0.97, Q2Y:0.86, Fig. 1B). Furthermore, a model containing metabolic information by fitting the PLS-DA through 200 times random permutation testing was performed to confirm the ability of the PLS-DA to correctly classify new samples. The results demonstrated that the model was reliable and not overfitting, as shown by the intercepts of the goodness-of-fit (R2) being greater than the goodness-of-prediction (Q2) and the intercept of Q2 less than zero (Fig. 1C).

We identified 44 significantly differential metabolites in the SM compared with the HM (5 upregulated and 39 downregulated, Supplementary Table 1, 2, and 3) by considering the variable importance in the projection (VIP), fold change (FC), and *P*-value. The global distribution of different metabolites was shown by volcano plots, which indicated distinct metabolite levels between the SM and HM (Fig. 1D). Moreover, the metabolite levels in the SM were significantly different from those in the HM through differential metabolite cluster analysis by using a heatmap (Fig. 1E). KEGG pathway enrichment analysis indicated that the pathways enriched in the SM were mainly bile secretion, biosynthesis of unsaturated fatty acids, fatty acid biosynthesis and ferroptosis (Fig. 1F). Considering the role of gut microbiota in the pathogenesis of pathogen-induced mastitis^16,17^ and microbiota-mediated BA metabolism in regulating gut pathogen invasion^7,8^, we further focused on bile metabolism and showed that the secondary bile acid DCA and primary bile acid CA were significantly depleted in the SM samples compared with those in the HM group (Fig. 1G, H). Notably, our previously published rumen microbiome analysis of these SARA cows indicates that they have fewer *Clostridia* which might include those capable of making DCA from CA but that genus-level data is insufficient to know for sure^20^. Collectively, these results indicated that SARA cows had altered metabolic profiles in their milk, especially reduced BAs, which may thus promote *S. aureus*-induced mastitis.

### The secondary bile acid DCA, but not the primary bile acid CA, alleviates *S. aureus*-induced mastitis in mice

**Fig. 2:**
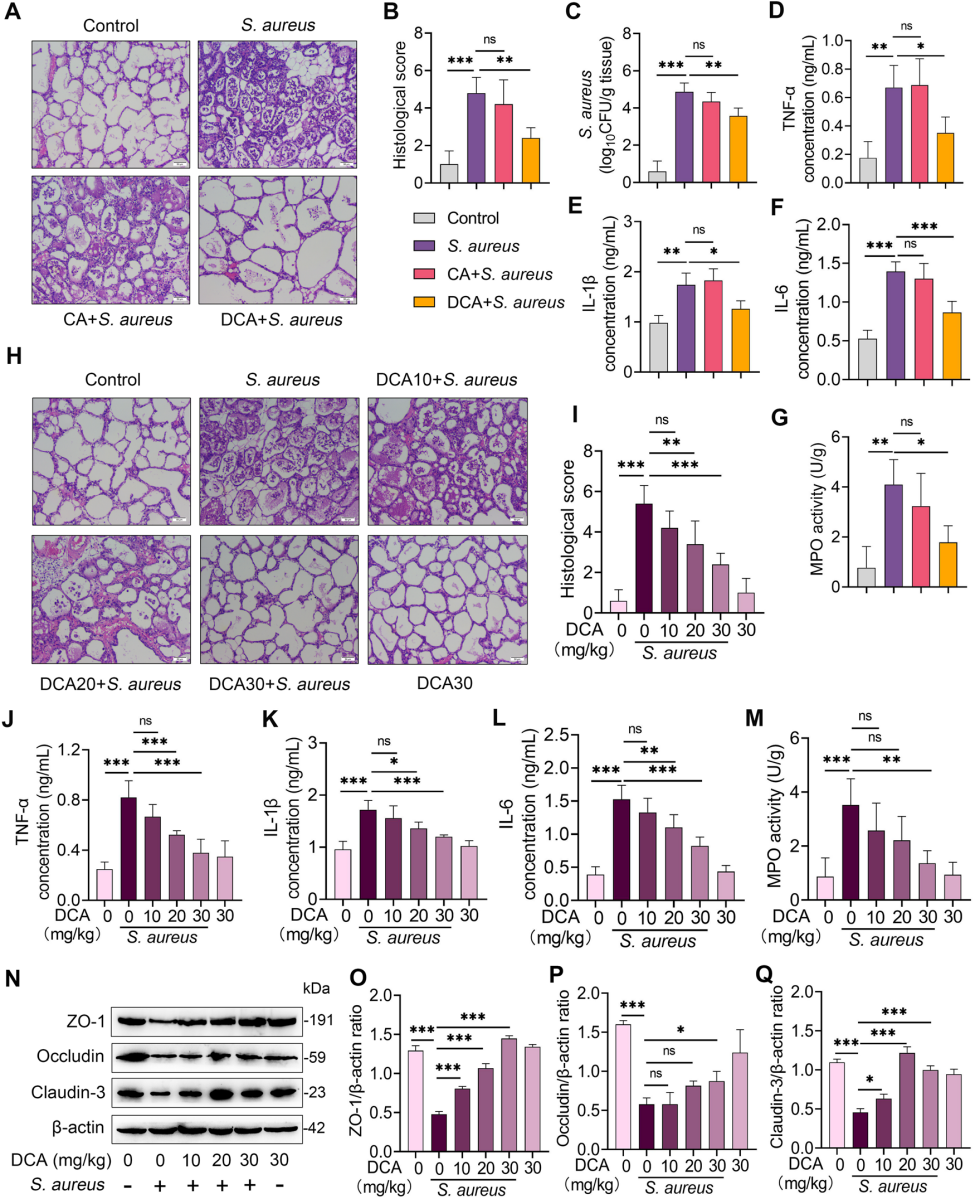
DCA but not CA alleviates *S. aureus*-induced mastitis in mice. Mice were pretreated with 30 mg/kg of CA or DCA for 2 h intraperitoneally followed by *S. aureus*-induced mastitis. Twenty-four hours after *S. aureus* infection, the mammary tissues were collected for determination. **A** Representative H&E-stained sections of the mammary gland from the indicated groups (scale bar, 50 μm). **B** Histological score for the mammary gland based on H&E-stained sections (*n* = 6). **C** Mammary *S. aureus* load (*n* = 6). Inflammatory parameters of the mammary gland from different groups, including TNF-α (**D**), IL-1β (**E**), and IL-6 (**F**) concentrations and MPO activity (**G**), were performed (*n* = 6). **H–Q** Mice were pretreated with different dose of DCA (10, 20, and 30 mg/kg) for 2 h intraperitoneally and then stimulated with *S. aureus* for next 24 h. **H** Representative mammary H&E-stained sections from the indicated mice (scale bar, 50 μm). **I** Histological score of the mammary gland from different treatment groups (*n* = 6). The inflammatory parameters of the mammary gland from different treatment groups, TNF-α (**J**), IL-1β (**K**), and IL-6 (**L**) concentrations and MPO activity (**M**), were measured (*n* = 6). **N** Represented images of ZO-1, Occludin, and Claudin-3 in the mammary glands from the indicated mice. The intensities of ZO-1, Occludin, and Claudin-3 were determined (**O–Q**). Data are expressed as the means ± SD (**B-G, I–M**, and **O–Q**) and one-way ANOVA was performed, followed by Tukey’s test (**B–G, I-M**, and **O–Q**). *p < 0.05, **p < 0.01, ***p < 0.001 indicate significance. ns no significance.

We next investigated the role of BAs in mastitis using a *S. aureus*-induced mouse mastitis model. The results showed that *S. aureus* treatment induced significant mastitis traits by increasing mammary injury and inflammatory infiltration and responses compared with the control mice (Fig. 2A–G). Pretreatment with DCA intraperitoneally, but not CA, alleviated *S. aureus*-induced mastitis, as evidenced by DCA improving mammary injury (Fig. 2A, B), mammary *S. aureus* burden (Fig. 2C), proinflammatory markers including TNF-α, IL-1β and IL-6 and MPO activity (Fig. 2D–G) compared with that of the *S. aureus* group. To confirm the protective effects of DCA on *S. aureus*-induced mastitis, mice were pretreated with different doses of DCA and the results found that DCA ameliorated *S. aureus*-induced mammary damage (Fig. 2H, I) and inflammatory parameters (Fig. 2J–M) in a dose-dependent manner. Increased mammary immune cell infiltration and inflammatory responses were associated with impaired blood-milk barrier integrity^16,17,20^. Indeed, DCA pretreatment improved the mammary barrier disruption resulting from *S. aureus*, as evidenced by the restoration of the tight-junction (TJ) proteins ZO-1, Occludin and Claudin-3 compared with those in the *S. aureus*-treated group (Fig. 2N–Q). These results suggest that the secondary bile acid DCA, but not the primary bile acid CA, improves *S. aureus*-induced mastitis in mice.

### DCA attenuates *S. aureus*-induced mastitis through the TGR5-cAMP-PKA-NF-κB/NLRP3 pathways

**Fig. 3:**
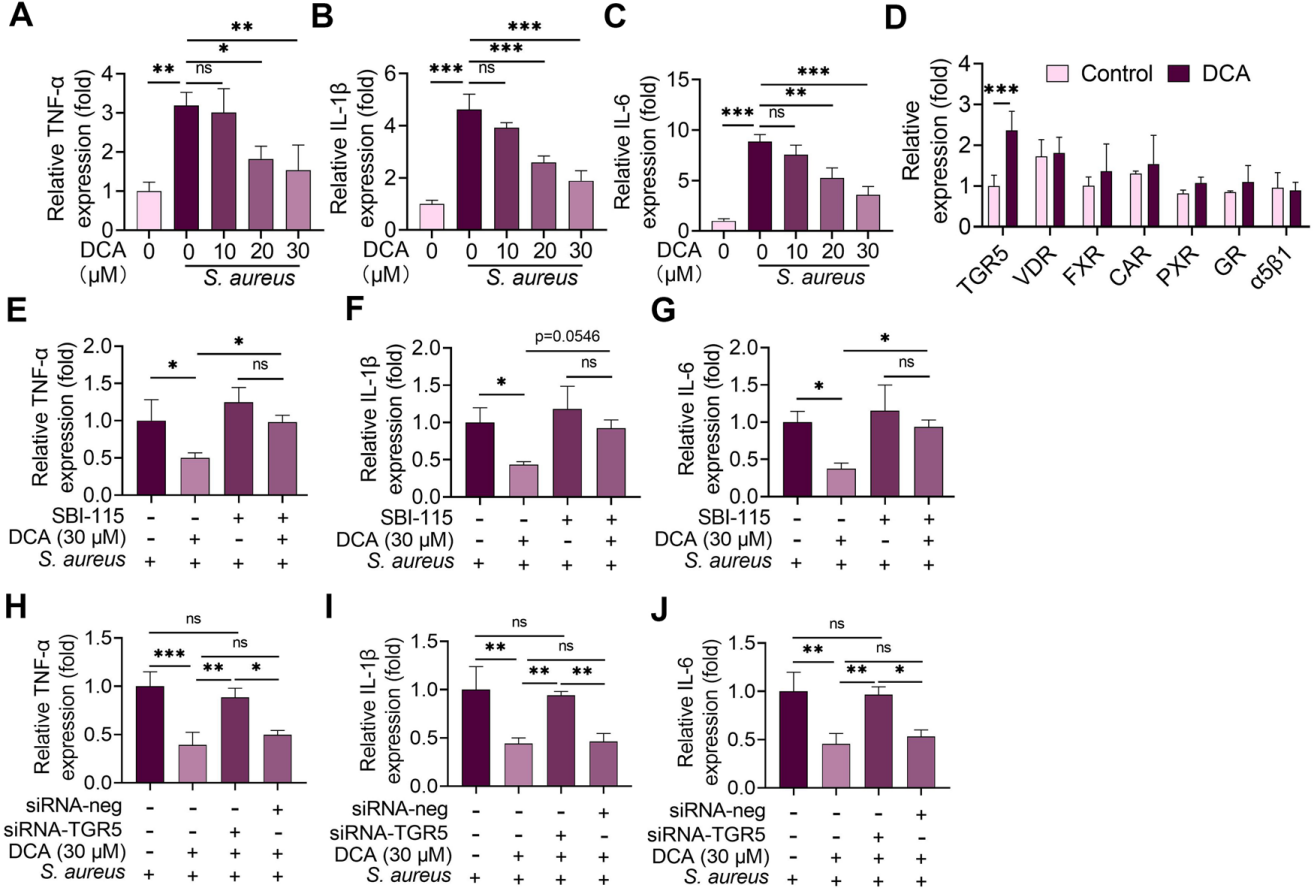
DCA alleviates *S. aureus*-induced inflammation in MMECs by activating TGR5. Cells were pretreated with DCA (10, 20 and 30 μM) for 2 h followed by *S. aureus* treatment for next 24 h, and the relative mRNA levels of proinflammatory TNF-α (**A**), IL-1β (**B**), and IL-6 (**C**) from the indicated group were detected by qPCR. **D** Cells were treated with DCA (30 μM) for 24 h and the relative mRNA levels of TGR5, VDR, FXR, CAR, PXR, GR and α5β1 were determined using qPCR. Cells were pre-treated with SBI-115 for 2 h and then treated with DCA (30 μM) for an additional 2 h followed by *S. aureus* treatment for the next 24 h. Relative mRNA levels of proinflammatory TNF-α (**E**), IL-1β (**F**), and IL-6 (**G**) from the different treatment groups were detected by qPCR. **H–J** Cells were pretreated with siRNA-TGR5 or siRNA-neg for 48 h followed by DCA (30 μM) and *S. aureus* treatment as mentioned above, and the relative mRNA levels were detected by qPCR. Data are expressed as the means ± SD (**A–J**) and one-way ANOVA followed by Tukey’s test (**A–C** and **E–J**) and Student’s *t*-test was performed (**D**). *p < 0.05, **p < 0.01, ***p < 0.001 indicate significance. ns no significance.

It is well known that BAs have different antimicrobial abilities and the biofilm of *S. aureus* contributes to its infection^7,23^. We, therefore, investigated whether DCA and CA regulated the growth and biofilm formation of *S. aureus*. The results showed that DCA (636.82 μM reduced the OD600 by almost 50%) had a better capacity to limit *S. aureus* growth in vitro than CA (1835.67 μM had few inhibitory effects) (Supplementary Fig. 3A–B). In addition, no significant influences on the biofilm formation of *S. aureus* were observed in less than 60 μM of DCA and CA treatment groups (Supplementary Fig. 3C, D). Consistent with the results found in other cell types^24^, a dose of DCA that did not inhibit the growth of *S. aureus* reduced *S. aureus*-induced TNF-α, IL-1β and IL-6 mRNA in the mouse mammary epithelial cells (MMECs) (Fig. 3A–C), suggesting that DCA-alleviated *S. aureus*-induced inflammation is not entirely dependent on the inhibition of *S. aureus* growth. Secondary BAs were reported by multiple receptors^25–27^, including the vitamin D receptor (VDR), Takeda G protein-coupled receptor 5 (TGR5), α5β1, glucocorticoid receptor (GR), constitutive androstane receptor (CAR), farnesoid X receptor (FXR), and pregnane X receptor (PXR). We next found that only TGR5 was markedly increased in the DCA-treated MMECs compared with the control group (Fig. 3D). To confirm the role of TGR5 activation in the effects of DCA, we blocked TGR5 activation with the specific inhibitor SBI-115^28^ or siRNA-TGR5. Both SBI-115 and siRNA-TGR5 reversed the protective effects of DCA on *S. aureus-*induced proinflammatory cytokine production in the MMECs (Fig. 3E–J). Although CA has also been reported to activate TGR5, we found that 30 μM of the CA treatment had no significant influence on *S. aureus-*induced proinflammatory cytokine production in the MMECs (Supplementary Fig. 4A–C). These results indicate that TGR5 activation is responsible for the protective effects of DCA on *S. aureus-*induced mastitis.

**Fig. 4:**
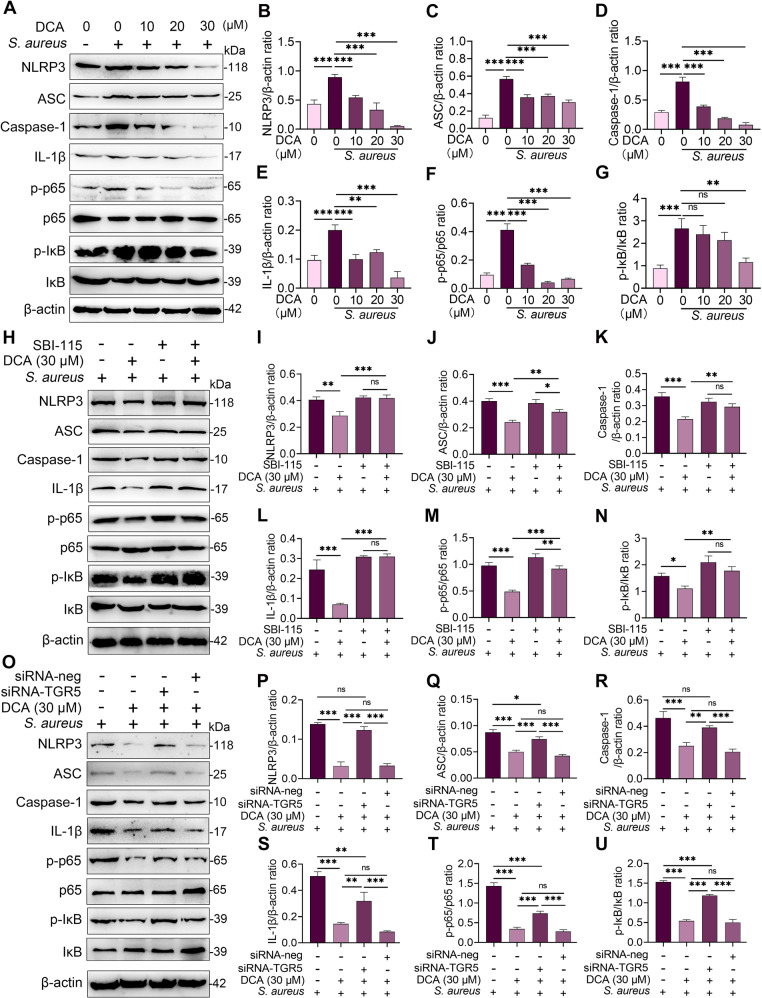
DCA inhibits *S. aureus*-induced NF-κB and NLRP3 activation by activating TGR5 in MMECs. **A–G** Cells were pretreated with DCA (10, 20, and 30 μM) for 2 h followed by *S. aureus* treatment for the next 24 h and the protein levels of the NF-κB and NLRP3 pathways from the indicated groups were determined by western blotting (**A**). The relative intensities of p-p65, p-IκB, NLRP3, ASC, and IL-1β were determined (**B–G**). **H–N** Cells were pretreated with SBI-115 for 2 h and then treated with DCA (30 μM) for an additional 2 h followed by *S. aureus* treatment for the next 24 h. The protein levels of the NF-κB and NLRP3 pathways were determined by western blotting (**H**), and the relative intensities of p-p65, p-IκB, NLRP3, ASC, and IL-1β were determined (**I–N**). **O–U**. Cells were pretreated with siRNA-TGR5 or siRNA-neg for 48 h followed by DCA (30 μM) and *S. aureus* treatment as mentioned above, and the protein levels of the NF-κB and NLRP3 pathways were determined by western blotting. Data are expressed as the means ± SD (**B–G, I–N**, and **P–U**) and one-way ANOVA was performed, followed by Tukey’s test (**B–G, I–N**, and **P–U**). *p < 0.05, **p < 0.01, ***p < 0.001 indicate significance. ns, no significance.

**Fig. 5:**
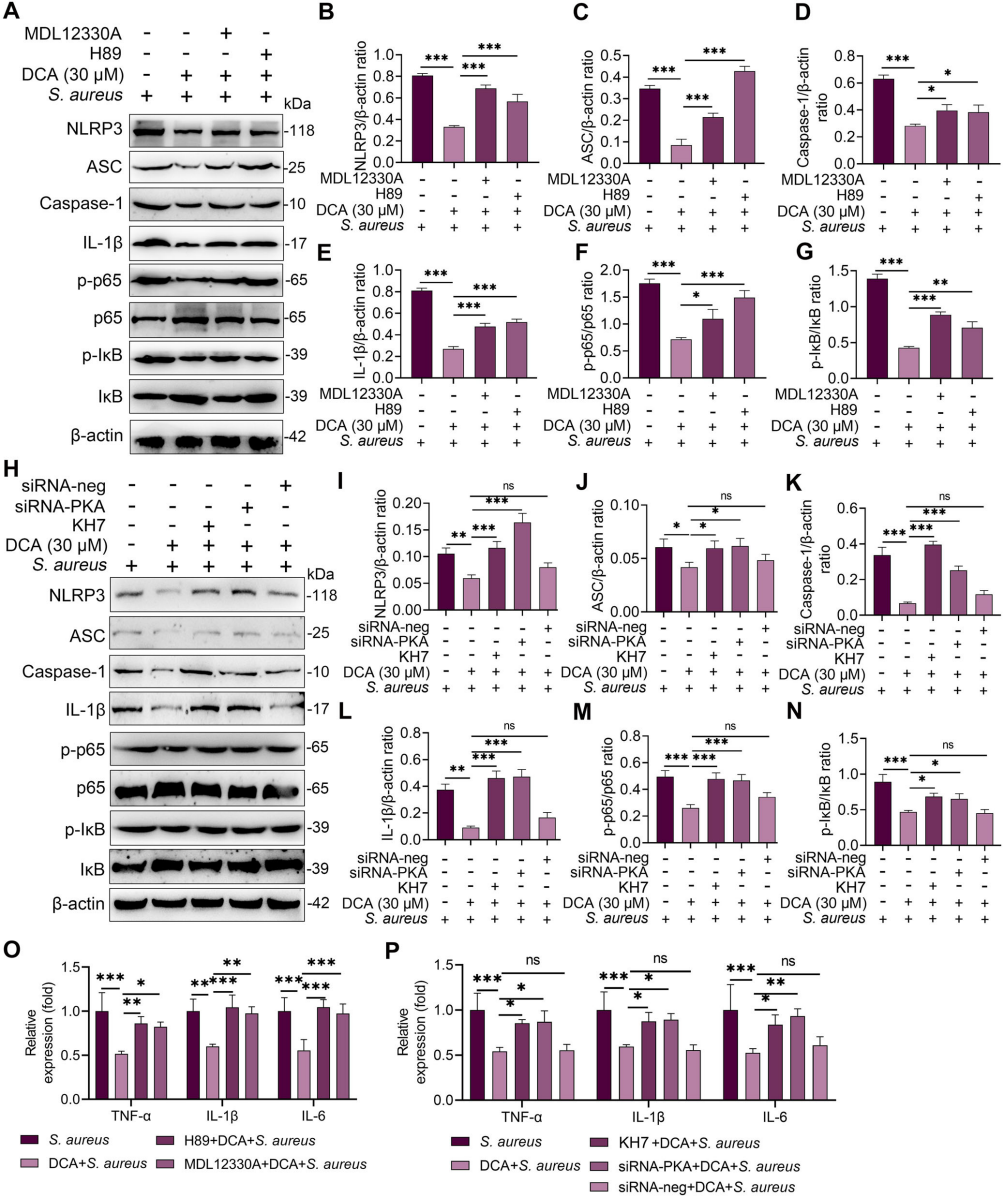
DCA alleviates *S. aureus*-induced inflammation in MMECs through the cAMP-PKA- NF-Κb/NLRP3 pathways. **A–G** and **O**. Cells were pretreated with H89 (30 μM) and MDL12330A (10 μM) for 2 h and then treated with DCA (30 μM) for an additional 2 h followed by *S. aureus* treatment for the next 24 h. The protein levels of NF-κB and NLRP3 pathways from the indicated groups were determined by western blotting (**A**). The relative intensities of p-p65, p-IκB, NLRP3, ASC, and IL-1β were determined (**B–G**). The relative mRNA levels of proinflammatory gene from the indicated group were detected by qPCR (**O**). **H–N** and **P**. Cells were pretreated with siRNA for 48 h and then treated with DCA (30 μM) and KH7 (10 μM) for an additional 2 h followed by *S. aureus* treatment for the next 24 h. The protein levels of NF-κB and NLRP3 pathways from the indicated groups were determined by western blotting (**H**) and The relative intensities of p-p65, p-IκB, NLRP3, ASC and IL-1β were determined (**I–N**). The relative mRNA levels of proinflammatory gene from the indicated group were detected by qPCR (**P**). Data are expressed as the means ± SD (**B–G, I–N** and **O**, **P**) and one-way ANOVA was performed, followed by Tukey’s test (**B–G, I–N**, and **O**, **P**). *p < 0.05, **p < 0.01, ***p < 0.001 indicate significance.

Proinflammatory cytokine secretion was attributed to the activation of proinflammatory pathways, such as NF-κB and NLRP3^29^. As expected, DCA treatment inhibited the activation of the NF-κB and NLRP3 pathways in the *S. aureus*-treated MMECs in a dose-dependent manner (Fig. 4A–G). We further investigated whether these effects were mediated by TGR5 activation. The results showed that the SBI-115 and siRNA-TGR5 treatments reversed the reduction in p-p65, p-IκB, NLRP3, and IL-1β resulting from DCA treatment in the *S. aureus*-treated MMECs (Fig. 4H–U), leading to TGR5 activation inhibiting *S. aureus-*induced activation of the NF-κB and NLRP3 pathways. Previous studies have shown that TGR5 binding with ligands contributes to cAMP production, leading to the activation of PKA^24,26^. We, therefore, investigated whether the cAMP-PKA pathway is involved in TGR5-mediated NF-κB and NLRP3 inhibition by using the adenylate cyclase inhibitor MDL12330A and PKA inhibitor H89^30,31^. Pretreatment with both MDL12330A and H89 reversed the protective effect of TGR5 activation on NF-κB and NLRP3 activation (Fig. 5A–G). To confirm these results, another cAMP inhibitor KH7, and siRNA-PKA were also performed. Consistently, the KH7 and siRNA-PKA treatments reversed the decrease in NF-κB and NLRP3 activation induced by DCA in *S. aureus*-treated MMECs (Fig. 5H–N). Moreover, the inhibition of cAMP and PKA by inhibitor and siRNA reversed the decrease in TNF-α, IL-1β, and IL-6 expression caused by DCA in *S. aureus*-treated MMECs (Fig. 5O, P). Collectively, these results indicated that DCA alleviated *S. aureus*-induced mastitis through the TGR5-cAMP-PKA-NF-κB/NLRP3 axis.

### Gut microbiota-produced DCA mediates TGR5 activation and the development of *S. aureus*-induced mastitis in mice

**Fig. 6:**
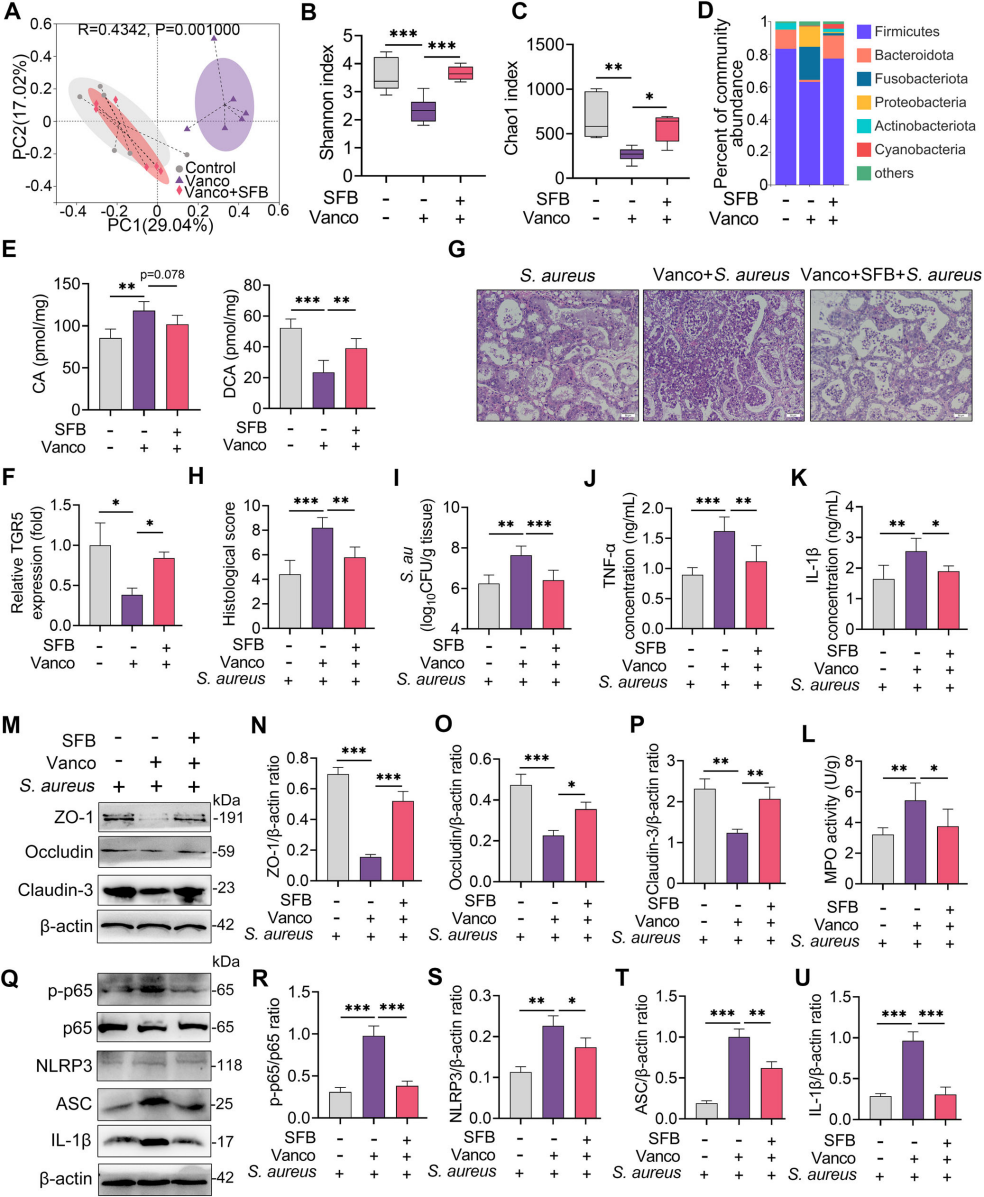
Gut dysbiosis impairs TGR5 activation and aggravates *S. aureus*-induced mastitis in mice. Mice were treated with vancomycin and supplemented with SFB, followed by S. aureus-induced mastitis. **A** Principal coordinate analysis score plots indicate the separation of intestinal microbiota structure (*R* = 0.4342, *P* = 0.001) in the vancomycin-treated mice from that of control or SFB-transplanted mice based on Bray-Curtis distance (*n* = 6). **B-C** The Shannon (**B**) and Chao1 (**C**) indices showed that vancomycin reduced the gut microbial diversity and richness but was restored by SFB transplantation (*n* = 6). **D** The gut microbial composition at the phylum level from different treatment groups. **E** Mammary DCA and CA levels from the indicated groups were detected (*n* = 6). **F** The relative expression of TGR5 mRNA from different treatment groups (*n* = 6). **G** Representative images of H&E-stained sections from different groups (scale bar, 50 μm). **H** The histological score of the mammary gland from different groups (*n* = 6). **I** Mammary *S. aureus* load. The proinflammatory markers of TNF-α (**J**), IL-1β (**K**), and MPO activity (**L**) were measured (*n* = 6). **M** Representative images of ZO-1, Occludin and Claudin-3 of the mammary gland from the indicated mice. The intensities of ZO-1, Occludin and Claudin-3 were determined (**N–P**). **Q** The protein levels of the NF-κB and NLRP3 pathways in the mammary gland were detected using western blotting. The relative intensities of p-p65, NLRP3, ASC, and IL-1β in the mammary gland were detected (**R–U**). Data are expressed as boxplots, with the center line representing the median, the boundary of the whiskers representing the minimum and maximum values of the dataset, and the boundary of the box representing the 25th and 75th percentile of the dataset (**B**, **C**) or means ± SD (**E**, **F**, **H-L, N–P**, and **R–U**) and one-way ANOVA was performed, followed by Tukey’s test (**E**, **F**, **H–L, N–P** and **R–U**). *p < 0.05, **p < 0.01, ***p < 0.001 indicate significance.

Gut microbiota plays an important role in the translation of primary into secondary BAs^32,33^, such as CA converting into DCA by gut microbiota-mediated enzymes. Together with the different effects of DCA and CA on *S. aureus*-induced mastitis, we thus investigated the role of the gut microbiota in the pathogenesis of mastitis caused by *S. aureus*. Vancomycin-sensitive anaerobic microbes are known as the predominant taxa for secondary BA production^34,35^. We then modified the gut microbiota by depleting gut anaerobic microbes using vancomycin and compensating with anaerobic SFB. Principal coordinate analysis based on the Bray-Curtis distance showed that the gut microbiota structure of the vancomycin treatment group was markedly separated from that of the control or SFB treatment groups (*R* = 0.4342, *P* = 0.001) (Fig. 6A). Venn diagram analysis found that the vancomycin-treated mice had reduced species in the gut microbiota compared with the control group but reversed after the SFB compensation (Supplementary Fig. 5A). Alpha diversity analysis, including Shannon, Chao1, ACE and Simpson indices, showed that the vancomycin-treated group had reduced richness and diversity of the gut microbiota compared with that of the control and SFB compensation groups (Fig. 6B, C and Supplementary Fig. 5B, C). At the phylum level, the vancomycin treatment reduced the abundance of the obligate anaerobic *Firmicutes* and *Bacteroidota*, but enriched the abundance of *Fusobacteriota* and *Proteobacteria*, while these changes were reversed after the SFB transplantation (Fig. 6D). At the genus level, the vancomycin-treated mice also showed distinct microbial compositions from those of the control or SFB compensation groups (Supplementary Fig. 5D). Linear discriminant analysis effect size (LEfSe) showed that *Clostridium* and *Fusobacterium* were significantly changed among different groups (Supplementary Fig. 5E). We next showed that the vancomycin treatment reduced DCA but increased CA levels in the mammary gland compared with the control and SFB treatment groups (Fig. 6E). Consistently, the vancomycin treatment reduced the expression of TGR5 in the mammary gland compared with the control group, but was restored after the SFB compensation (Fig. 6F). Moreover, the vancomycin-treated mice developed more severe mastitis than the control and SFB compensation mice upon *S. aureus* infection, as shown by aggravated mammary damage, bacterial burden and proinflammatory parameters (Fig. 6G–L). However, the SFB transplantation from the control mice alleviated *S. aureus*-induced mastitis compared with that of the vancomycin-treated mice (Fig. 6G–L). Likewise, the vancomycin-treated mice had reduced TJ expressions and increased NF-κB and NLRP3 activation compared with the control group, while the SFB treatment restored the barrier function and limited NF-κB and NLRP3 activation caused by *S. aureus* (Fig. 6M–U). To confirm the protective role of gut microbiota-metabolized DCA in *S. aureus*-induced mastitis, CA and DCA were performed orally in conventional and gut-dysbiotic mice. Interestingly, oral gavage with CA alleviated *S. aureus*-induced mastitis in the conventional mice but reversed after the depletion of commensal microbiota by an antibiotic cocktail, accompanied by changed DCA levels in the mammary gland (Supplementary Fig. 6A–F). Moreover, treatment with DCA in gut-dysbiotic mice still alleviated *S. aureus*-induced mastitis (Supplementary Fig. 6A–F). These results indicate that gut microbiota-produced DCA mediates the activation of TGR5 and the development of *S. aureus*-induced mastitis in mice.

### *C. scindens* with the capacity of secondary BA production alleviates *S. aureus-*induced mastitis in mice

**Fig. 7:**
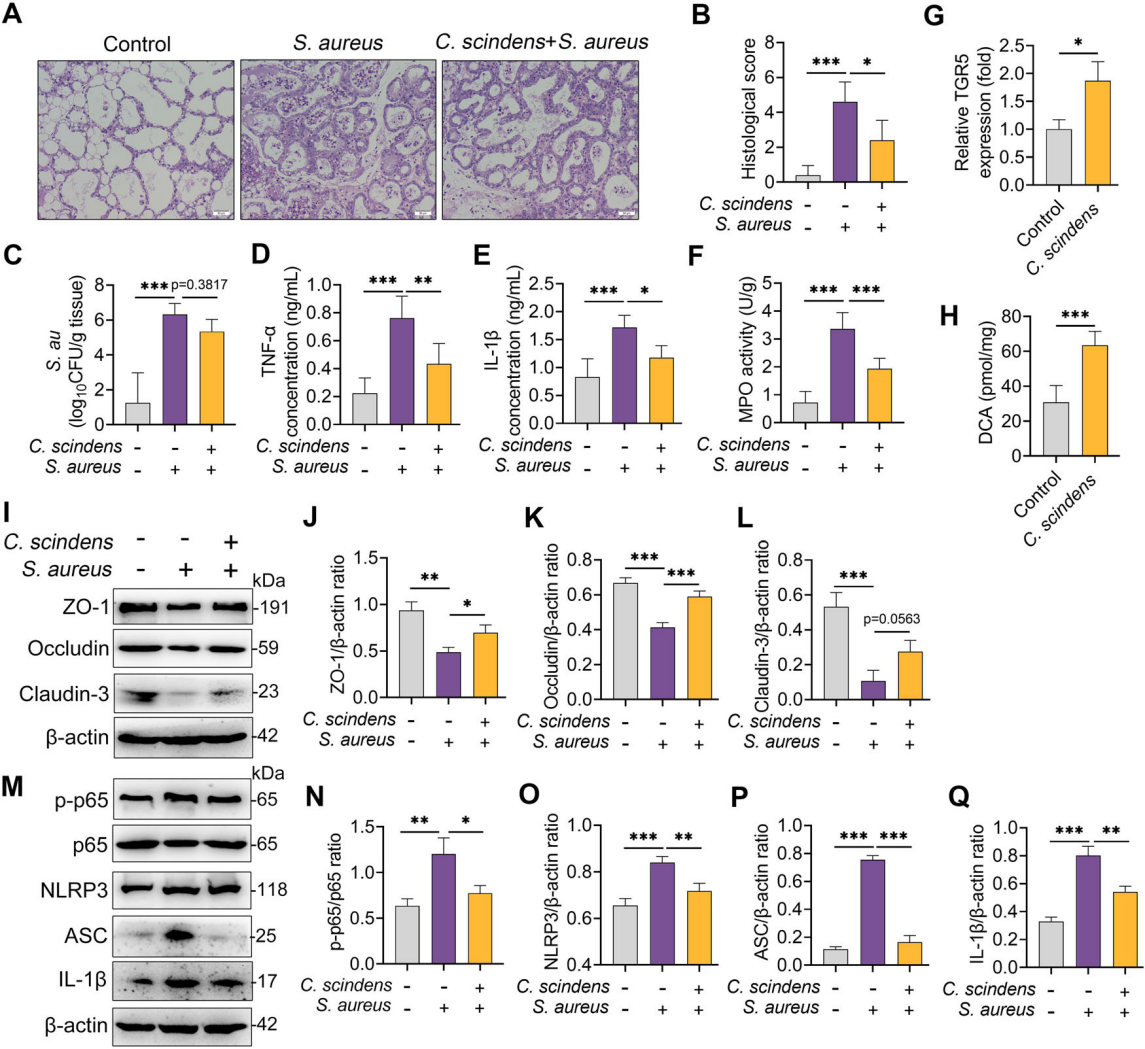
*Clostridium scindens* with DCA-producing capacity alleviates *S. aureus*-induced mastitis in mice. **A** Representative images of the H&E-stained sections from different groups (scale bar, 50 μm). **B** The histological score of the mammary gland from different groups (*n* = 6). **C** Mammary *S. aureus* load (*n* = 6). The proinflammatory markers of TNF-α (**D**), IL-1β (**E**), and MPO activity (**F**) were measured (*n* = 6). **G** Relative expression of TGR5 mRNA from different treated groups (*n* = 6). **H** Mammary DCA levels from the indicated mice (*n* = 6). **I** Representative images of ZO-1, Occludin, and Claudin-3 of the mammary gland from the indicated mice. Intensities of ZO-1, Occludin, and Claudin-3 were determined (**J–L**). **M** The protein levels of NF-κB and NLRP3 pathways of the mammary glands were detected using western blotting. The relative intensities of p-p65, NLRP3, ASC, and IL-1β of the mammary glands were detected (**N–Q**). Data are expressed as the means ± SD (**B–H**, **J–L**, and **N–Q**) and one-way ANOVA was performed, followed by Tukey’s test (**B–H**, **J–L**, and **N–Q**). *p < 0.05, **p < 0.01, ***p < 0.001 indicate significance.

We next investigated the effects of *C. scindens*, a commensal microbe known to convert primary BAs into secondary BAs^26,36^, on *S. aureus*-induced mastitis in mice. As expected, colonization with *C. scindens* alleviated *S. aureus*-induced mammary damage (Fig. 7A, B), the *S. aureus* burden (Fig. 7C), and proinflammatory marker expressions (Fig. 7D–F). Notably, the *C. scindens*-colonized mice had increased TGR5 gene expression and DCA levels in their mammary glands compared with the control mice (Fig. 7G, H). Moreover, *C. scindens* supplementation alleviated *S. aureus*-induced blood-milk barrier damage, as evidenced by restored TJ proteins including ZO-1, Occludin, and Claudin-3 compared with the *S. aureus* treatment group (Fig. 7I–L). In addition, treating with *C. scindens* ameliorated *S. aureus*-induced NF-κB and NLRP3 activation in the mammary glands (Fig. 7M–Q). These results suggest that secondary BA producer improved *S. aureus*-induced mastitis through the activation of TGR5 in mice.

## Discussion

MRSA-associated infections are of significant concern for humans and animals^13^. Mounting evidence suggests that an alternated host’s state endows different susceptibilities to pathogens including MRSA^6,16,17^, which may be attributed to distinct metabolic profiles caused by the host and gut microbiota^3,4,11^. However, whether and how gut microbiota-derived metabolic changes contribute to the development of MRSA-associated diseases in distal organs remains unknown. In this study, we identified significantly different metabolic characteristics in the mammary glands of SARA cows, which may be involved in the changed susceptibility to *S. aureus*-induced mastitis^20^. Among the metabolites associated with gut microbiota is the BA metabolism characterized by reduced CA and DCA in the SARA cows. Supplementation of the mice with DCA, but not CA, attenuated *S. aureus*-associated mastitis by limiting inflammatory responses and improving the blood-milk barrier disruption in which the underlying mechanism was involved in TGR5-mediated NF-κB and NLRP3 inhibition through the cAMP-PKA pathways. The activation of mammary TGR5 was regulated by gut microbiota, enabling different responses and development to *S. aureus*-induced mastitis, as evidenced by gut dysbiosis caused by vancomycin-impaired mammary TGR5 expression but restored after SFB compensation. Moreover, treatment mice with secondary BA producer also activated mammary TGR5 and alleviated *S. aureus*-induced mastitis in mice.

Host- or microbiota-mediated metabolic changes have been proven to play a central role in the development and outcomes of infectious diseases^3,9,37^. We then performed untargeted metabolomics to identify potential metabolites that affected the susceptibility of pathogen-induced mastitis. The results showed that SARA cows had metabolic profiles in their milk that were distinct from those of healthy cows. Previous studies indicated that SARA increased systemic inflammatory responses and changed metabolites in the circulation^38^, which was associated with reduced milk yield and fat synthesis. Consistently, our results showed that SARA cows had changed metabolites enriched in fatty acid biosynthesis. A recent study also showed that cows with mastitis had different metabolic traits in their serum^39^, leading that mastitis was associated with changed host homeostasis. Of note, gut microbiota-associated BAs, especially CA and DCA, were reduced in SARA cows, which act as important factors for regulating host function and the outcomes of diseases^7,25,40^. Consistently, a previous study indicated that consumption of a fiber-enriched diet increased serum secondary bile acids levels in cows with subclinical mastitis, including DCA and lithocholic acid^41^. Another study showed that mammary glands from Mediterranean diet-fed animals had higher levels of bile acid metabolites, including cholate, glycocholate, taurocholate, deoxycholate and chenodeoxycholate^21^, which suggests that dietary components are a main determinant of secondary bile acids metabolism. In clinical and subclinical mastitis cows, lower serum secondary bile acids including DCA and 12-ketolithocholic acid were also detected^42^. Interestingly, Thomas et al. found a dynamic change in BA concentrations in milk after intramammary challenge with *Streptococcus uberis*^43^, implying the potential role of BAs in pathogen-induced mastitis. In addition, bile salts have been reported to regulate zinc uptake and capsule synthesis in a mastitis-associated pathogenic *E. coli*^44^, suggesting that different bile acids may have different effects on the host or pathogens.

In our study, treatment with DCA, but not CA, alleviated *S. aureus-*induced mastitis in mice. Similar results were found in other disease models in which DCA, but not CA, improved the outcome of autoimmune uveitis in mice^26^. We also confirmed the role of DCA in regulating host responses but not inhibiting bacterial growth since non-antimicrobial dose of DCA also reduced *S. aureus-*induced inflammation. Our results agree with previous findings that a non-antimicrobial dose of DCA has the capacity to limit LPS- or pathogen-induced inflammatory responses in macrophages^24^. DCA treatment restored the decrease in TJ proteins caused by *S. aureus*, suggesting improved blood-milk barrier integrity. In intestinal epithelial cells, DCA was also found to regulate TJ levels^45^. Impaired barrier function is a common response to increased inflammatory responses that are attributed to activated inflammatory signatures, such as the NF-κB and NLRP3 pathways. *S. aureus* invasion has been known to activate the NF-κB and NLRP3 pathways and these signatures play an essential role in the pathogenesis of mastitis^16^. However, DCA depressed the activation of NF-κB and NLRP3 in the MMECs, which agrees with the finding that DCA can inhibit the activation of proinflammatory pathways^24^. DCA can work through multiple host receptors, including TGR5, FXR, and VDR^26^. We then determined that TGR5 was significantly increased after DCA treatment in the mammary gland. In return, blocking TGR5 with SBI-115 reversed the protective effects of DCA. Another study also showed that SBI-115 reversed the effects of DCA or INT777, a specific TGR5 agonist, on autoimmune uveitis^26^. Moreover, increased TGR5 activation regulates the expression of cAMP and subsequent PKA^24,26^. Blocking cAMP and PKA with MDL12330A and H89 impaired the effects of DCA on NF-κB and NLRP3 and subsequent proinflammatory cytokines in the MMECs. These results showed that TGR5-mediated cAMP-PKA pathways contributed to the effects of DCA on *S. aureus-*induced mastitis in mice.

Considering the role of DCA and CA in *S. aureus-*induced mastitis in mice and the gut microbiota in BA metabolism and mastitis^7,25^, we next studied gut microbiota-mediated TGR5 activation in the pathogenesis of mastitis. Mice treated with vancomycin had reduced DCA levels and TGR5 activation and developed more serious mastitis than the control mice. Similar results were also found in a previous study in which depletion of gut obligate anaerobic microbes by clindamycin aggravated *C jejuni*-induced colitis in specific pathogen-free (SPF) IL10^−/−^ mice by reducing intestinal DCA levels^7^. Hu et al. also showed that impairment of the secondary BA reservoir by antibiotics also exacerbated autoimmune uveitis in mice^26^. Supplementation with SFB restored the DCA and TGR5 levels in the mammary glands and ameliorated *S. aureus-*induced mastitis in mice, supporting the essential role of spore-forming *Clostridium* in the production of secondary BAs^25,35^. *C. scindens* increased mammary TGR5 activation compared with the control mice and reduced *S. aureus-*induced mastitis. Another study also showed that *C. scindens* mirrored the role of DCA or TGR5 activation in uveitis^26^. These results suggest that gut microbiota-mediated secondary BA production contributes to protecting against *S. aureus*-induced mastitis in mice. Notably, our results did not allow us to rule out the role of other metabolites in the pathogenesis of *S. aureus*-induced mastitis, as well as gut microbiota-derived host immune changes. In addition, whether the protective effects of gut microbiota-mediated secondary BAs on *S. aureus* are conservative in other organs needs to be proven. Future studies also need to explore the effects and potential mechanisms of other types of primary and secondary BAs on *S. aureus* infection.

In conclusion, our results show that the SARA cows have distinct metabolic changes compared with healthy cows. Among the metabolites potentially affecting the development of mastitis is DCA. Treatment with DCA alleviates *S. aureus-*induced mastitis in mice and the underlying mechanism is involved in the activation of TGR5, which inhibits the activation of NF-κB and NLRP3 signaling by cAMP and PKA. Gut dysbiosis impairs DCA production and TGR5 activation, which aggravates *S. aureus-*induced mastitis in mice, but restoring microbiota by SFB or commensal *C. scindens* ameliorates *S. aureus-*induced mastitis in mice. Our results suggest that gut microbiota-mediated secondary acid production is a key regulator in the pathogenesis of *S. aureus*-associated mastitis and acts as a basis for regulating gut microbiota-mediated metabolism to treat mastitis and other diseases.

## Methods

### Ethical statement

All animal experiments were subject to approval by the Institutional Animal Care and Use Committee (IACUC) of Jilin University (China). The full proposal was considered by the IACUC ethics committee, which approved the animal care and use permit license. All experiments complied with the manual of the care and use of laboratory animals published by the US National Institutes of Health.

### Materials

Deoxycholic acid (DCA), cholic acid (CA), and vancomycin were bought from Sigma Aldrich (St. Louis, MO, USA). SBI-115, MDL-12330A, and H89 were purchased from MedChemExpress (MCE, USA). The primary antibodies, including phosphorylation-p65 (p-p65, #AF2006; RRID: AB_2834435), p-65 (#AF5006; RRID: AB_2834847), p-IκB (#AF2002; RRID: AB_2834433), IκB (#AF5002; RRID: AB_2834792), Occludin (#DF7504; RRID: AB_2841004), ZO-1 (#AF5145; RRID: AB_2837631), Claudin-3 (#AF0129; RRID: AB_2833313) and β-actin (#AF7018; RRID: AB_2839420) were obtained from Affinity Biosciences (OH, USA). NLRP3 (#15101), ASC (#67824), and IL-1β (#12242) were bought from Cell Signaling Technology (CST, Boston, USA). Goat anti-rabbit or Rabbit anti-mouse secondary antibodies were bought from ImmunoWay Biotechnology Company. Mouse TNF-α (Cat #430915) and IL-1β (Cat #432615) ELISA assay kits were obtained from Biolegend (CA, USA). Myeloperoxidase (MPO) assay kit was bought from Nanjing Jiancheng Bioengineering Institute (Nanjing, China).

### Cow treatment and samples collection

A total of 12 Holstein cows (4–6 years, averaging ~600 kg of weight) were obtained from a farm in Qingzhou, Shangdong Province, China, and none of the animals had diseases or were treated with antibiotics or drugs within 6 months. Cows were randomly separated into healthy group and SARA group. The healthy group was treated with a standard diet of grass-legume hay and the SARA group was treated with a high-concentrate diet (70% grain diet)^20,46^. All cows met the daily nutrient requirements for lactation. After eight weeks of treatment, milk samples from the different treatment groups were harvested and stored in liquid nitrogen until the metabolomics analysis. The ruminal pH was used to diagnose SARA^47^.

### Untargeted metabolomics

The milk samples (100 mL) were mixed with prechilled methanol (400 μL) by vortexing. The samples were incubated on ice for 5 min and then centrifuged at 15,000 × *g* and 4 °C for 5 min. The supernatant was diluted to a final concentration containing 53% methanol with LC-MS grade water. The samples were subsequently transferred to a fresh Eppendorf tube and then were centrifuged at 15,000 × *g* and 4 °C for 10 min. Finally, the supernatant was injected into the LC-MS/MS system for analysis.

The LC-MS/MS analyses were performed using a Vanquish UHPLC system (Thermo Fisher) coupled with an Orbitrap Q Exactive series mass spectrometer (Thermo Fisher). Samples were injected onto a Hyperil Gold column (100 × 2.1 mm, 1.9 μm) using a 16 min linear gradient at a flow rate of 0.2 mL/min. The raw data files generated by the UHPLC-MS/MS were processed using Compound Discoverer 3.1 (Thermo Fisher) to perform peak alignment, peak picking, and quantitation for each metabolite. The main parameters were set as follows: retention time tolerance, 0.2 min; actual mass tolerance, 5 ppm; signal intensity tolerance, 30%; signal/noise ratio, 3; and minimum intensity, 100000. After the peak intensities were normalized to the total spectral intensity. The normalized data were used to predict the molecular formula based on additive ions, molecular ion peaks, and fragment ions. Then, peaks were matched with the mzCloud (https://www.mzcloud.org/), mzVault, and MassList databases to obtain accurate qualitative and relative quantitative results. Statistical analyses were performed using the statistical software R (R version R-3.4.3), Python (Python 2.7.6 version) and CentOS (CentOS release 6.6), When data were not normally distributed, normal transformations were attempted using the area normalization method.

These metabolites were annotated using the KEGG database (http://www.genome.jp/kegg/) and Lipidmaps database (http://www.lipidmaps.org/). The PCA and PLS‐DA were performed at metaX. Pearson correlation analysis was used to assess the correlation of QC samples to determine the stability and accuracy of the metabolome. We applied univariate analysis (t-test) to calculate the statistical significance (*P*-value). Metabolites with a VIP > 1 and *P*-value < 0.05 and fold change ≥2 or FC ≤ 0.5 were considered to be differential metabolites. Volcano plots were used to filter metabolites of interest based on the Log2 (FC) and -log10 (*P*-value) of the metabolites. For the clustering heat maps, the data were normalized using z-scores of the intensity areas of differential metabolites and were plotted by the Pheatmap package in R language. The functions of these metabolites and metabolic pathways were studied using the KEGG database. The metabolic pathway enrichment of the differential metabolites was performed. When the ratio was satisfied by x/n > y/N, the metabolic pathways were considered enriched. When the *P*-value of metabolic pathway < 0.05, the metabolic pathways were considered as significantly enriched.

### Mouse and treatments

All the SPF-grade BABL/c mice (6–8 weeks, 22–24 g) were obtained from Liaoning Changsheng Biotecnology Co., Ltd. (Benxi, China). The mice were raised with enough food and water in SPF-grade feeding conditions with 12 h light and 12 h dark daily for a week. After adapting to the feeding environment, these mice were mixed at a ratio of three females to one male in separated cages with the same feeding conditions. After confirming pregnancy by the observation of vaginal spermatozoa, the male mice were removed.

For the DCA supplementation experiment, mice 7 days after delivery were pretreated with DCA (10, 20, and 30 mg/kg) for 2 h intraperitoneally before the induction of the *S. aureus*-induced mastitis model^24^. To confirm the role of gut microbiota in converting CA to DCA and regulating mastitis pathogenesis, mice were treated with CA (30 mg/kg) or DCA (30 mg/kg) orally with or without antibiotics treatment (200 mg/kg ampicillin, neomycin and metronidazole, 100 mg/kg vancomycin) for a week. For the vancomycin treatment experiment, mice were treated with vancomycin (0.5 g/L) in the drinking water for three weeks. For the *C. scindens* treatment, mice were treated with antibiotics (200 mg/kg ampicillin, neomycin, and metronidazole, 100 mg/kg vancomycin) for 5 days to deplete the commensal microbiota^48^ and *C. scindens* (10^9^ CFU/mouse) was supplemented by oral gavage for 7 consecutive days^26^.

### Bacterial spore preparation and transplantation

For SFB transplantation experiment, the cecal contents were harvested from the conventionally fed mice under sterile conditions and the cecal contents were added to a 1.5 mL microcentrifuge tube on ice. The cecal contents were diluted 1:10 in PBS (w/v) and chloroform was added at a final concentration of 3% (v/v). The prepared cecal contents were next incubated by shaking at 200 × g at 37 °C for 30 min. After allowing the chloroform to settle to the bottom of the tube at room temperature (approximately 20 min), the top aqueous layer was removed and the SFB was resuspended with 200 μL PBS in a sterile 1.5 mL tube^35,49^. For transplantation, mice were pretreated with vancomycin for 14 days and then treated with 200 μL SFB daily for consecutive 7 days.

### *S. aureus*-induced mastitis model

Lactating mice seven days after delivery were separated from the offspring for three hours and subsequently anesthetized using urethane (100 mg/kg). Next, these mice were treated with *S. aureus* USA300 (10^7^ CFU/50 μL) by intraductal injection at the fourth nipple^16,50^. Twenty-four hours later, the mammary tissues were harvested and stored at −80 °C until determination.

### Bacterial culture and growth assay

The *S. aureus* USA300 was cultured in a tryptone soybean broth (TSB) medium at 37 °C. The *C. scindens* ATCC35704 was purchased from American Type Culture Collection (ATCC) and cultured under anaerobic conditions. To investigate the effect of DCA and CA on bacterial growth, the *S. aureus* USA300 was incubated in TSB medium supplemented with DCA (0, 25.47, 63.68, 127.36, 191.04, 254.73, 636.82, 1273.65, 1910.48 and 2547.31 μM) or CA (0, 24.47, 61.18, 122.37, 183.56, 244.75, 611.89, 1223.78, 1835.67 and 2447.56 μM) and bacterial intensities were measured at 600 nm optical density (OD600) after incubation at 37 °C for 24 h.

### Biofilm assay

To investigate whether DCA affects the formation of *S. aureus* biofilms, *S. aureus* was incubated in a TSB medium in 96-well plates supplemented with different concentrations of DCA (0, 25.47, 63.68, 127.36, 191.04, 254.73, 636.82, 1273.65, 1910.48 and 2547.31 μM) or CA (0, 24.47, 61.18, 122.37, 183.56, 244.75, 611.89, 1223.78, 1835.67 and 2447.56 μM) at 37 °C for 24 h without shaking. After incubation, the supernatants were removed carefully and the biofilm in the plate was stained with 1% crystal violet solution for 10 min. After washing with PBS three times every 2 min, the plate was dried at 72 °C for 2 h. Finally, 200 μL ethanol (75%) was added to each well and bacterial biofilm was detected at OD570^51^.

### Cell culture and inflammatory response assay

Mouse mammary epithelial cells (MMECs, HC11 cells) were obtained from the American Type Culture Collection (ATCC, CRL-3062) and cultured in a DMEM supplemented with 10% fetal bovine serum and 1% ampicillin and streptomycin at 37 °C with 5% CO_2_. For the BA treatment experiment, ampicillin and streptomycin were removed from the cells (10^6^ cells/mL) and incubated in 6-well plates for 24 h. The prepared cells were then treated with SBI115 (100 μM), KH7 (10 μM), MDL12330A (10 μM), or H89 (30 μM) for 2 h prior to DCA. For the DCA treatment, DCA was performed 2 h prior to *S. aureus* treatment with a final concentration of 10, 20, or 30 μM according to the experimental demand. For the siRNA transfection experiment, TGR5 siRNA, cAMP siRNA, PKA siRNA, and the negative control siRNA were transfected using Opti-MEM and Lipofectamine^52^. All siRNAs were diluted in RNase-free water to a final concentration of 40 nM. The cells were incubated with the DMEM without antibiotics and serum for 24 h followed by a complete medium incubation for the next 24 h. DCA was added 48 h post-siRNA infection. The prepared cells were further treated with *S. aureus* (MOI 100:1) for 24 h according to the results of the preliminary experiments and the cells were harvested for determination.

### Histological analysis

All samples used for the histological analysis were fixed with 4% paraformaldehyde for more than 48 h and embedded in paraffin to prepare 5-μm paraffin sections. All sections were stained with hematoxylin and eosin (H&E) after dewaxing and hydration. The histological changes in the mammary glands were performed by using an optical microscope (Olympus, Tokyo, Japan) and the histological score was assessed^17^.

### Cytokine detection

ELISA assay was performed to determine the predominant proinflammatory cytokines of TNF-α and IL-1β in the serum, mammary gland, and cultured cell supernatant. For the mammary samples, 10% tissue homogenates were prepared using PBS and the supernatants were collected by centrifugation at 12,000 × *g* for 10 min. The concentrations of TNF-α and IL-1β the mammary gland were calculated according to the manufacturer’s instructions (Biolegend, CA, USA).

### MPO activity assay

To determine the MPO levels, 10% tissue homogenates were prepared using a MPO buffer and detected by a MPO assay kit (A044-1-1) according to the manufacturer’s instructions (Nanjing Jiancheng Bioengineering Institute, Nanjing, China).

### DCA and CA analysis

To detect the levels of mammary CA and DCA, mammary samples were pre-weighted in lysis tubes containing ceramic beads and added Methanol (MeOH)-containing internal standard. MeOH-extracted samples were centrifuged, diluted 1:1 in 50% MeOH:water, and injected into the UPLC–MS^52^. CA and DCA levels were determined using commercially available standards purchased from Sigma.

### Total bacterial DNA extraction and sequencing

For the gut microbiome analysis, fecal samples were collected from the individual mice. Microbial genomic DNA was extracted by using the FastDNA® Spin Kit for Soil (MP Biomedicals, U.S.). 16 S rRNA gene libraries were constructed using primers specific to the V3-V4 region (338 F (5′-ACTCCTACGGGAGGCAGCAG-3′) and 806 R (5′-GGACTACHVGGGTWTCTAAT-3′)) by an ABI GeneAmp® 9700 PCR thermocycler (ABI, CA, USA) and performed paired-end sequencing on an Illumina MiSeq PE300 platform/NovaSeq PE250 platform (Illumina, San Diego, USA) by Majorbio Bio-Pharm Technology Co. Ltd. (Shanghai, China). Operational taxonomic units (OTUs) with a 97% similarity cutoff were clustered using UPARSE version 7.1, and chimeric sequences were identified and removed. The taxonomy of each OTU representative sequence was analyzed by RDP Classifier version 2.2 against the 16S rRNA database using a confidence threshold of 0.7. The principal coordinate analysis was performed to identify the separation of gut microbiota among the different treatment groups based on the Bray-Curtis distance and significance was analyzed by ANOSIM. LEfSe was used to identify the differential bacterial taxa in the different treatment groups (LDA score (log10) > 3).

### Total RNA extraction and quantitative RT-PCR

The total RNA of the mammary tissues was extracted by Trizol (Invitrogen, CA, USA)^17^. Briefly, 100 mg of tissues or collected cells were extracted with 1 mL Trizol and subjected to chloroform, isopropanol, and 75% ethyl alcohol treatment under RNase-free conditions. After reverse transcription using TransStart Tip Green qPCR SuperMix (TransGen Biotech, Beijing, China), the cDNA was reacted with specific primers using a FastStart Universal SYBR Green Master Mix (ROX) (Roche, Switzerland, Basel) in a Step One Plus apparatus (Applied Biosystems, Foster City, CA, USA). The reaction conditions were performed as previously mentioned^17^. The primers used in this study are presented in Supplementary Table 4, and GAPDH served as an endogenous control. The 2^−ΔΔCt^ method was performed to calculate the relative expression of genes by calibration with the control group.

### Western blotting

The total protein of the mammary gland was harvested using a tissue protein extraction kit (Thermo Fisher Scientific, USA)^17^. Ten percent or 15% SDS-PAGE was used to separate proteins, which were then bound to 0.45 μm PVDF membranes. After blocking in 5% skim milk, the prepared PVDF membranes were incubated at 4 °C overnight with specific primary antibodies, including p-p65 (#AF2006; 1:1000), p-65 (#AF5006; 1:1000), p-IκB (#AF2002; 1:1000), IκB (#AF5002; 1:1000), Occludin (#DF7504; 1:1000), ZO-1 (#AF5145; 1:1000), Claudin-3 (#AF0129; 1:1000), NLRP3 (#15101; 1:1000), ASC (#67824; 1:1000), IL-1β (#12242; 1:1000) and β-actin (#AF7018; 1:1000). After washing three times with TBST, the PVDF membranes were treated with goat anti-rabbit or rabbit anti-mouse IgG (1:20000) for 2 h at room temperature. Finally, the proteins were detected using the ECL plus western blotting Detection System (Tanon, China). All blots or gels derive from the same experiment and that were processed in parallel. Original blots are provided in Supplementary Information.

### Statistical analysis

All data were analyzed using GraphPad Prism 8 (San Diego, CA, USA) and expressed as boxplots or the means ± SD. The Student’s t test (parametric) and Mann-Whitney test (nonparametric) were performed for the comparison of two groups. One-way analysis of variance (ANOVA) was performed for the comparison of more than two groups, followed by Tukey’s test to determine the differences among groups. A *p* < 0.05 indicated statistical significance. Other special analyses are stated in the legends.

### Reporting summary

Further information on research design is available in the Nature Research Reporting Summary linked to this article.

## Supplementary information

Supplementary information

Reporting Summary

## Data Availability

The 16S rRNA gene sequencing data are available in NCBI Sequence Read Archive (SRA) repository under accession number PRJNA892063.
