# Supplementary material for: Gut microbiota-mediated secondary bile acid alleviates Staphylococcus aureus-induced mastitis through the TGR5-cAMP-PKA-NF-κB/NLRP3 pathways in mice
Source: NPJ Biofilms Microbiomes. 2023 Feb 8;9:8. doi: 10.1038/s41522-023-00374-8 (PMC9908919; doi:10.1038/s41522-023-00374-8)

Supplementary materials for

Gut microbiota-mediated secondary bile acid alleviates *Staphylococcus aureus*-induced mastitis through the TGR5-cAMP-PKA-NF-κB/NLRP3 pathways in mice

Caijun Zhao[1#], Keyi Wu[1#], Haoyang Hao[1], Yihong Zhao[1], Lijuan Bao[1], Min Qiu[1], Yuhong He[1], Zhaoqi He[1], Naisheng Zhang[1], Xiaoyu Hu[1]*, Yunhe Fu[1]*

1. Department of Clinical Veterinary Medicine, College of Veterinary Medicine, Jilin University, Changchun, Jilin Province 130062, China.

# Authors contributed equally to this study.

*** Corresponding author:**

Yunhe Fu, E-mail: fuyunhesky@sina.com.

Xiaoyu Hu, E-mail: hxiaoyu@yeah.net.

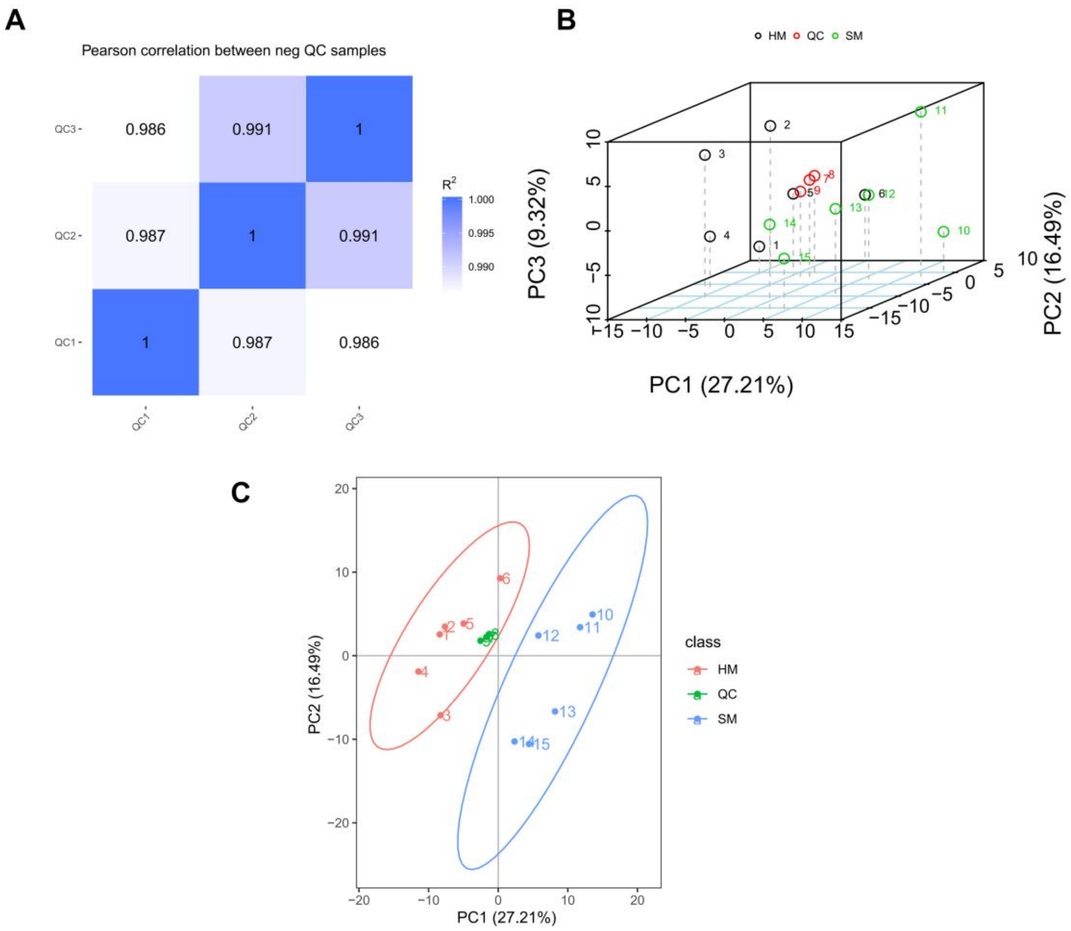

Supplementary Figure 1. Data quality checks. A. The Pearson correlation of milk QC samples. **B-C**. The PCA score plots for the healthy and SARA samples containing QC samples. QC, quality control; Principal component analysis, PCA

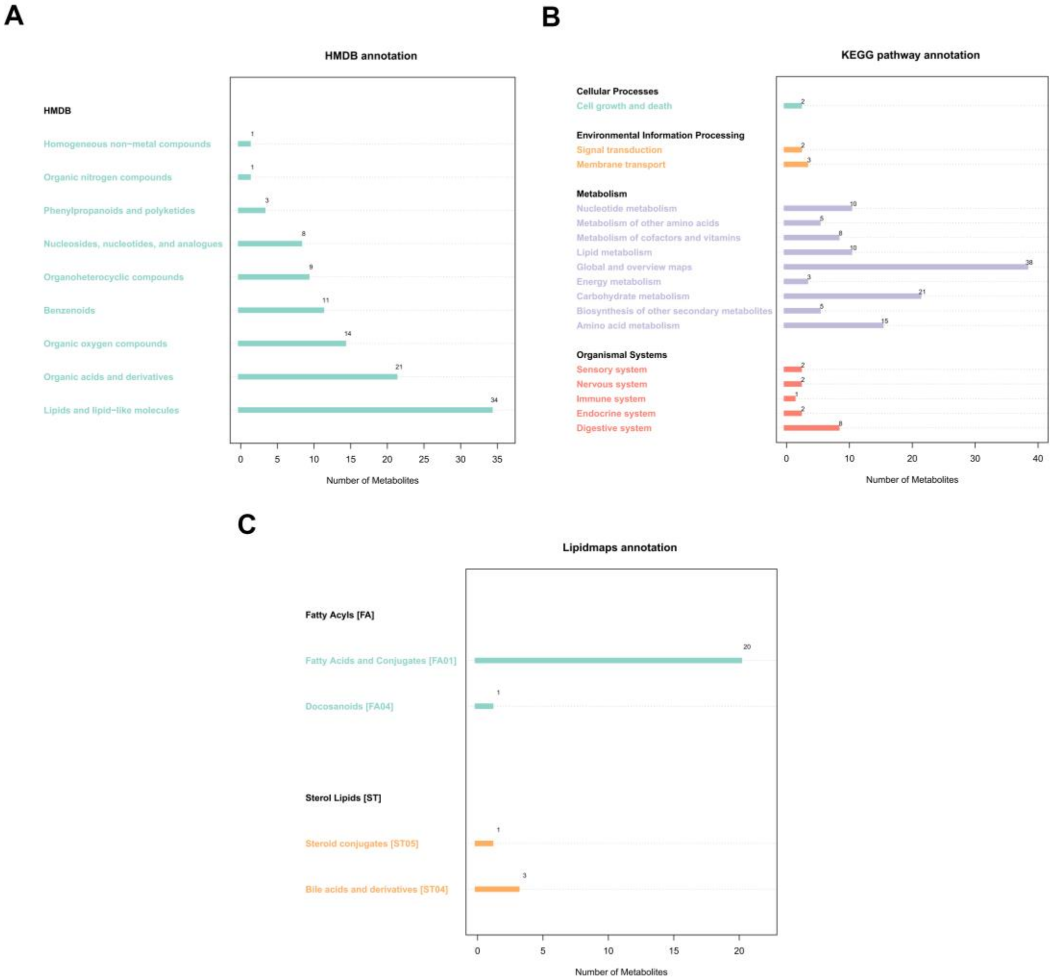

Supplementary Figure 2. Classification and functional annotation of metabolites. A. HMDB classification annotation. **B.** KEGG pathway annotation for the healthy and SARA milk samples. **C**. Lipid maps annotation for the healthy and SARA samples. HMDB, Human Metabolome Database; KEGG, Kyoto Encyclopedia of Genes and Genomes.

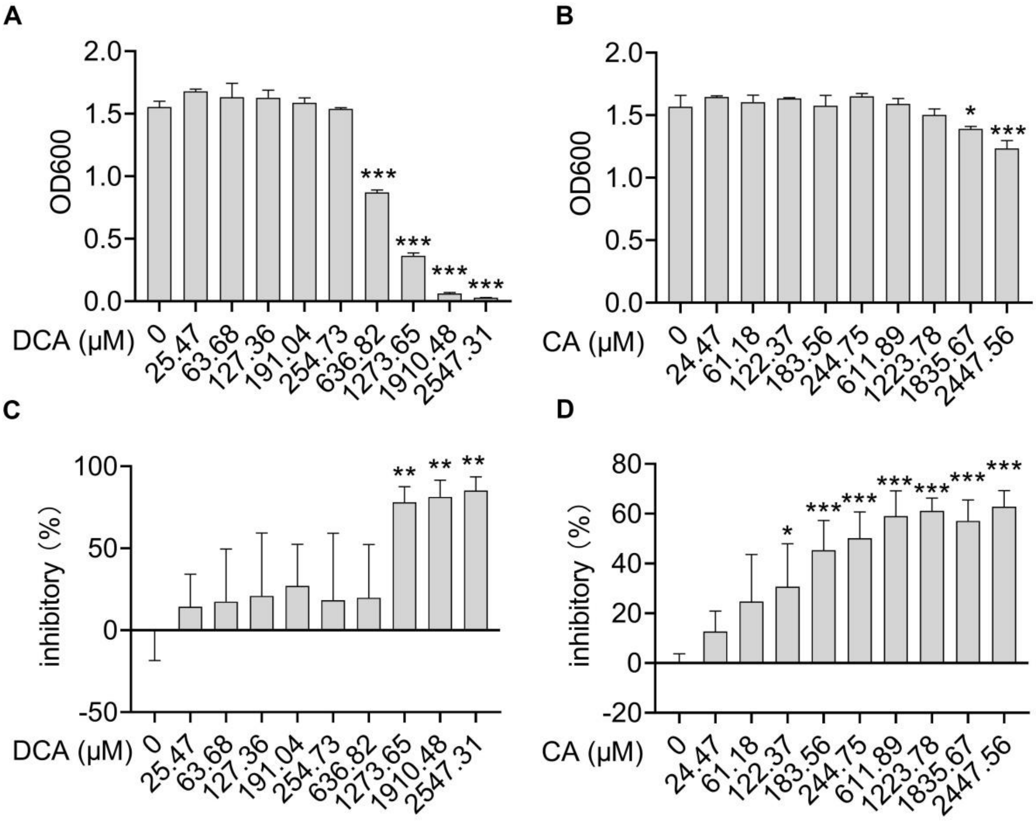

Supplementary Figure 3. The effects of DCA and CA on *S. aureus* growth and biofilm formation. A-B. *S. aureus* was cultured in a TSB supplemented with different concentration of DCA or CA for 24 h and measured at OD 600 nm. **C-D**. *S. aureus* was cultured in a TSB supplemented with different concentration of DCA or CA for 24 h without shaking. The biofilm was detected at OD 570 nm after crystal violet staining. Data are expressed as the means ± SD (**A-D**). **p* < 0.05, ***p* < 0.01, ****p* < 0.001 indicate significance by Student's t-test.

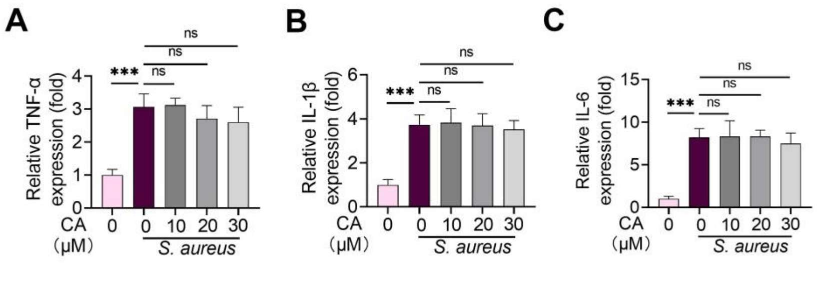

Supplementary Figure 4. CA does not alleviate *S. aureus*-induced inflammatory response in MMECs. Cells were pretreated with CA (10, 20 and 30 μM) for 2 h followed by *S. aureus* treatment for next 24 h, and the relative mRNA levels of proinflammatory TNF-α (**A**), IL-1β (**B**) and IL-6 (**C**) from the indicated group were detected by qPCR. Data are expressed as the means ±

SD (**A-C**) and one-way ANOVA followed by Tukey's test (**A-C**) was performed. ***p < 0.001 indicates significance. ns, no significance.

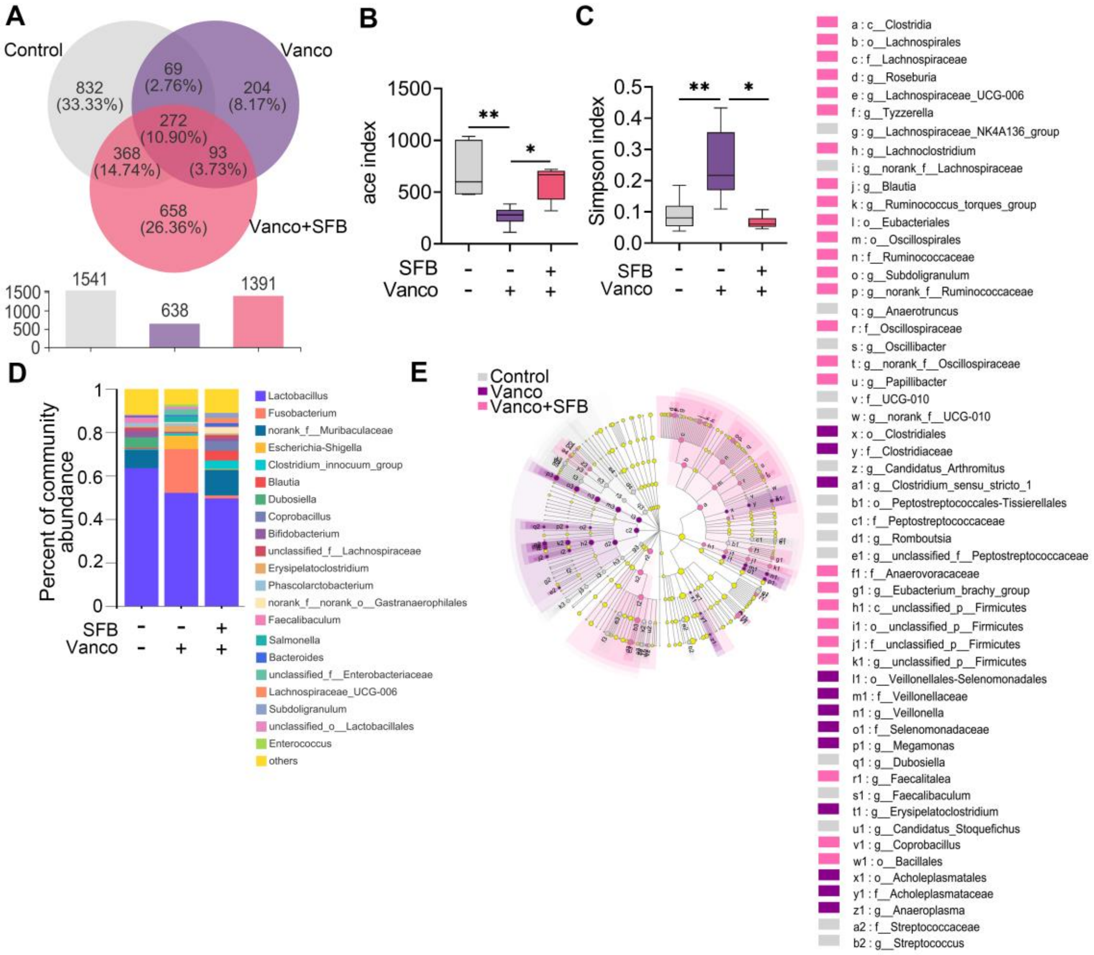

Supplementary Figure 5. The gut microbiota profiles after vancomycin treatment and SFB transplantation. A. Venn diagram from the indicated groups. **B-C**. Alpha diversity analysis by ace and Simpson indices. **D.** Gut microbial compositions at the genus level from the indicated groups. **E**. LEfSe showed different taxa that enriched in the indicated groups. Data are expressed as boxplots, with the center line representing the median, the boundary of the whiskers representing the minimum and maximum values of the dataset, and the boundary of the box representing the 25th and 75th percentile of the dataset (**B-C**) and one-way ANOVA followed by Tukey's test (**B-C**) was performed. *p < 0.05 and **p < 0.01 indicate significance.

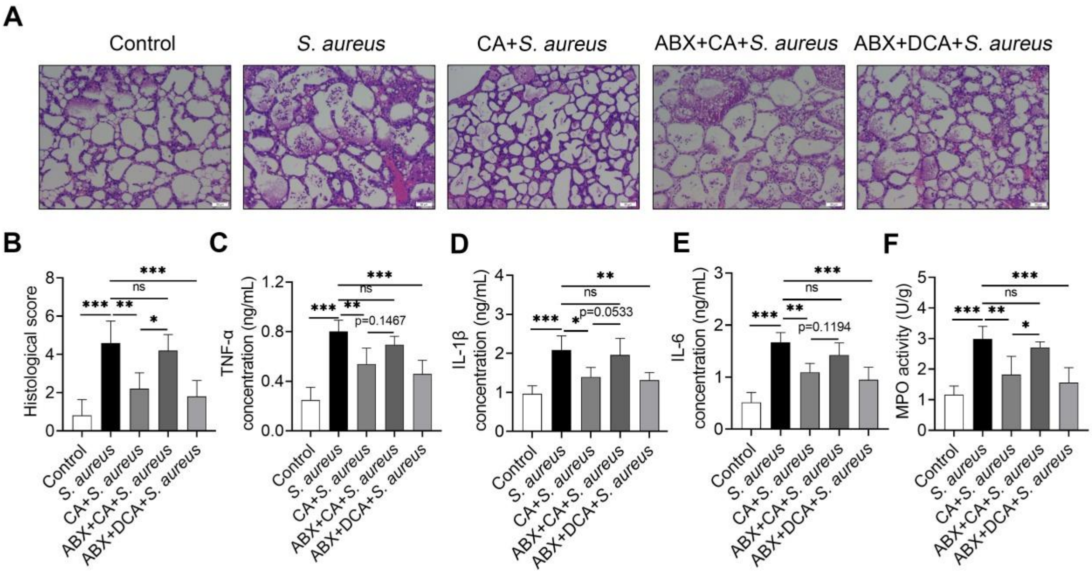

Supplementary Figure 6. Gut microbiota-mediated DCA production alleviates S. aureus-induced mastitis in mice. Mice were treated with CA (30 mg/kg) or DCA (30 mg/kg) for a week with or without antibiotics treatment, followed by *S. aureus*-induced mastitis. **A**. Representative H&E-stained sections of the mammary gland from the indicated groups (scale bar, 50 μm). **B**. Histological score for the mammary gland based on H&E-stained sections (n=6). Inflammatory parameters of the mammary gland from different groups, including TNF-α (**C**), IL-1β (**D**) and IL-6 (**E**) concentrations and MPO activity (**F**), were performed (n=6). Data are expressed as the means ± SD (**B-F**) and one-way ANOVA was performed, followed by Tukey's test (**B-F**). **p* < 0.05, ***p* < 0.01, ****p* < 0.001 indicate significance. ns, no significance.

Supplementary Table 1. Identified number of differential metabolites in Health and SARA samples.

Compared Sample	Number of total identified	Number of total significant	Number of significant Up	Number of significant Down
SARA. vs. Healthy	213	44	5	39

Supplementary Table 2. Metabolites significantly upregulated in SARA cows and ranked according to the P-value

Name	Formula	FC	log2FC	Pvalue	VIP
1-methyl-N-(3-methyl-5-cinnolinyl)-1H-imidazole-4-sulfonamide	C13 H13 N5	3.325244	1.73346	0.028923	1.768183

	O2 S				
3-(methylsulfanyl)-5H-[1,2,4]triazino[5,6-b]indole	C10 H8 N4 S	4.365467	2.126136	0.013204	3.064469
Cinnamoylglycine	C11 H11 N O3	2.173313	1.119896	0.004915	1.270383
Epinephrine	C9 H13 N O3	3.231945	1.692403	4.10E-07	1.872821
N-(1,1-Dioxotetrahydro-1H-1λ6-thiophen-3-yl)-4-methoxybenzamide	C12 H15 N O4 S	9.397567	3.232287	3.13E-07	3.506554

FC, fold change; VIP, variable importance in the projection

Supplementary Table 3. Metabolites significantly downregulated in SARA cows and ranked according to the P-value

Name	Formula	FC	log2FC	Pvalue	VIP
LPE 14:0	C19 H40 N O7 P	0.508115	-0.97677	0.039932	1.193835
SM (d14:1/14:0)	C33 H67 N2 O6 P	0.201858	-2.30859	0.034871	1.77633
Cholic acid	C24 H40 O5	0.122035	-3.03464	0.032836	2.196008
Deoxycholic acid	C24 H40 O4	0.271848	-1.87913	0.031931	1.685549
FAHFA (18:1/20:3)	C38 H66 O4	0.561696	-0.83214	0.020737	1.019588
FAHFA (18:1/18:2)	C36 H64 O4	0.533397	-0.90672	0.012568	1.004694
PS (18:0/22:4)	C46 H82 N O10 P	0.37562	-1.41265	0.011578	1.77248
FAHFA (18:1/19:2)	C37 H66 O4	0.511839	-0.96624	0.010798	1.214777
(R)-3-Hydroxy myristic acid	C14 H28 O3	0.350622	-1.51201	0.010547	1.886358
LPG 18:1	C24 H47 O9 P	0.477809	-1.06549	0.010331	1.251726
Eicosapentaenoic acid	C20 H30 O2	0.498155	-1.00533	0.008601	1.061365
LPE 22:4	C27 H48 N O7 P	0.398686	-1.32667	0.004021	1.367966
cis-2-Decenoic acid	C10 H18 O2	0.315008	-1.66654	0.00175	1.843293
Heptanoic acid	C7 H14 O2	0.304685	-1.71461	0.001718	1.773045

α-Linolenic acid	C18 H30 O2	0.466553	-1.09989	0.001202	1.193713
γ-Linolenic acid	C18 H30 O2	0.466812	-1.09909	0.001187	1.192717
FAHFA (16:0/18:2)	C34 H62 O4	0.393212	-1.34662	0.001101	1.518628
LPS 22:4	C28 H48 N O9 P	0.309656	-1.69126	0.00103	1.753157
Pentadecanoic acid	C15 H30 O2	0.435843	-1.19812	0.000809	1.345737
FAHFA (16:1/18:3)	C34 H58 O4	0.138398	-2.85311	0.000529	3.184504
Ethyl myristate	C16 H32 O2	0.51544	-0.95612	0.000362	1.048398
Adrenic acid	C22 H36 O2	0.244925	-2.02959	0.000346	2.273533
Hexanoic acid	C6 H12 O2	0.359582	-1.47561	0.00034	1.634762
FAHFA (18:2/18:2)	C36 H62 O4	0.214538	-2.2207	0.000328	2.543546
FAHFA (14:0/16:2)	C30 H54 O4	0.149794	-2.73895	0.000284	3.079546
13-Hotre(R)	C18 H30 O3	0.461302	-1.11622	0.000275	1.1945
16-Hydroxyhexadecanoic acid	C16 H32 O3	0.335242	-1.57672	0.00027	1.689894
Tridecylic acid	C13 H26 O2	0.260906	-1.9384	0.000245	2.165901
Arachidonic acid	C20 H32 O2	0.302893	-1.72312	0.000216	1.811439
FAHFA (18:2/20:4)	C38 H62 O4	0.110065	-3.18357	0.000193	3.578785
(+/-)12(13)-DiHOME	C18 H34 O4	0.319267	-1.64717	0.000178	1.875846
11(Z),14(Z)-Eicosadienoic acid	C20 H36 O2	0.392605	-1.34885	0.000129	1.46567
Lauric acid ethyl ester	C14 H28 O2	0.356037	-1.4899	0.000116	1.609447
all-cis-4,7,10,13,16-Docosapentaenoic acid	C22 H34 O2	0.340989	-1.5522	8.80E-05	1.655177
(+/-)-epi CP 47, 497	C21 H34 O2	0.495384	-1.01338	8.31E-05	1.11762
Lauric acid	C12 H24 O2	0.271224	-1.88245	8.25E-05	2.059585

Decanoic acid	C10 H20 O2	0.2722	-1.87726	6.27E-05	2.048578
FAHFA (18:2/17:2)	C35 H60 O4	0.192958	-2.37364	2.42E-05	2.609206
8Z,11Z,14Z-Eicosatrienoic acid	C20 H34 O2	0.349266	-1.5176	6.78E-06	1.655427

Supplementary Table 4. The oligonucleotides used in this study.

Gene	Primer	Sequence(5′ to 3′)
TNF-α	sense	5′- CCCTCACACTCAGATCATCTTCT-3′
	antisense	5′- GCTACGACGTGGGCTACAG-3′
IL-1β	sense	5′- GCAACTGTTCCTGAACTCAACT-3′
	antisense	5′-ATCTTTTGGGGTCCGTCAACT-3′
IL-6	sense	5′- TAGTCCTTCCTACCCCAATTTCC-3′
	antisense	5′-TTGGTCCTTAGCCACTCCTTC-3′
TGR5	sense	5′- CCTGGCAAGCCTCATCGTC-3′
	antisense	5′- AGCAGCCCGGCTAGTAGTAG-3′
CAR	sense	5′- CCCTGACAGACCCGGAGTTA-3′
	antisense	5′- GCCGAGACTGTTGTTCCATAAT-3′
VDR	sense	5′- ACCCTGGTGACTTTGACCG-3′
	antisense	5′- GGCAATCTCCATTGAAGGGG-3′
GR	sense	5′- AGCTCCCCCTGGTAGAGAC-3′
	antisense	5′- GGTGAAGACGCAGAAACCTTG-3
FXR	sense	5′-GCTTGATGTGCTACAAAAGCTG-3′
	antisense	5′- CGTGGTGATGGTTGAATGTCC-3
PXR	sense	5′- GATGGAGGTCTTCAAATCTGCC-3′
	antisense	5′- GGCCCTTCTGAAAAACCCCT-3
α5β1	sense	5′-ATGCCAAATCTTGCGGAGAAT-3′
	antisense	5′- TTTGCTGCGATTGGTGACATT-3
GAPDH	sense	5′-AACTTTGGCATTGTGGAAGG-3′
	antisense	5′-ACACATTGGGGGTAGGAACA-3′

Original blots presented in the manuscript

Fig 2N ZO-1

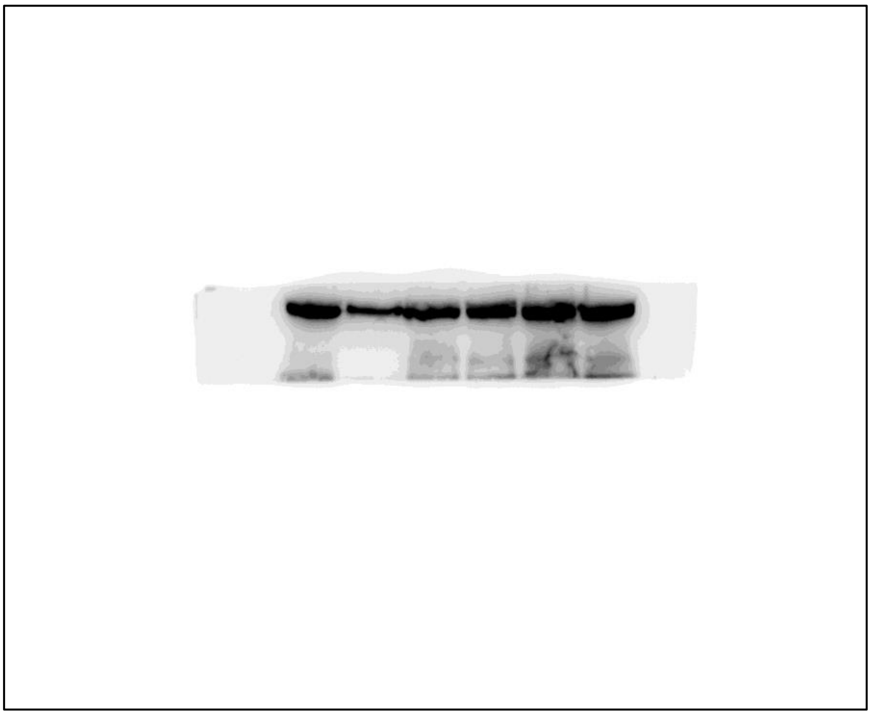

Fig 2N Occludin

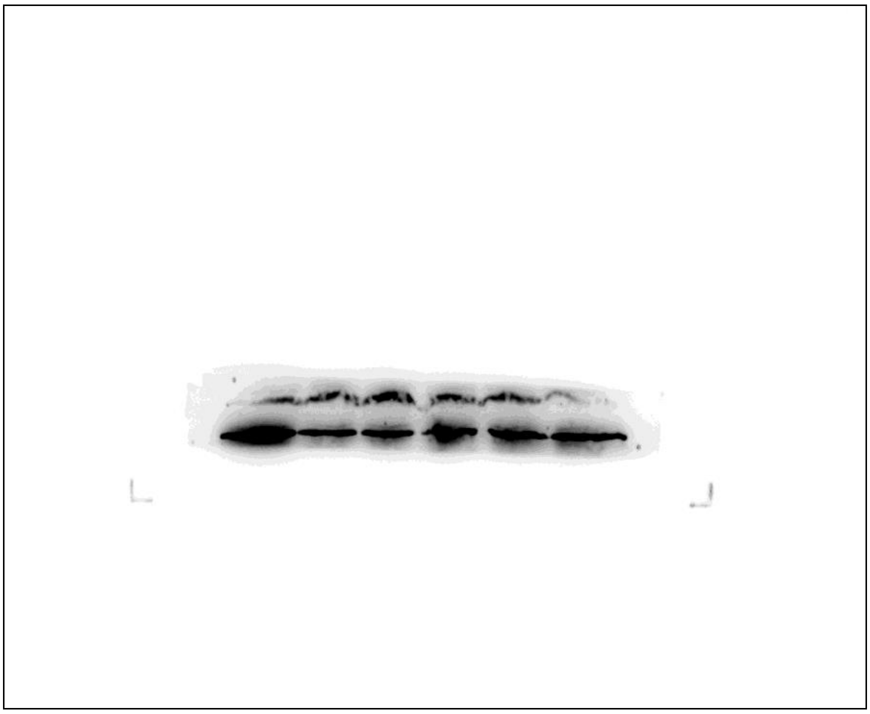

Fig 2N Claudin-3

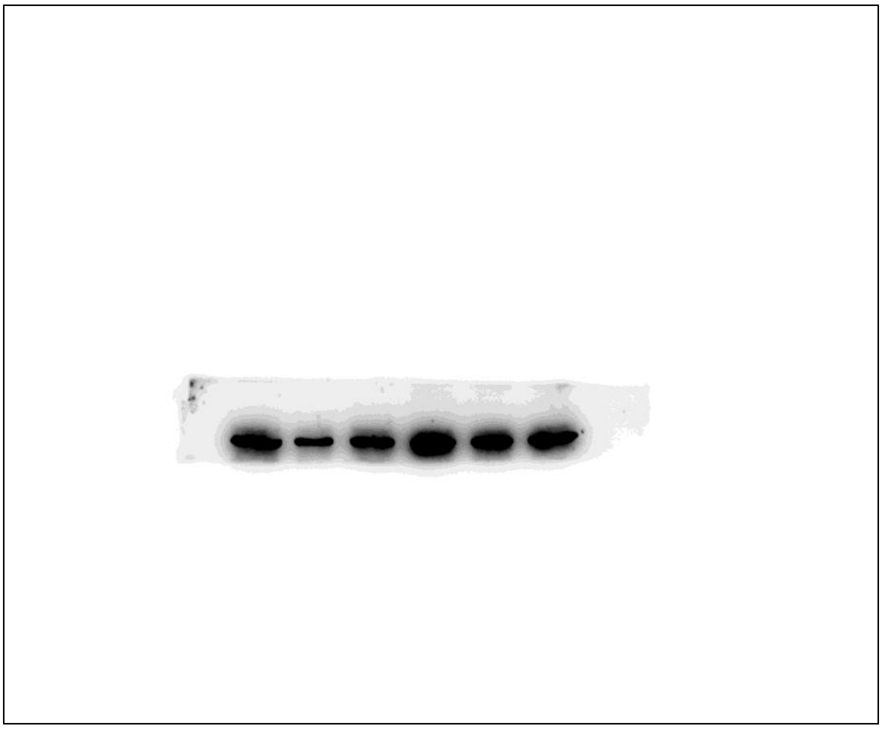

Fig 2N actin

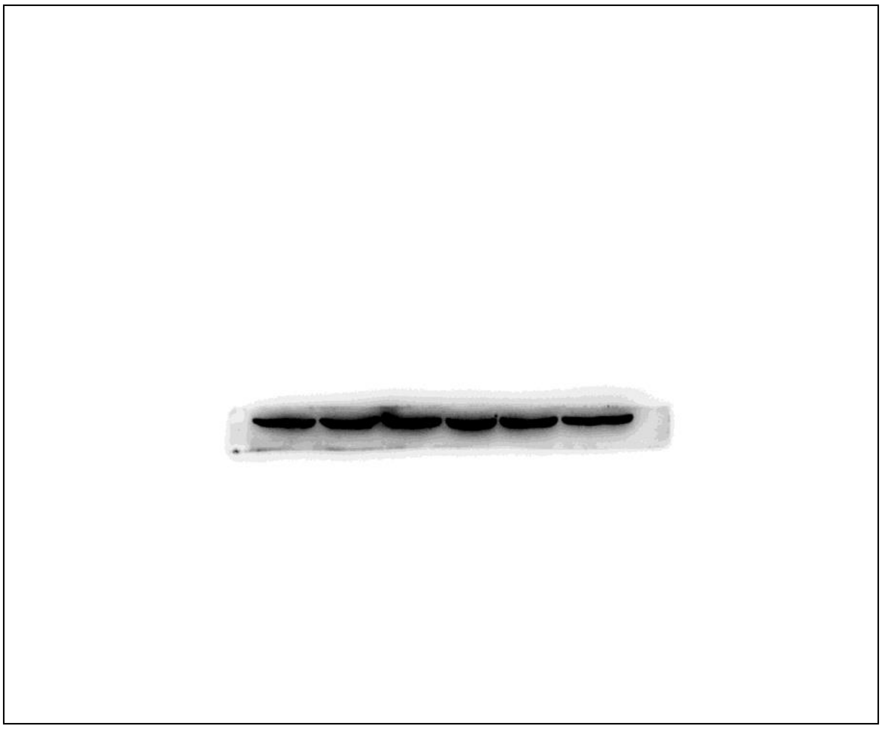

Fig 4A NLRP3

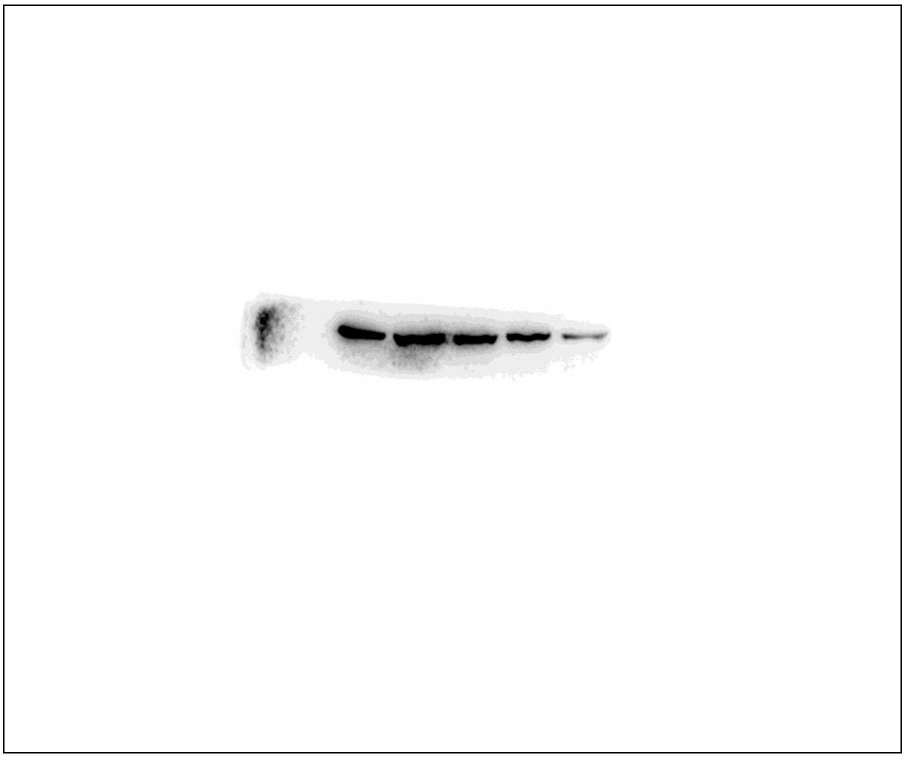

Fig 4A ASC

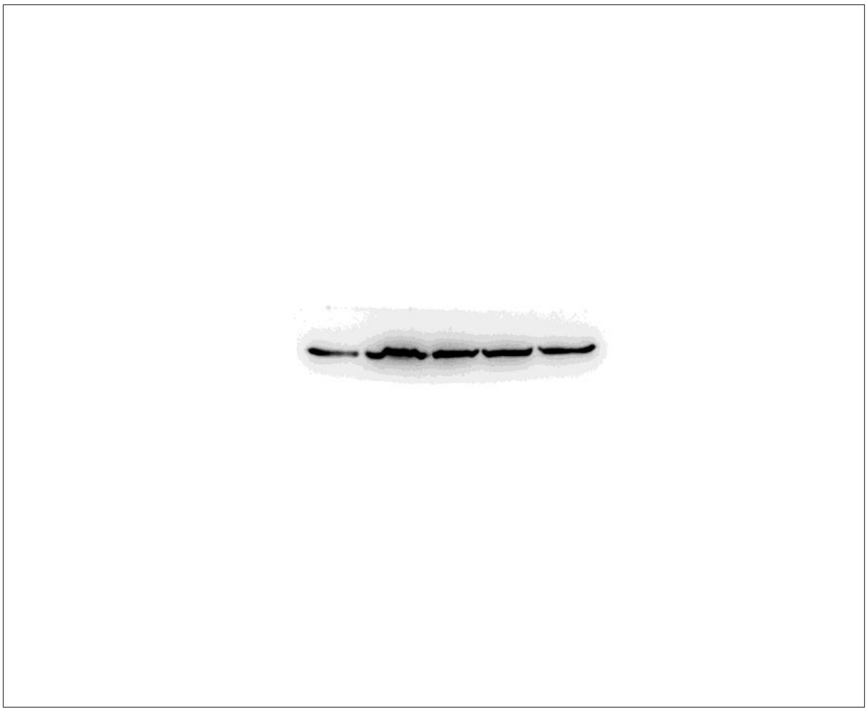

Fig 4A Caspase-1

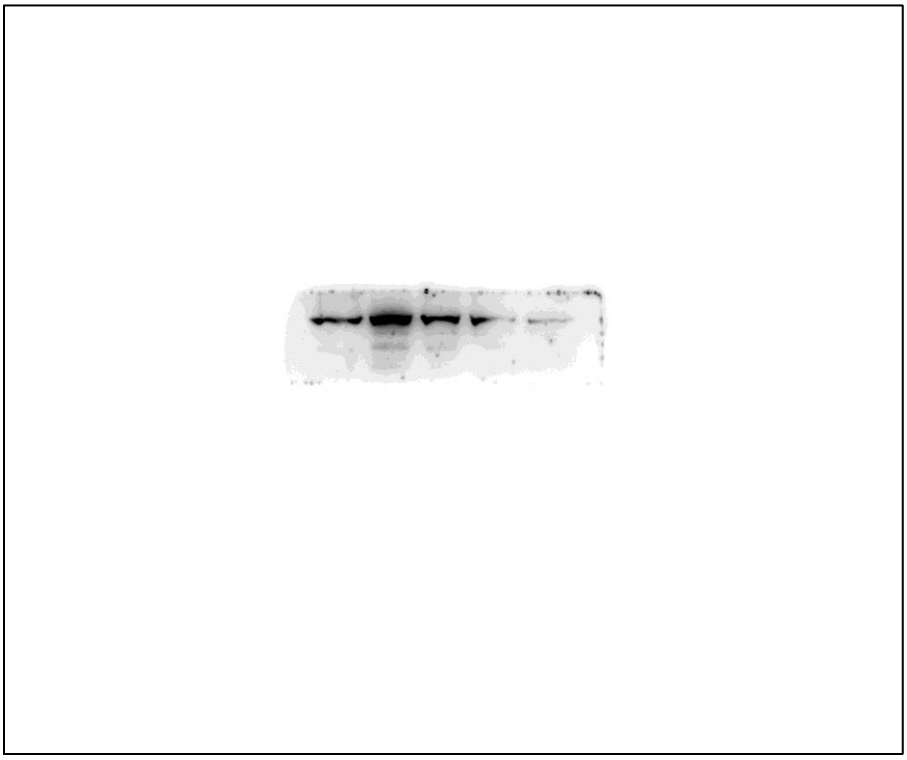

Fig 4A IL-1β

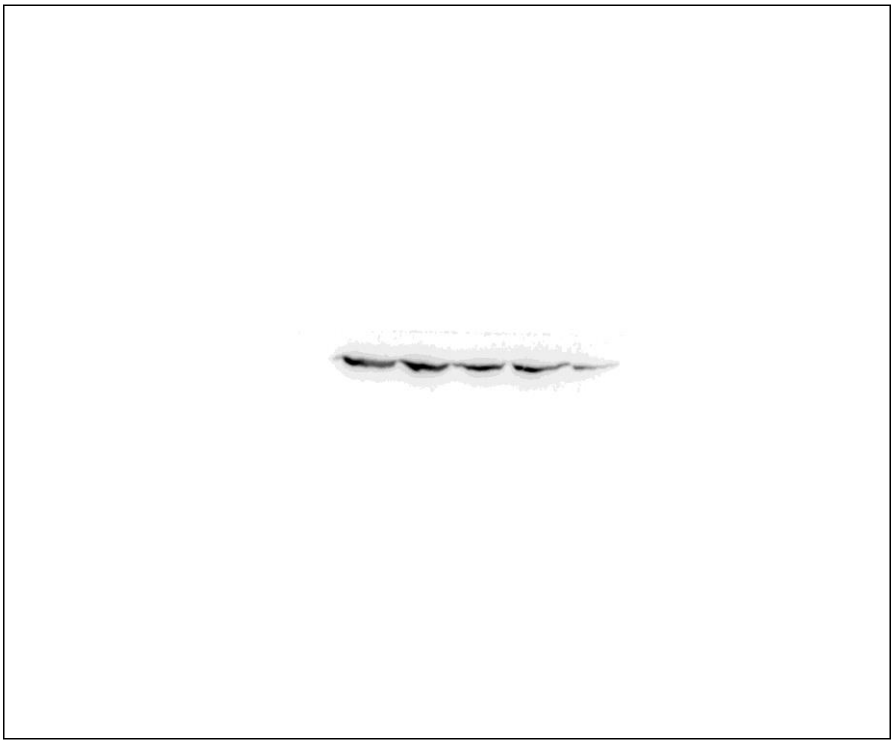

Fig 4A p-p65

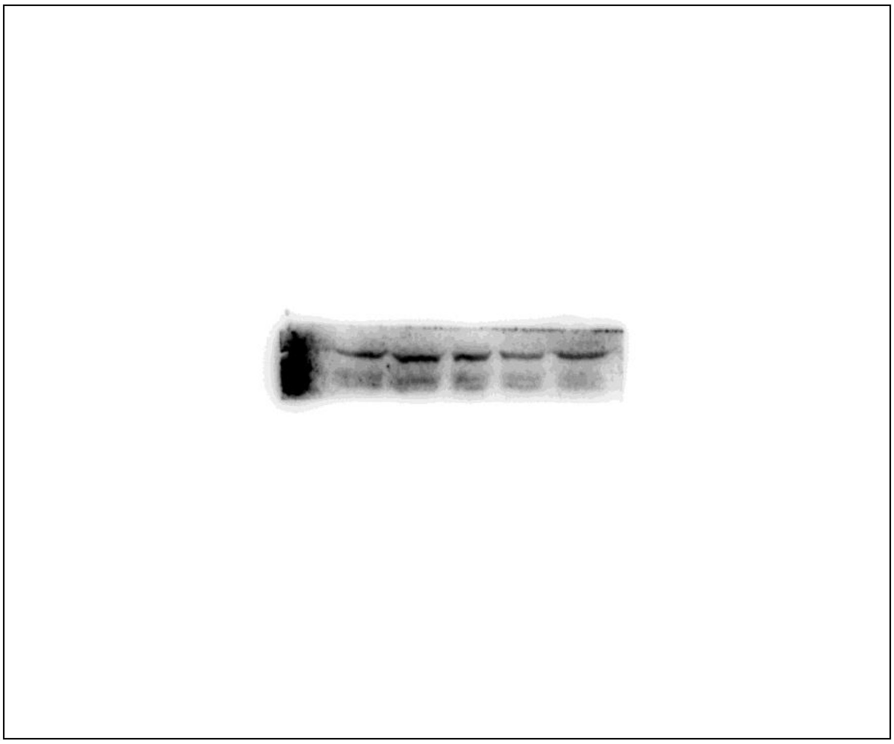

Fig 4A p65

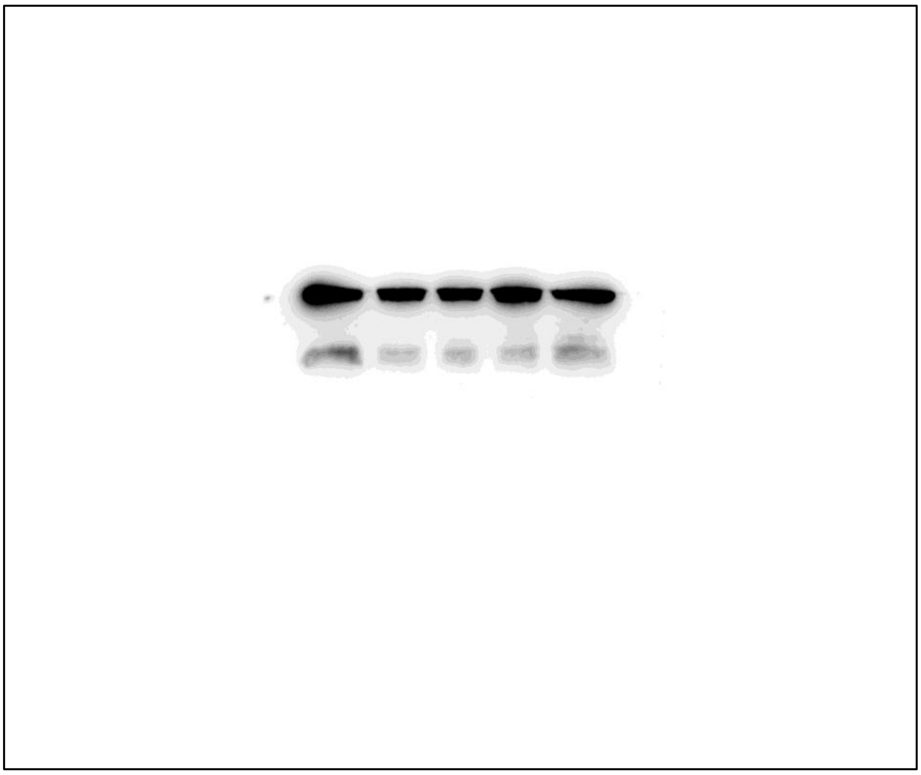

Fig 4A p-IκB

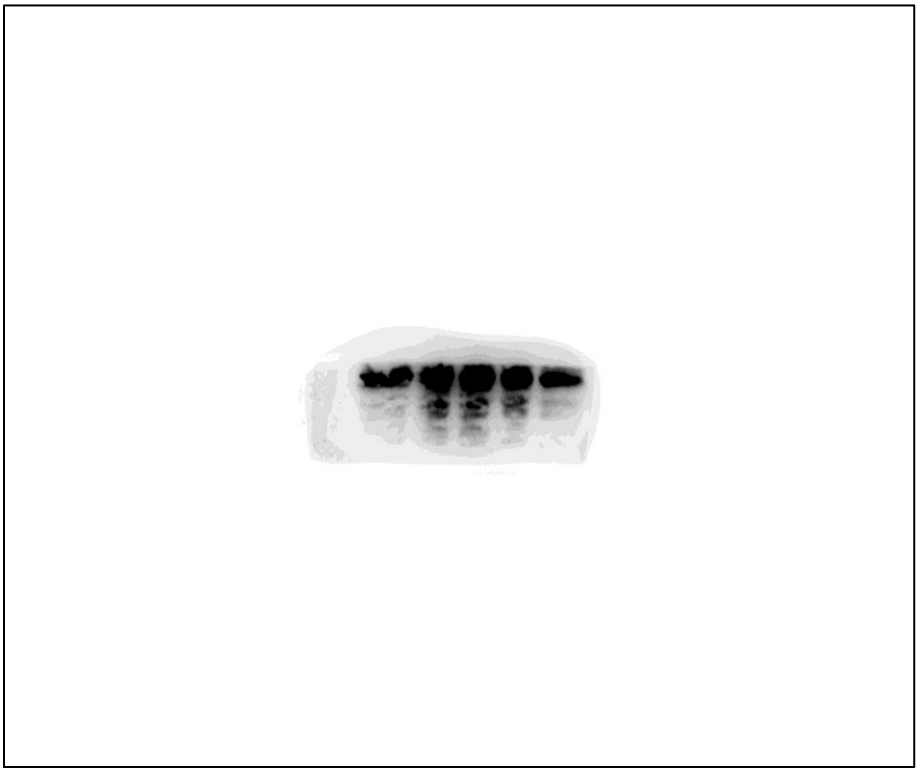

Fig 4A IκB

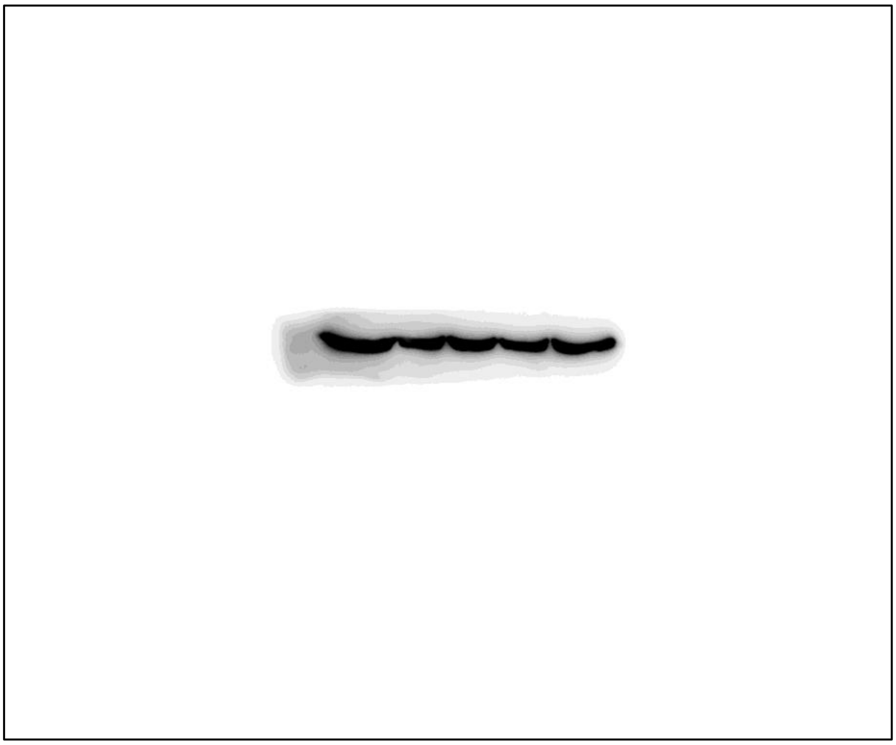

Fig 4A actin

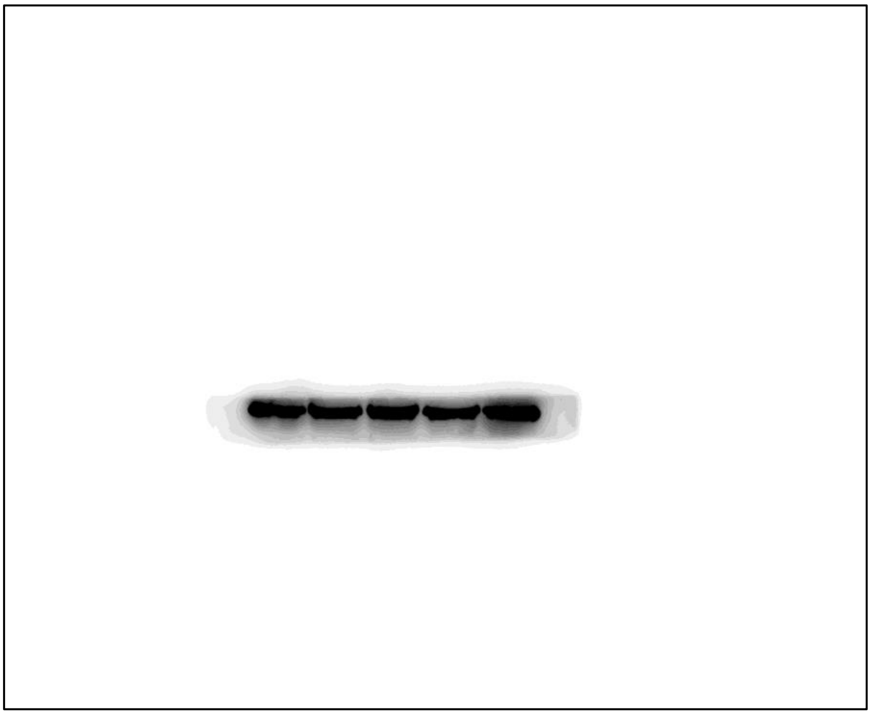

Fig 4H NLRP3

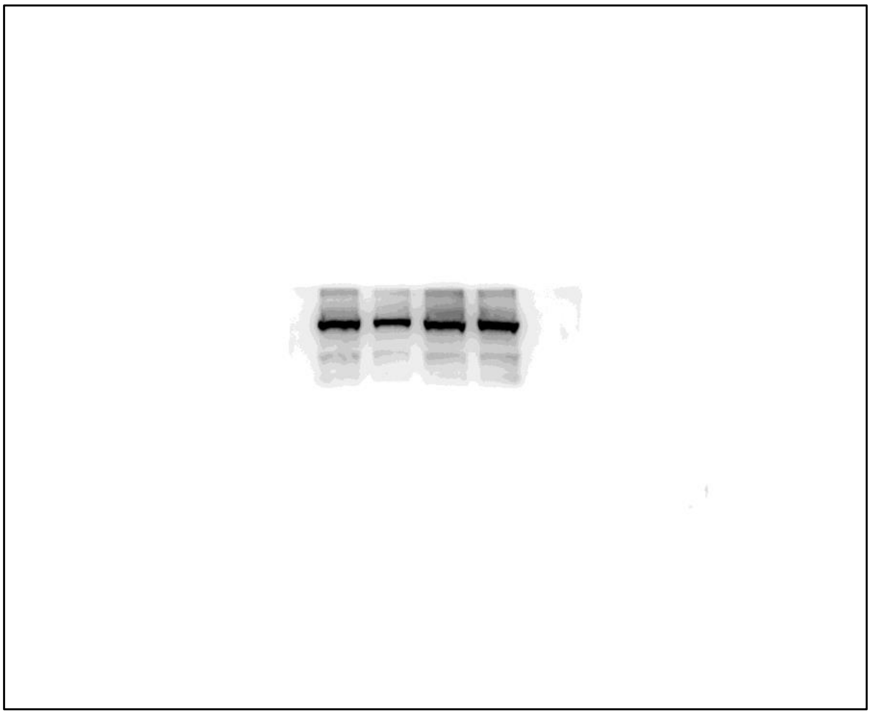

Fig 4H ASC

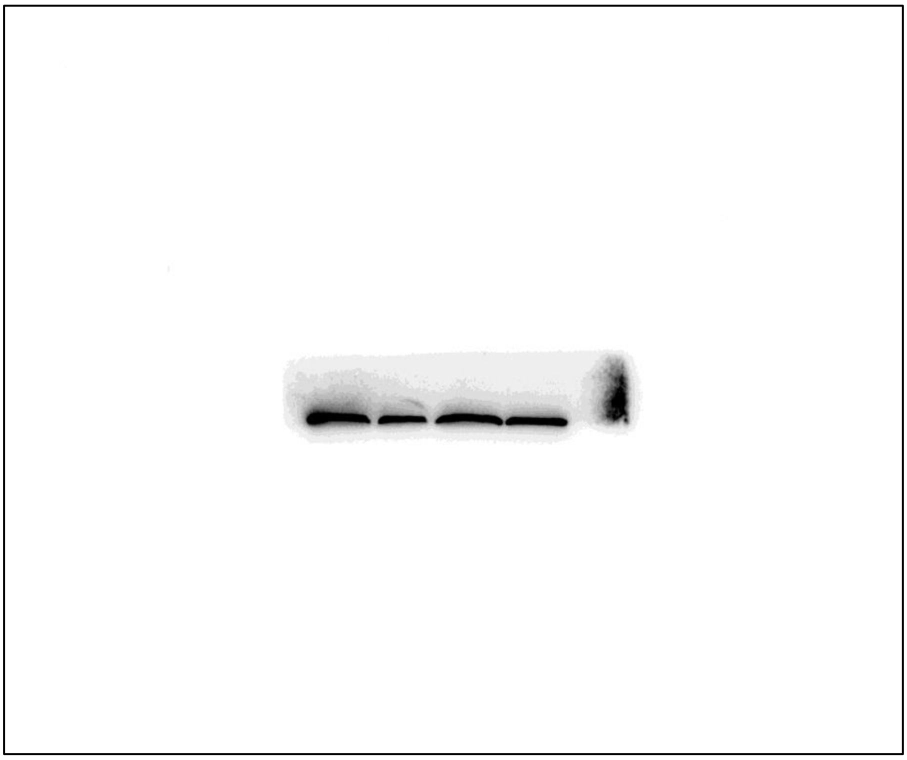

Fig 4H Caspase-1

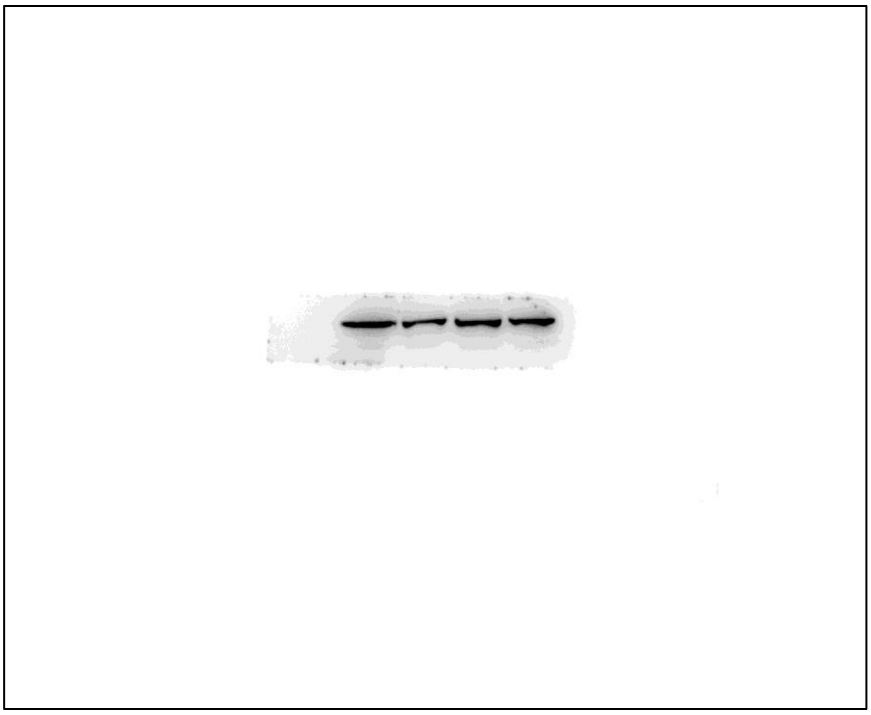

Fig 4H IL-1β

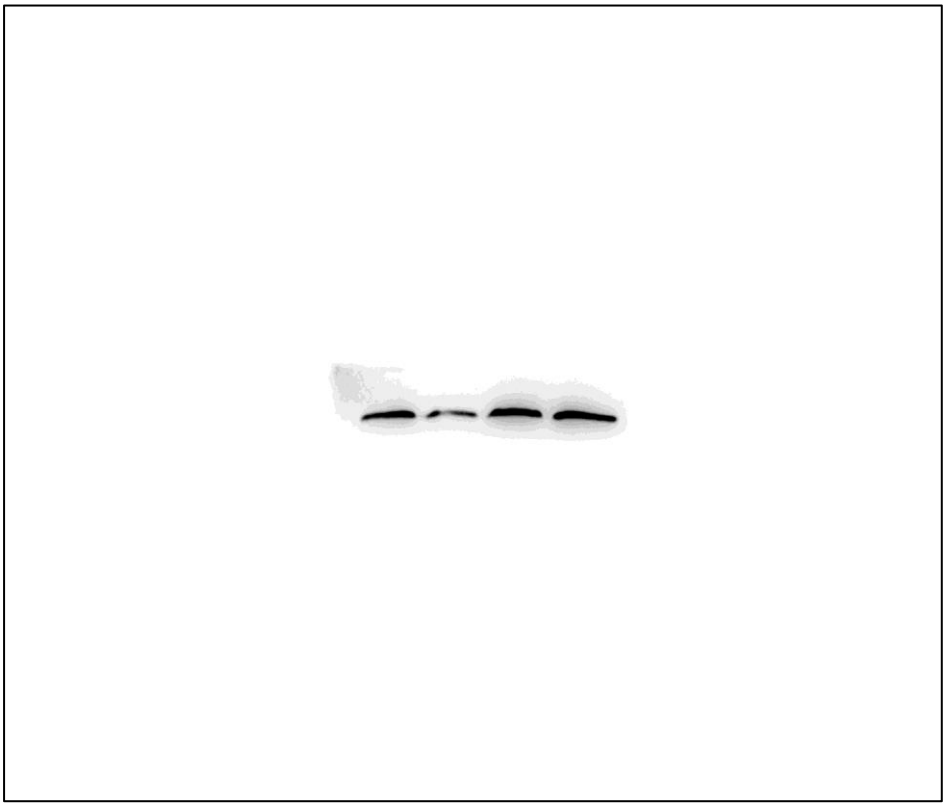

Fig 4H p-p65

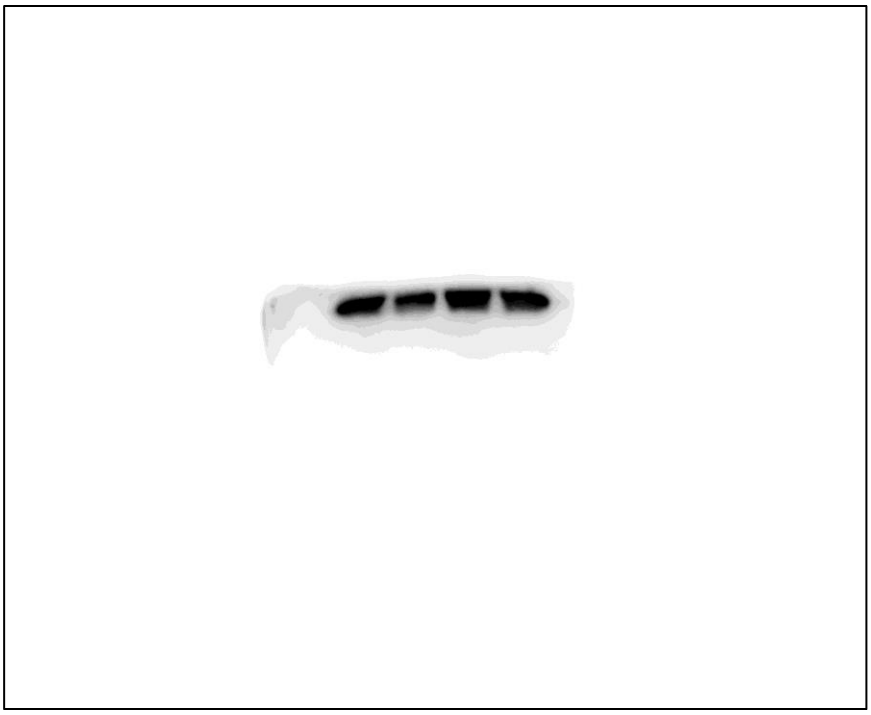

Fig 4H p65

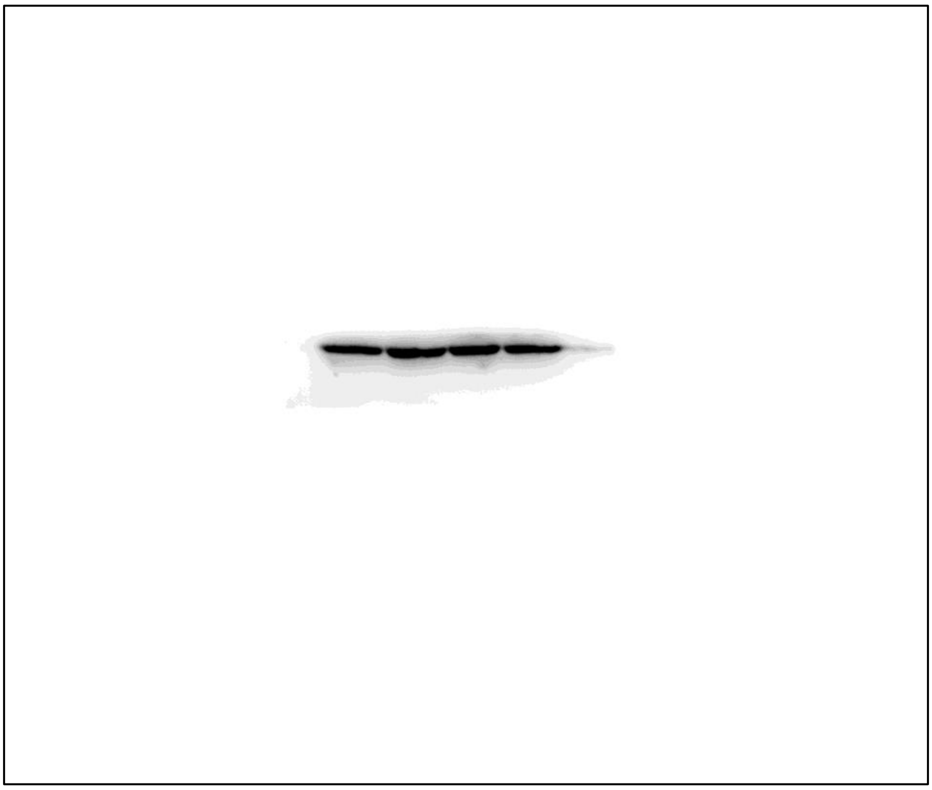

Fig 4H p-IκB

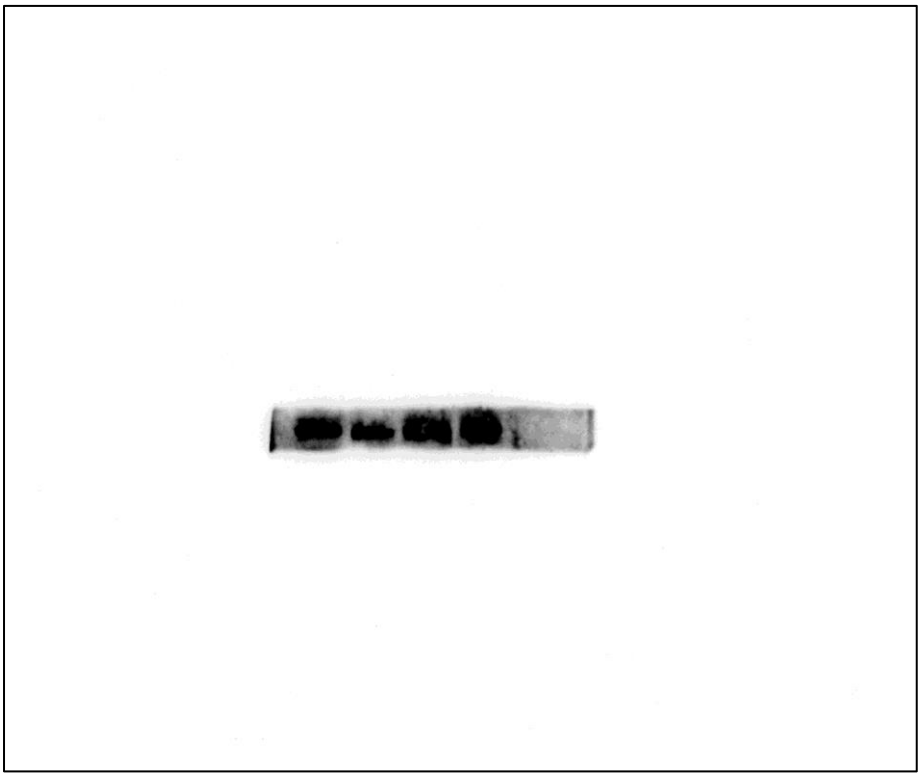

Fig 4H IκB

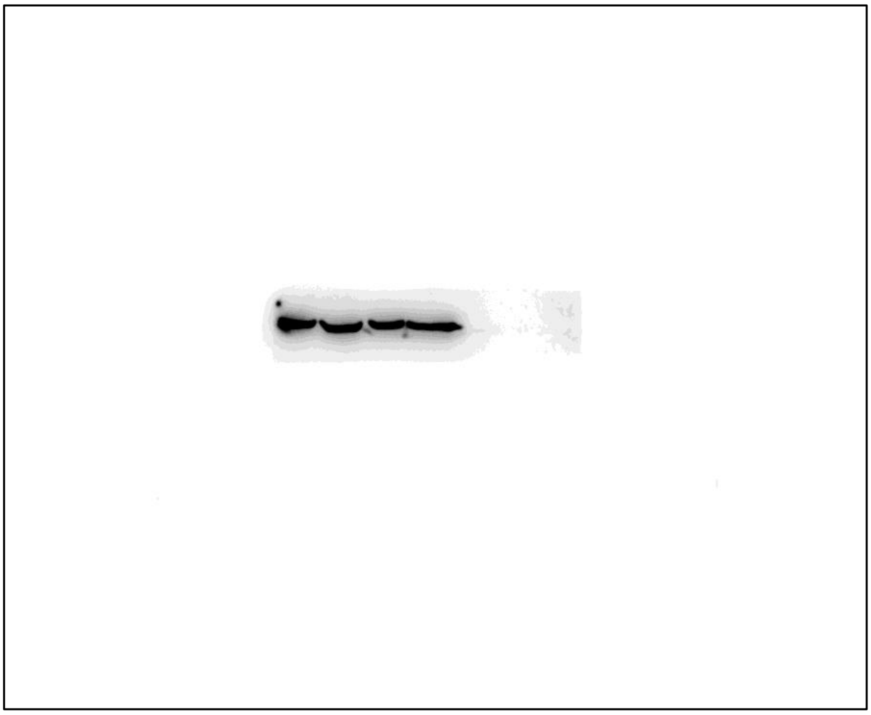

Fig 4H actin

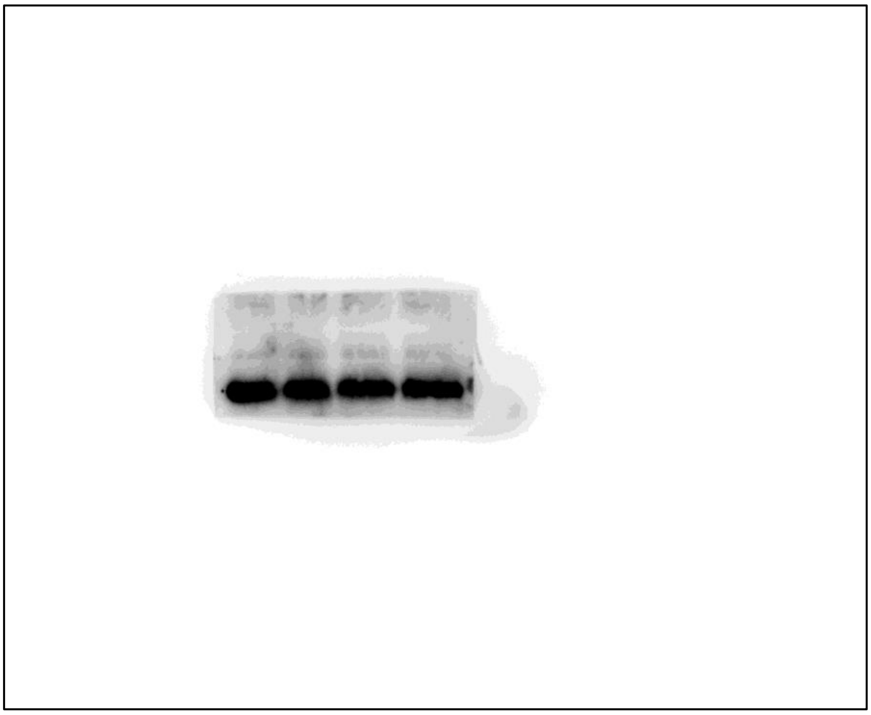

Fig 4O NLRP3

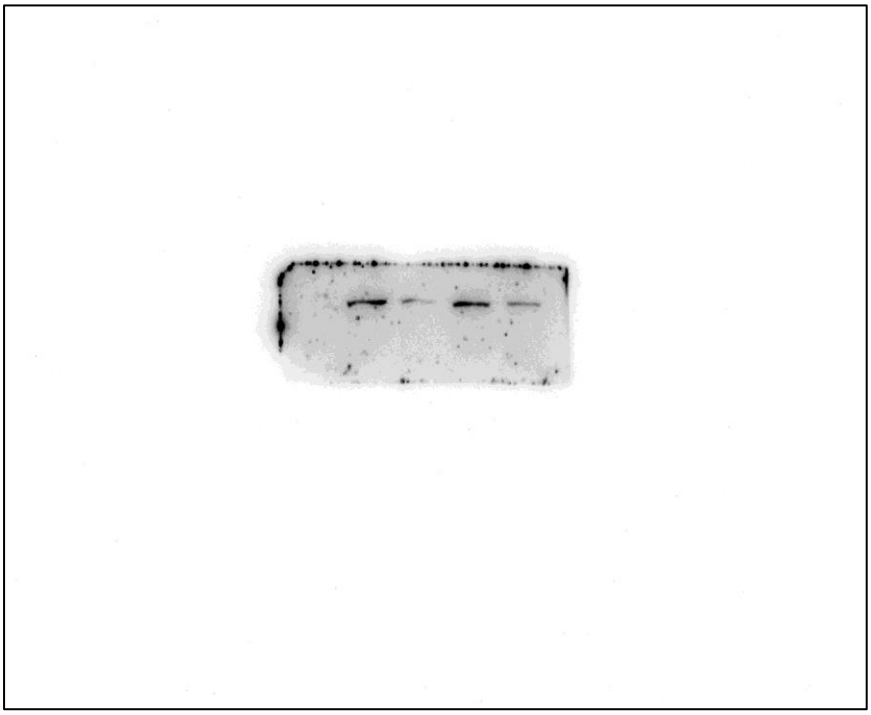

Fig 4O ASC

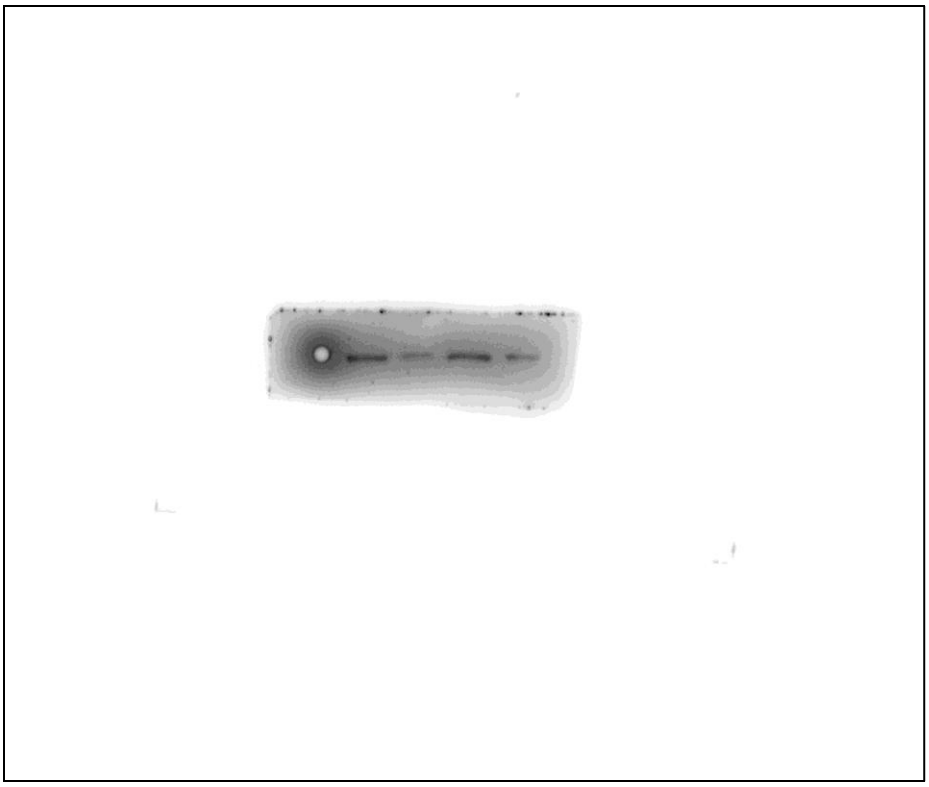

Fig 4O Caspase-1

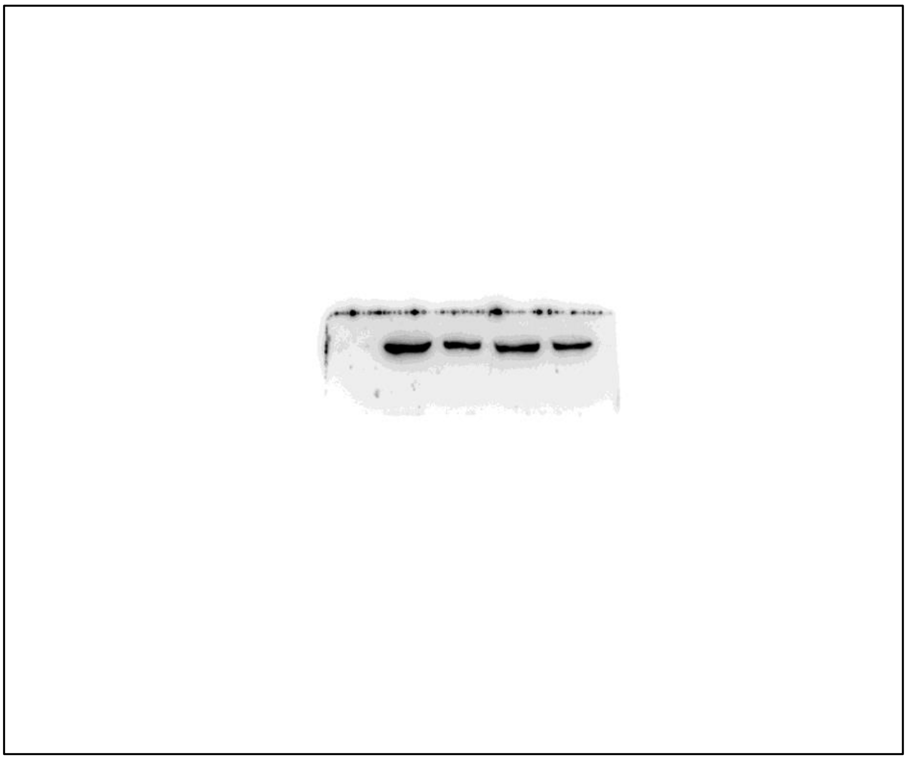

Fig 4O IL-1β

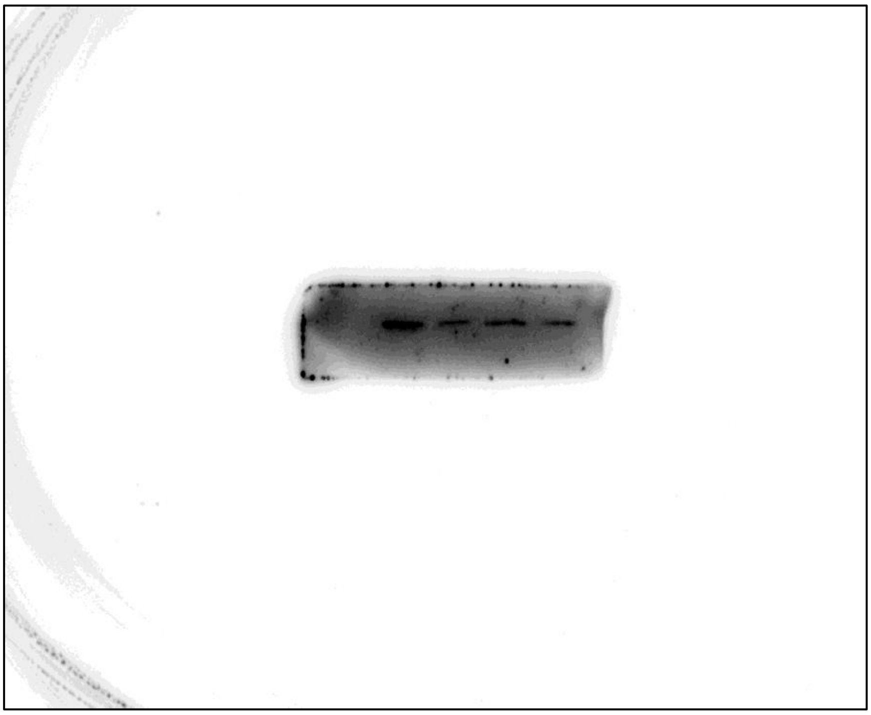

Fig 4O p-p65

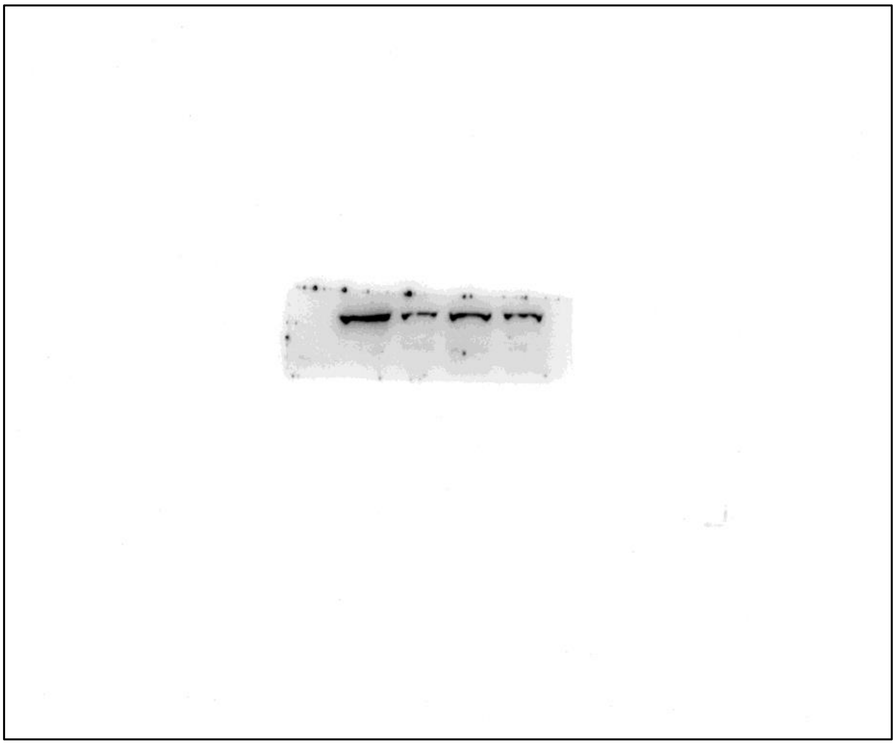

Fig 4O p65

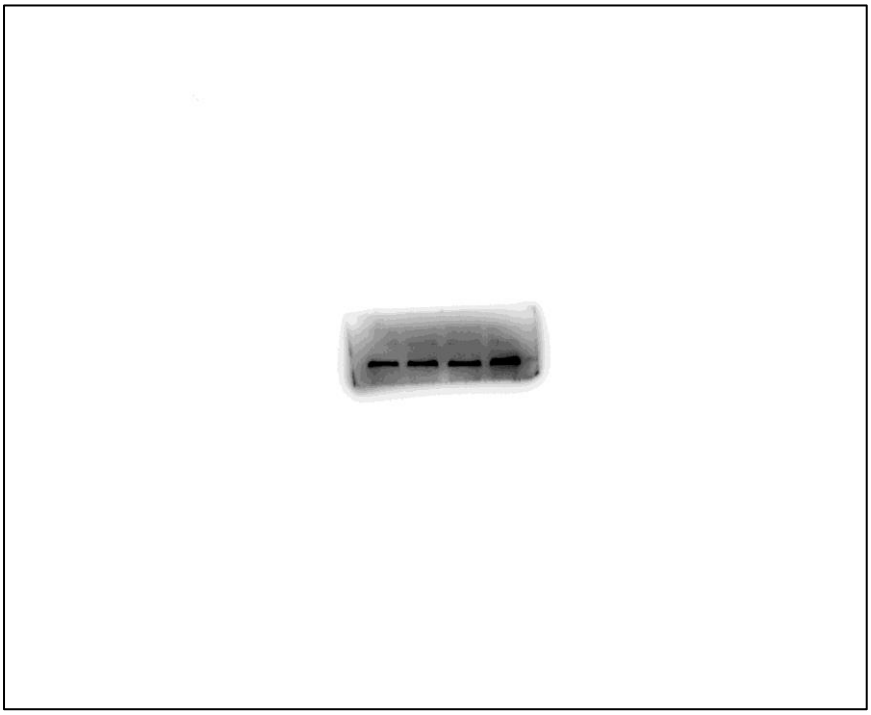

Fig 4O p-IκB

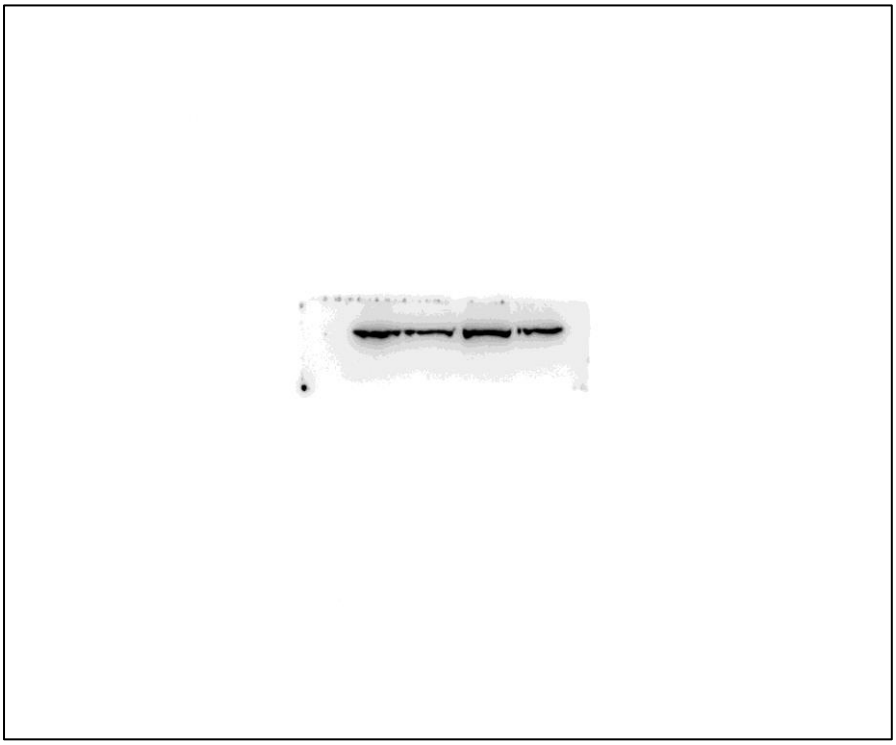

Fig 4O IκB

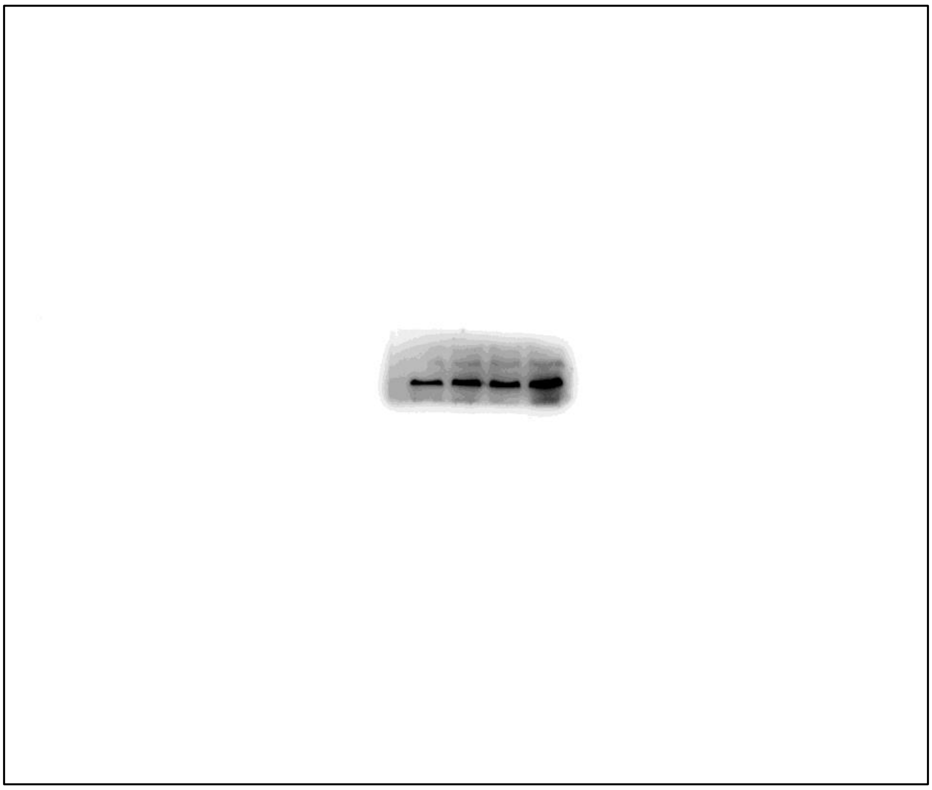

Fig 4O actin

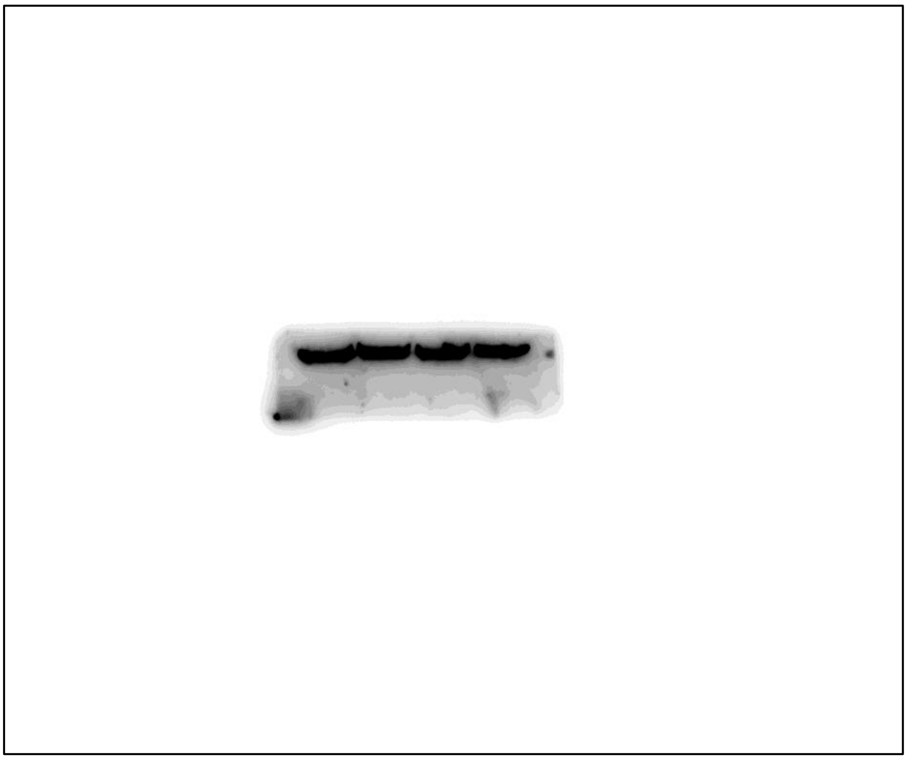

Fig 5A NLRP3

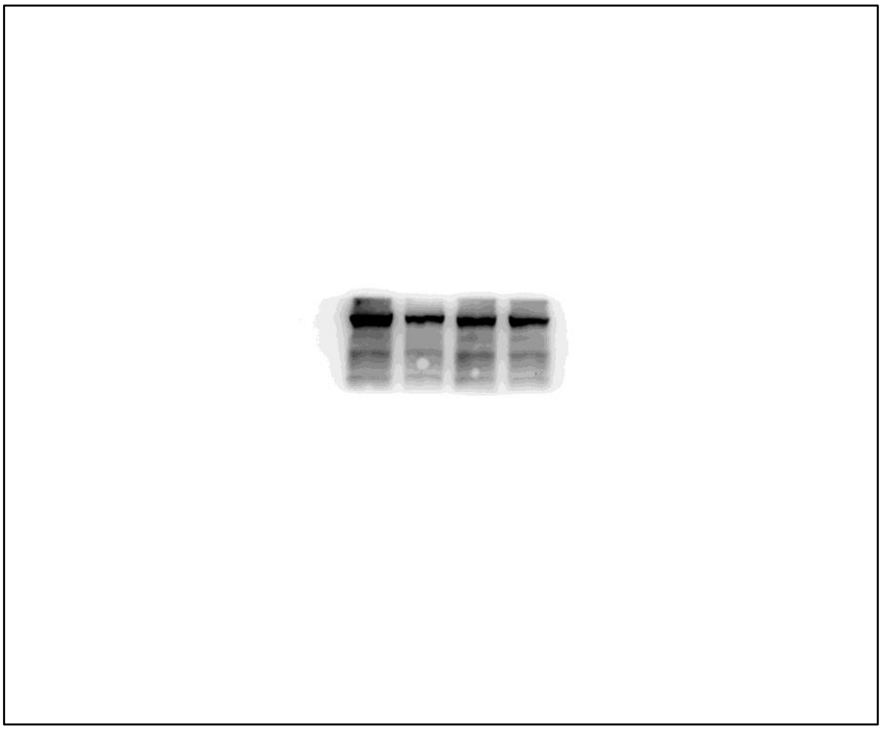

Fig 5A ASC

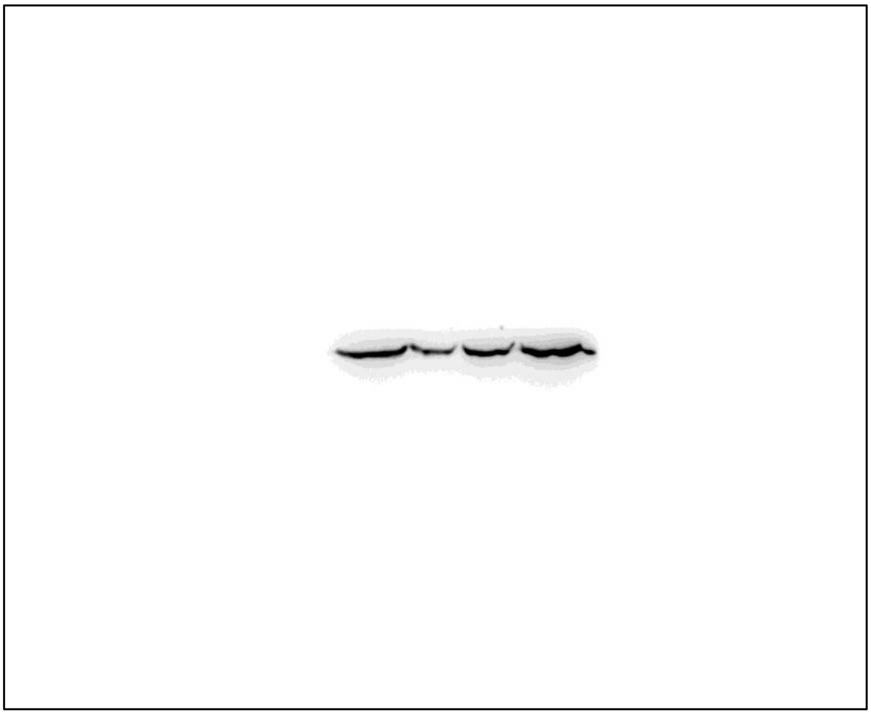

Fig 5A Caspase-1

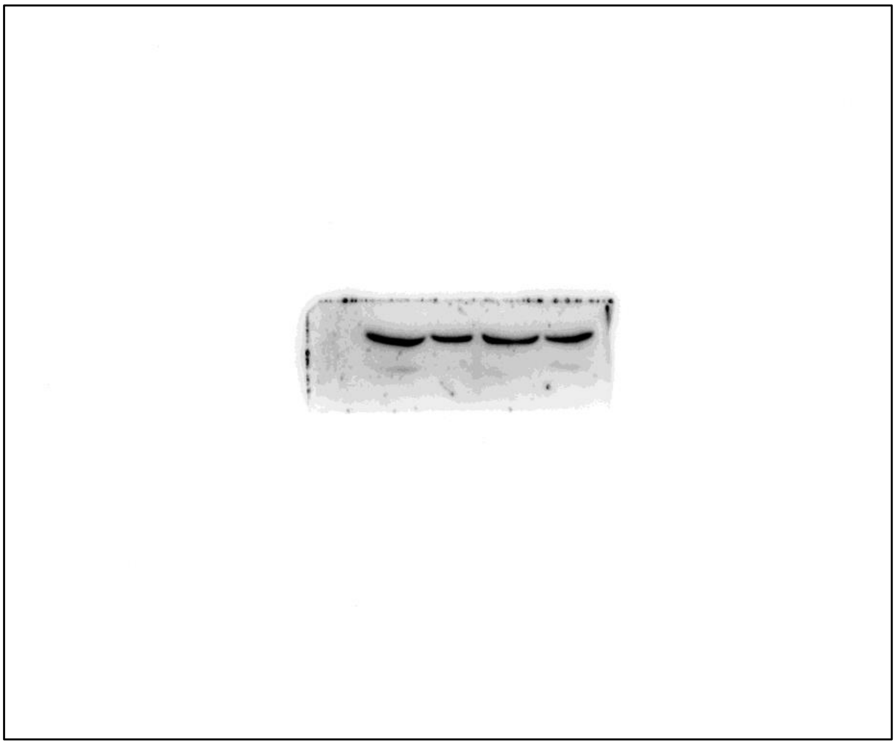

Fig 5A IL-1β

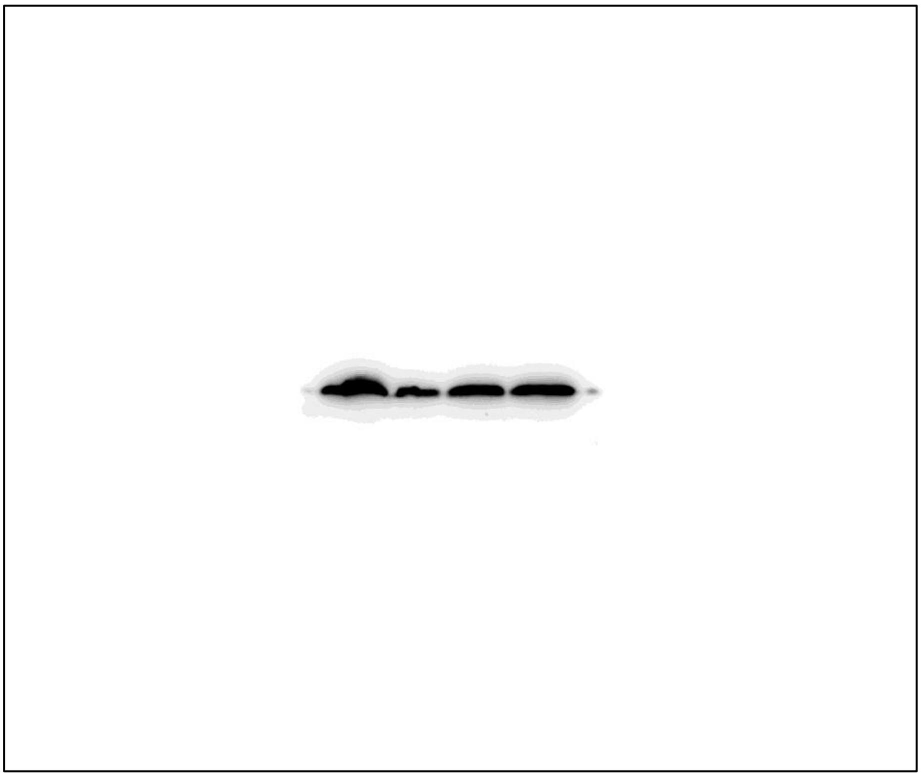

Fig 5A p-p65

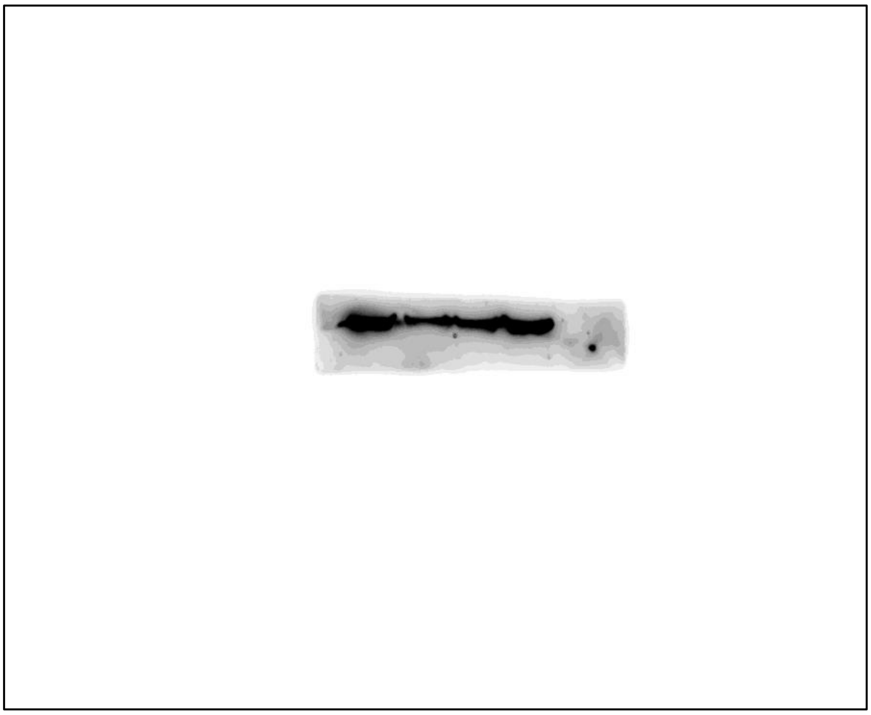

Fig 5A p65

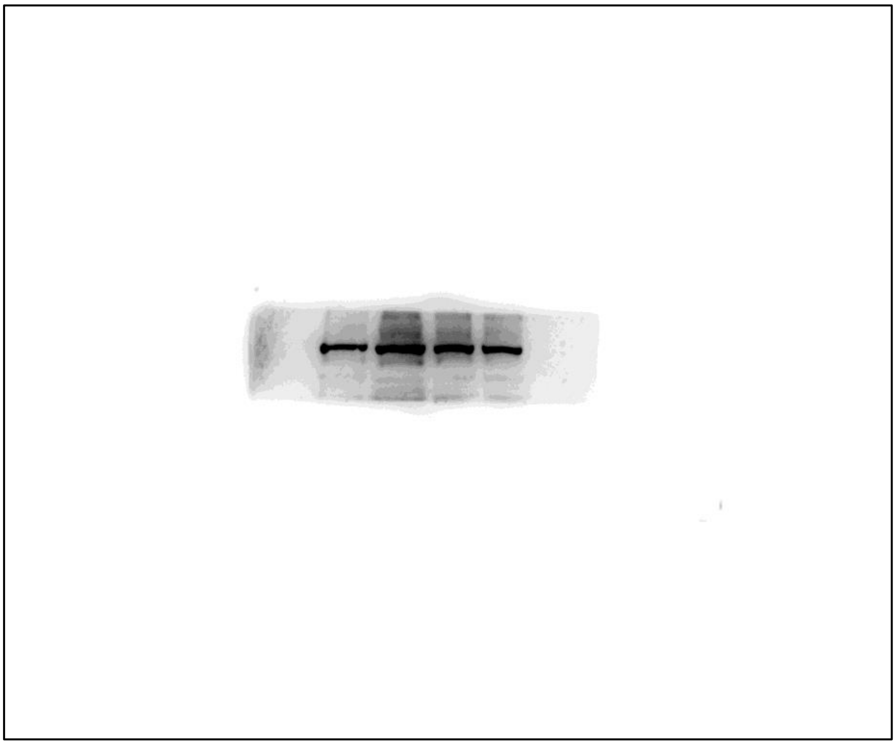

Fig 5A p-IκB

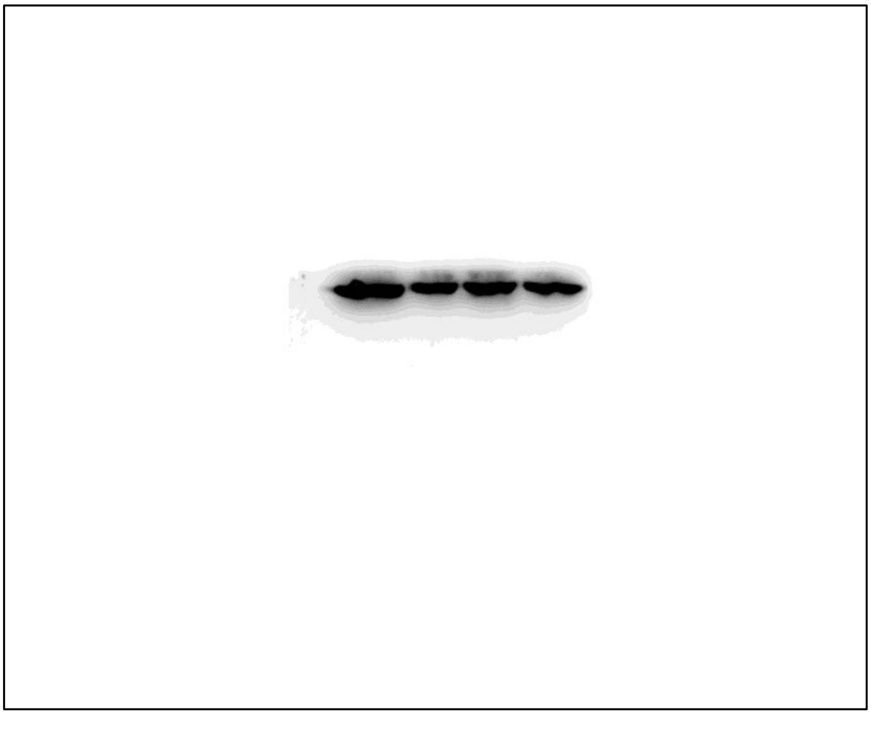

Fig 5A IκB

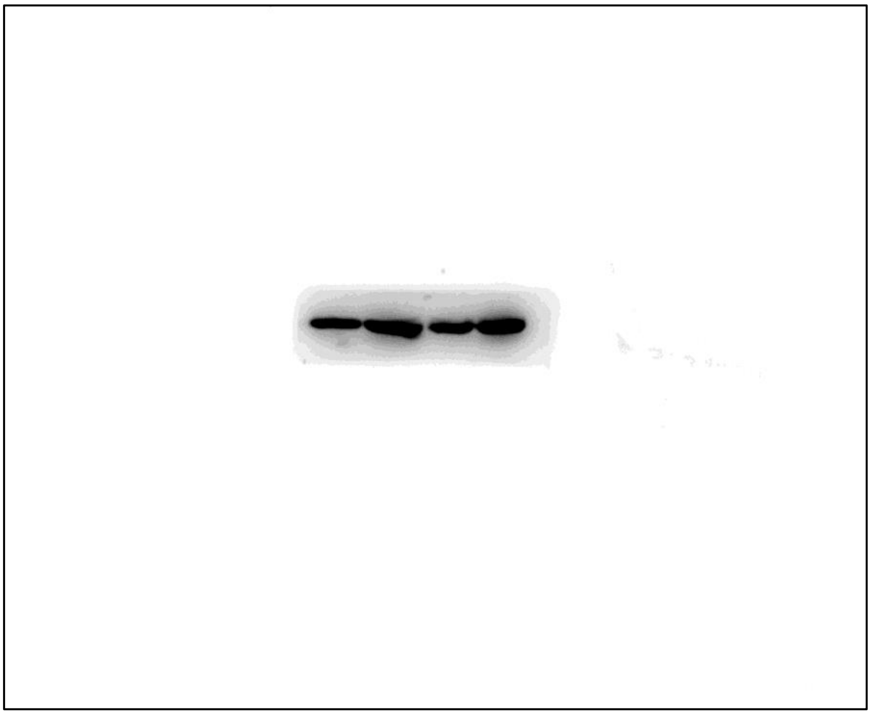

Fig 5A actin

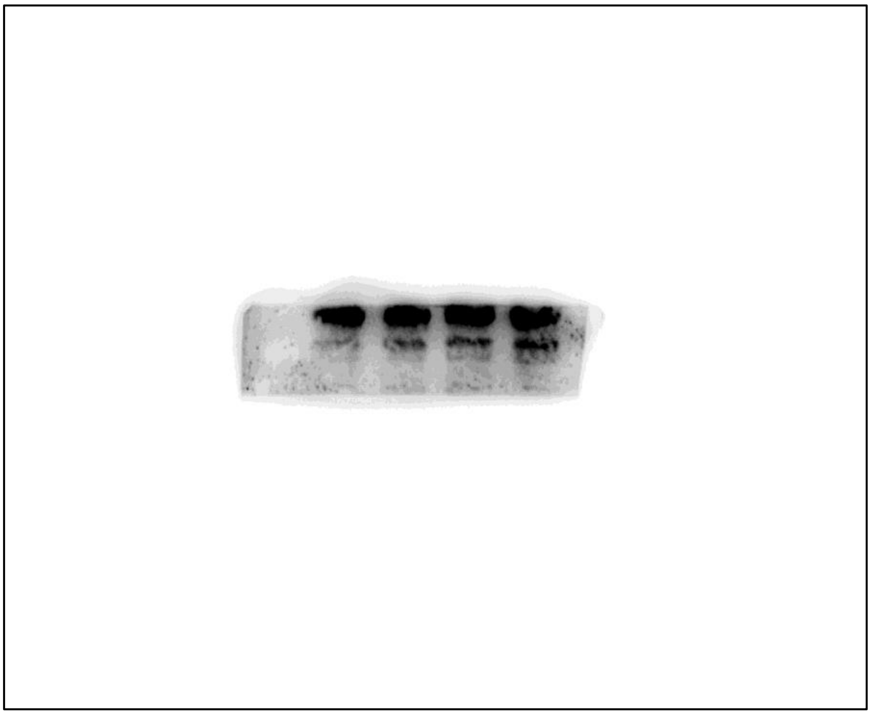

Fig 5H NLRP3

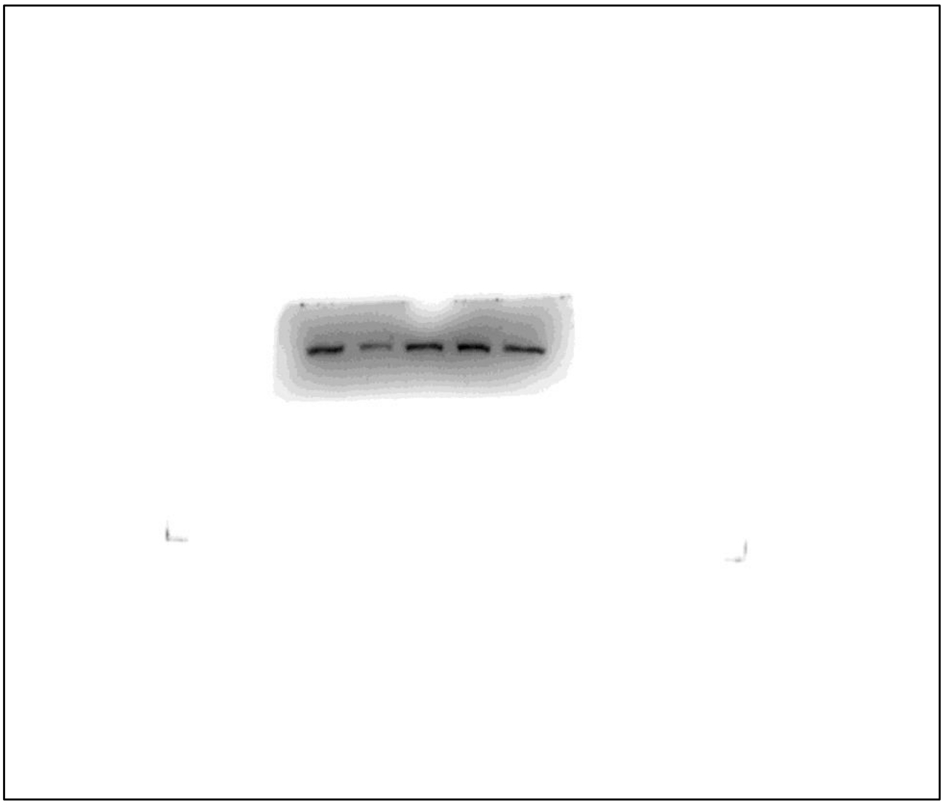

Fig 5H ASC

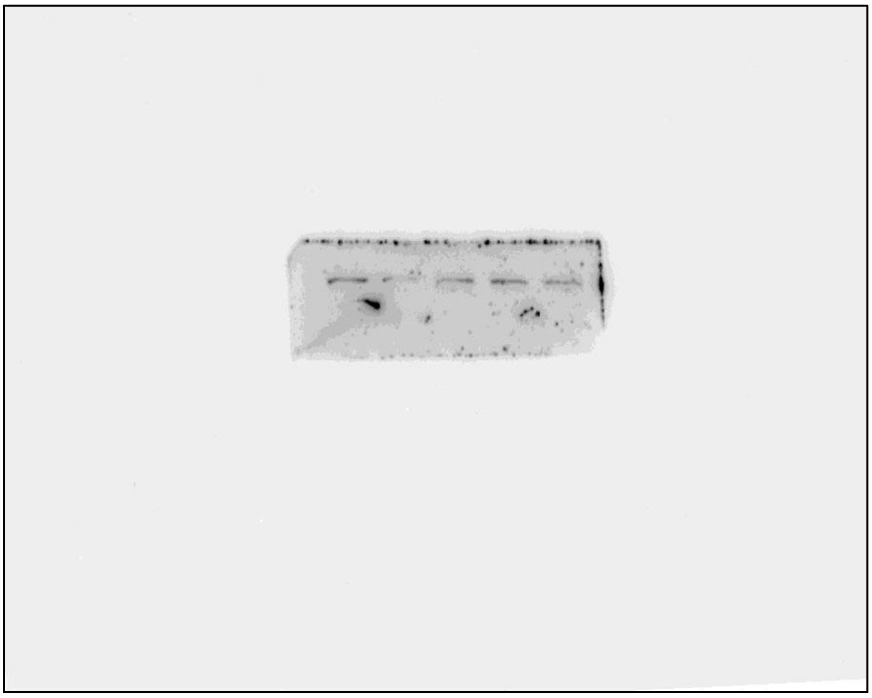

Fig 5H Caspase-1

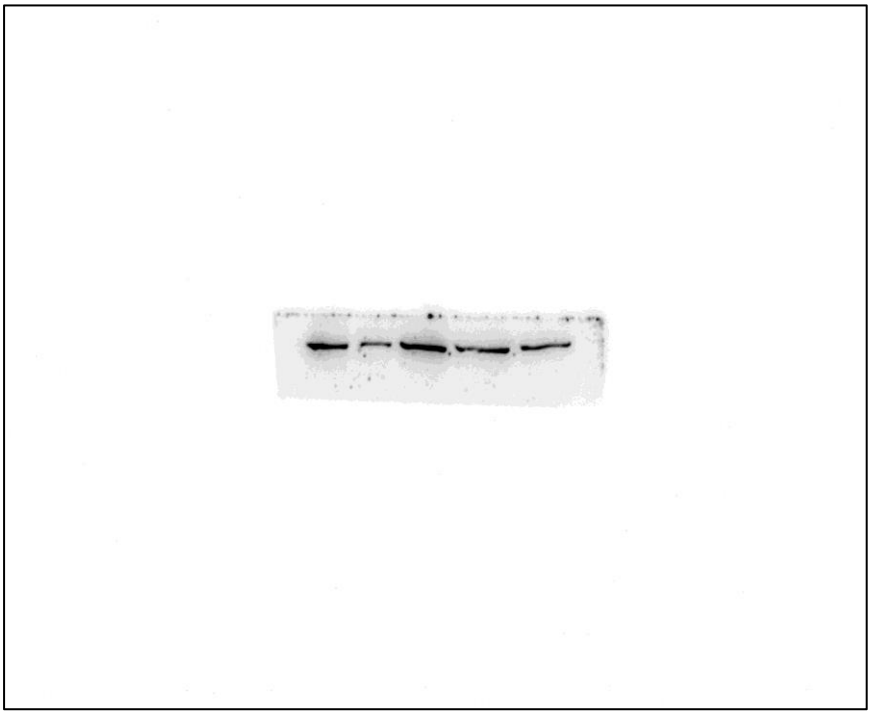

Fig 5H IL-1β

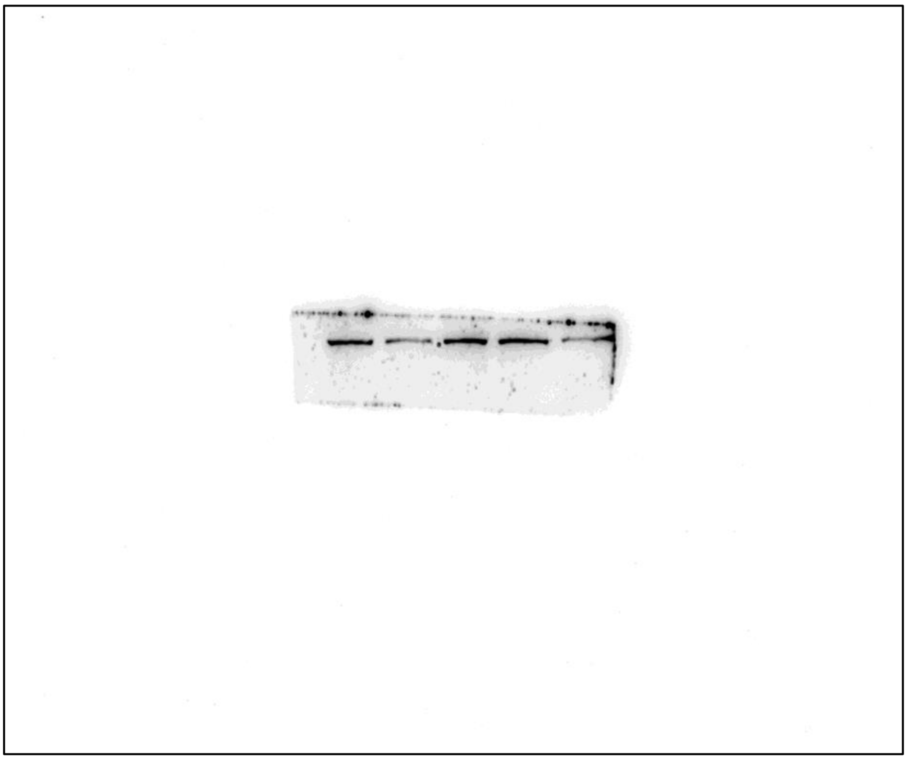

Fig 5H p-p65

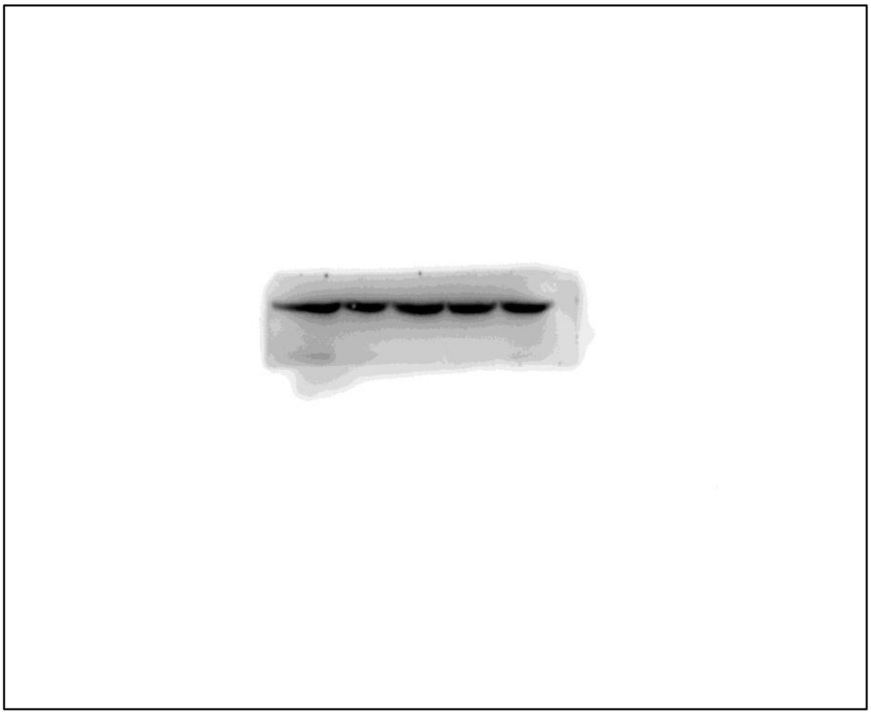

Fig 5H p65

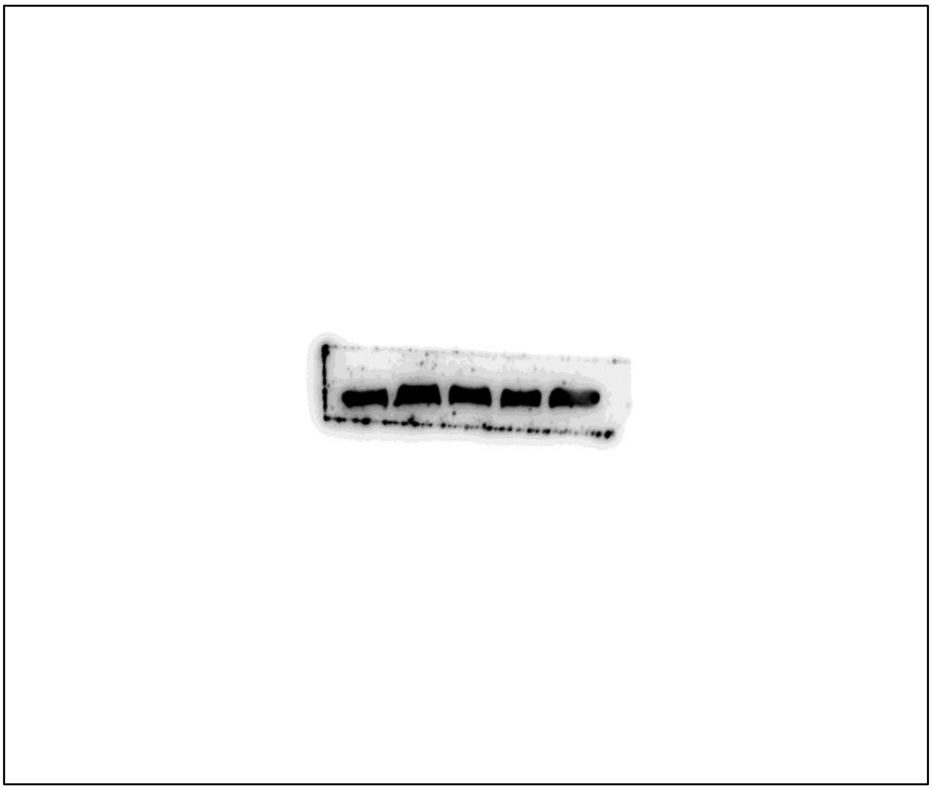

Fig 5H p-IκB

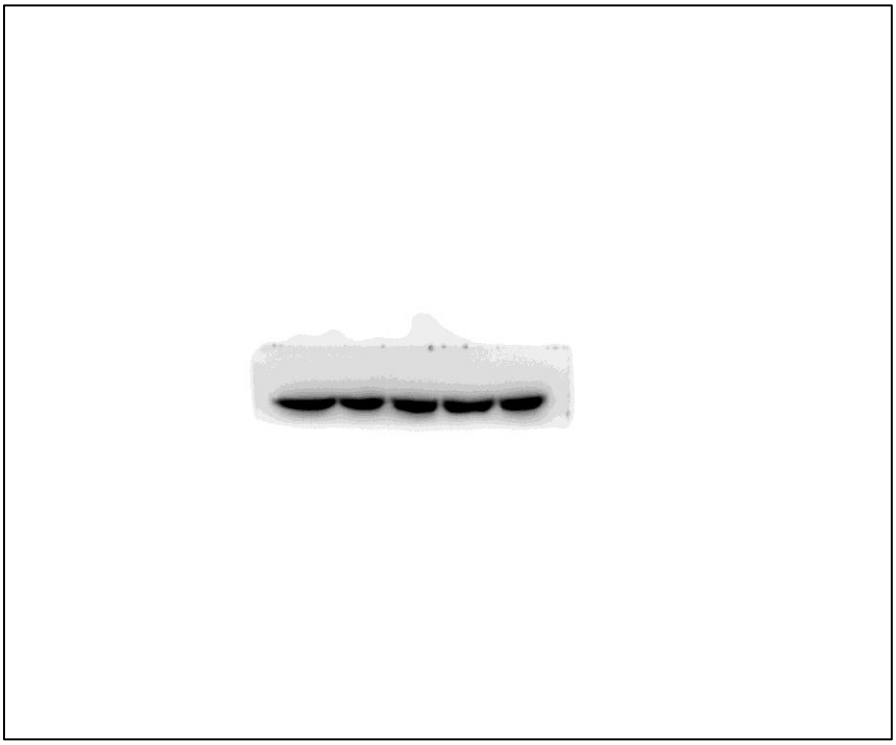

Fig 5H IκB

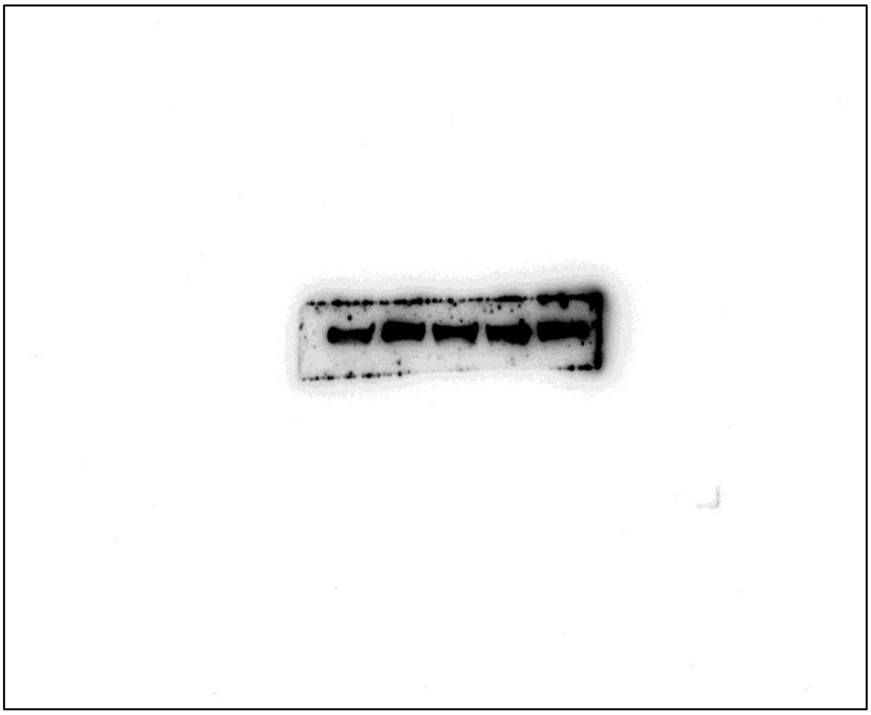

Fig 5H actin

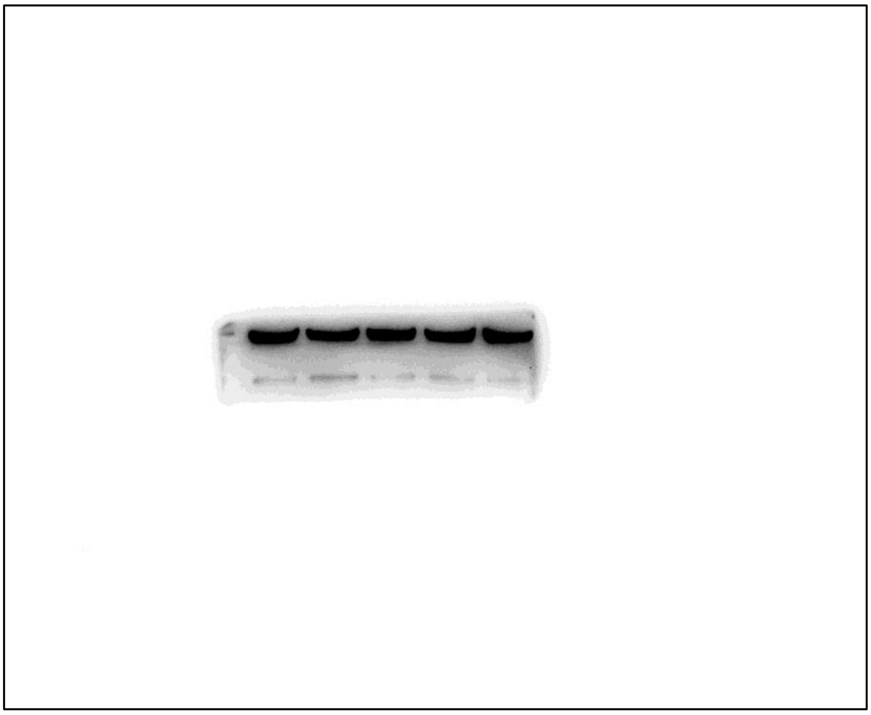

Fig 6M ZO-1

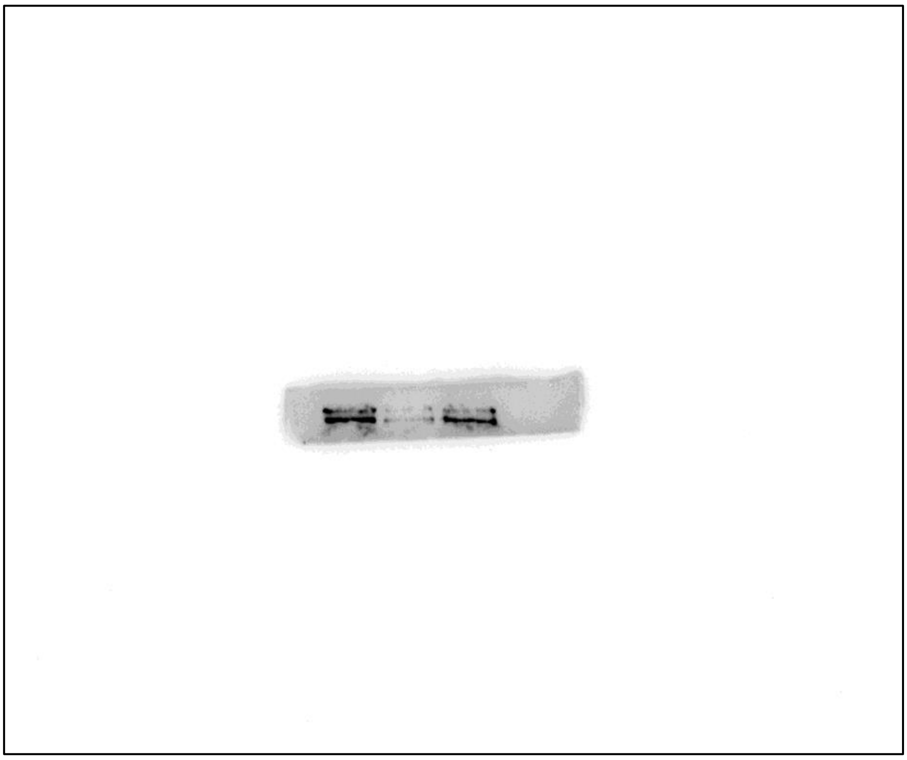

Fig 6M Occludin

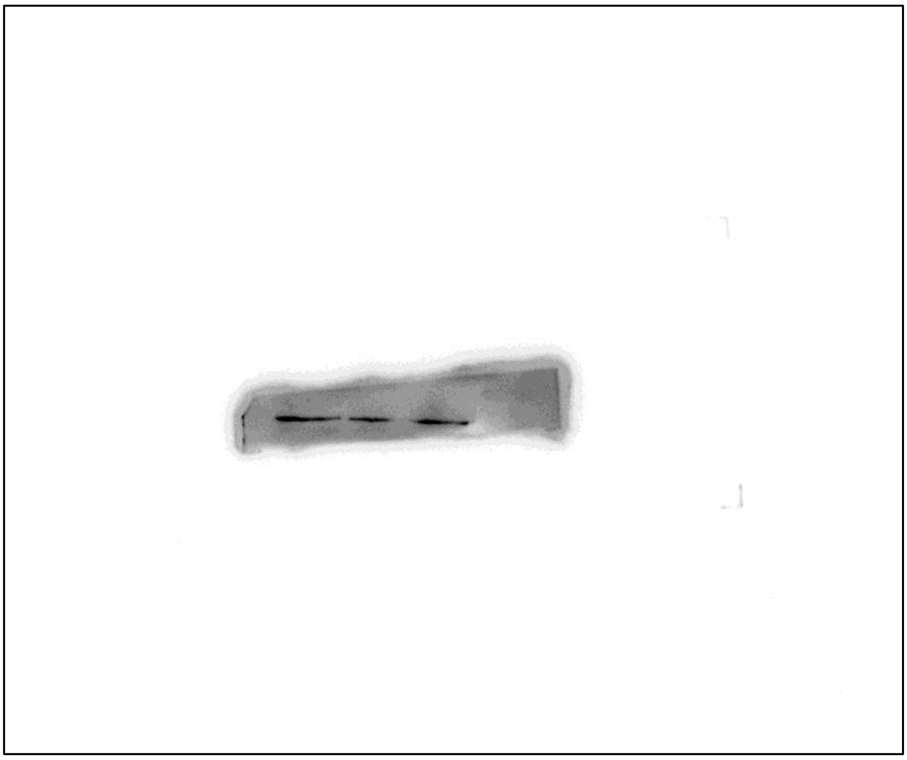

Fig 6M Claudin-3

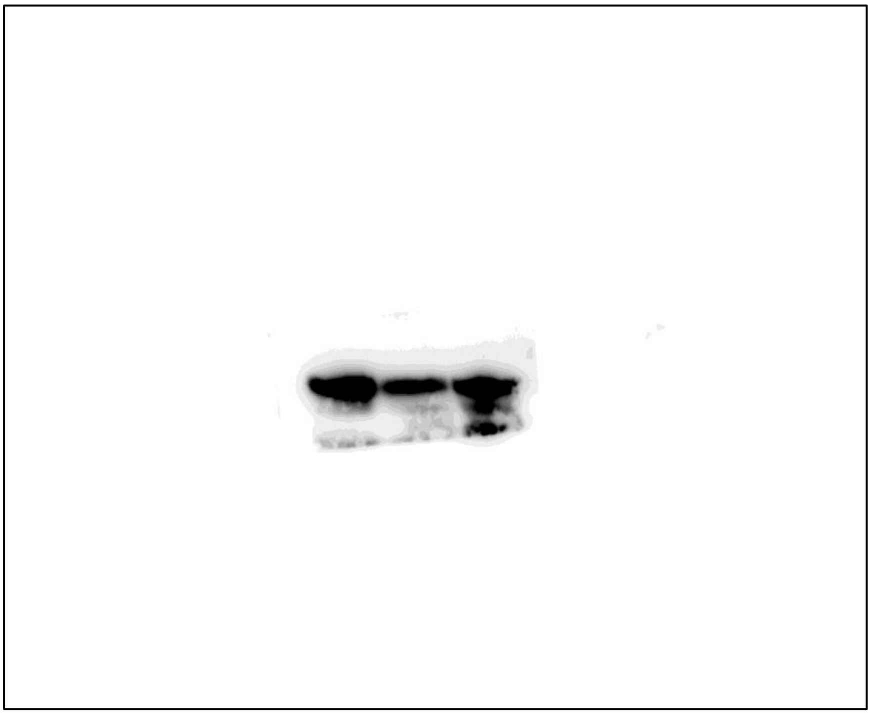

Fig 6M actin

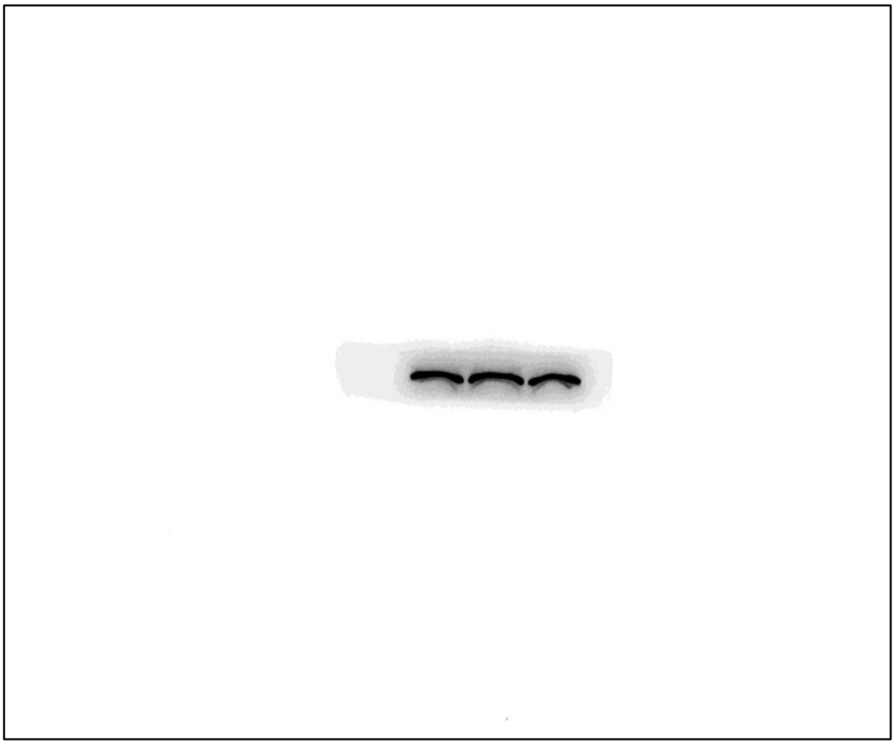

Fig 6Q p-p65

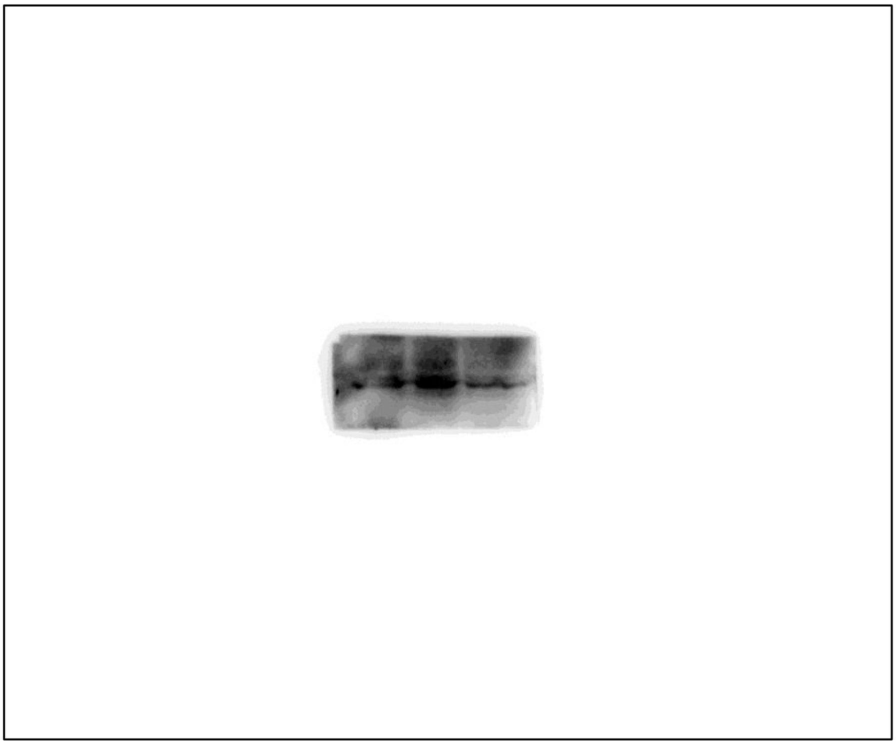

Fig 6Q p65

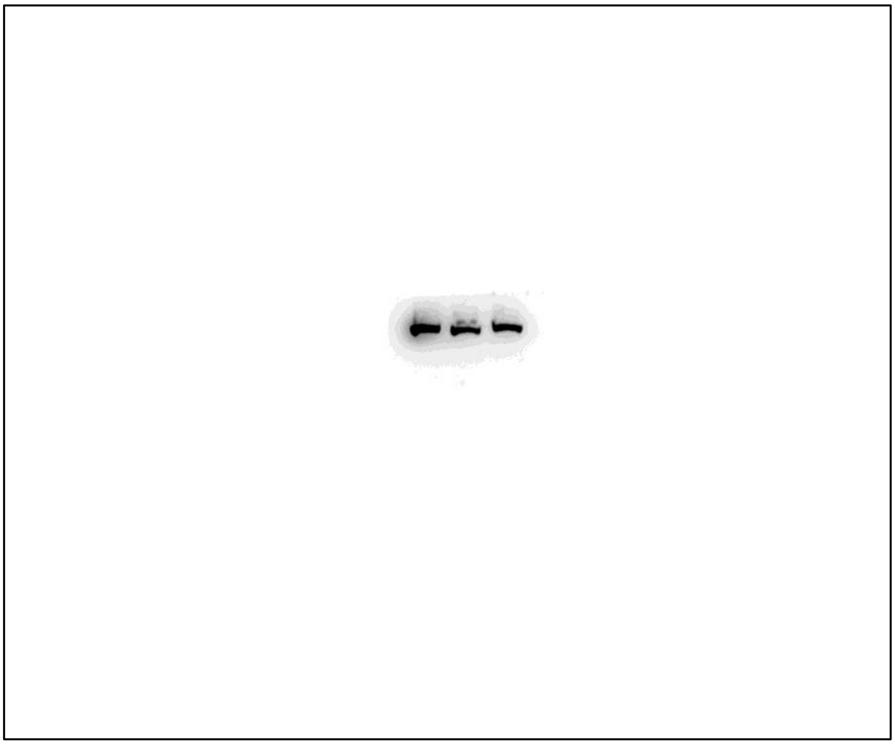

Fig 6Q NLRP3

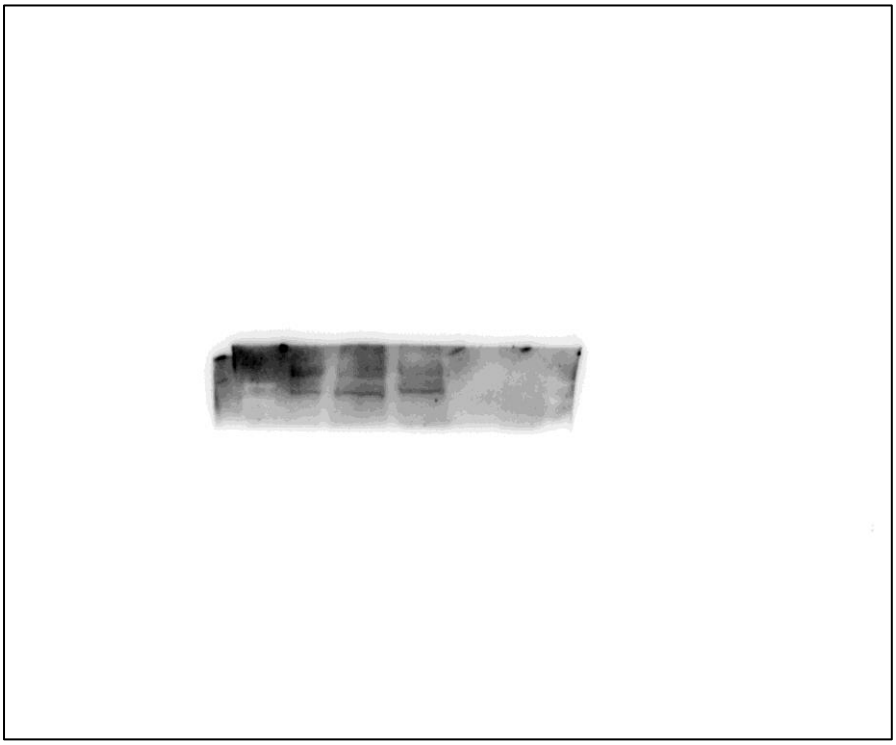

Fig 6Q ASC

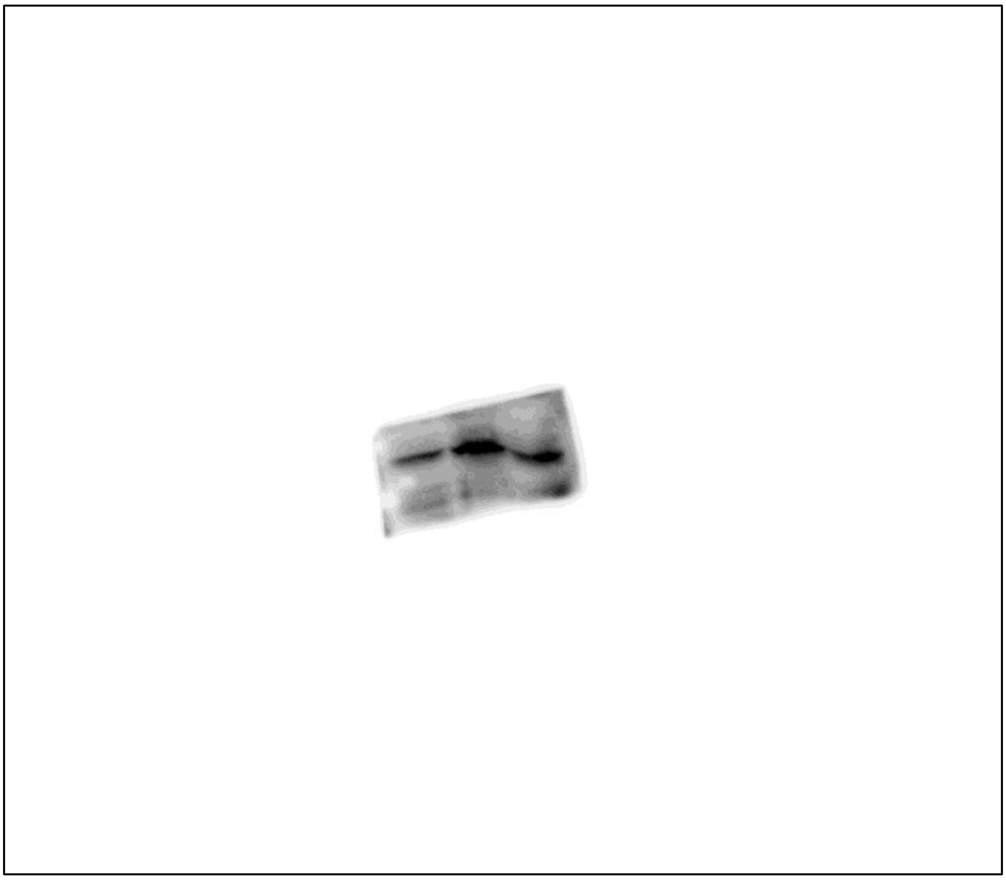

Fig 6Q IL-1β

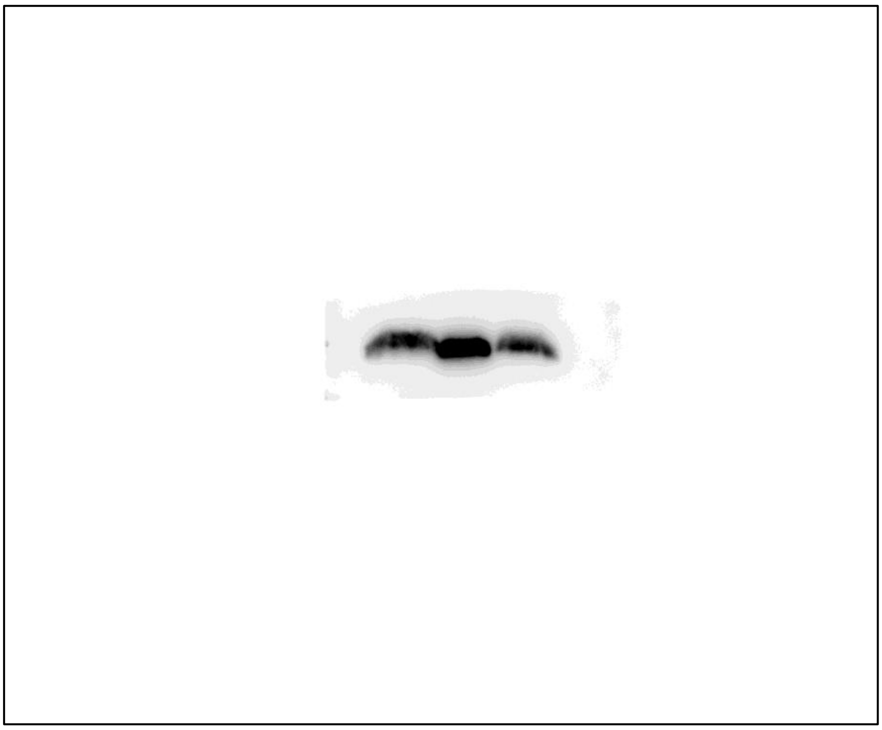

Fig 6Q actin

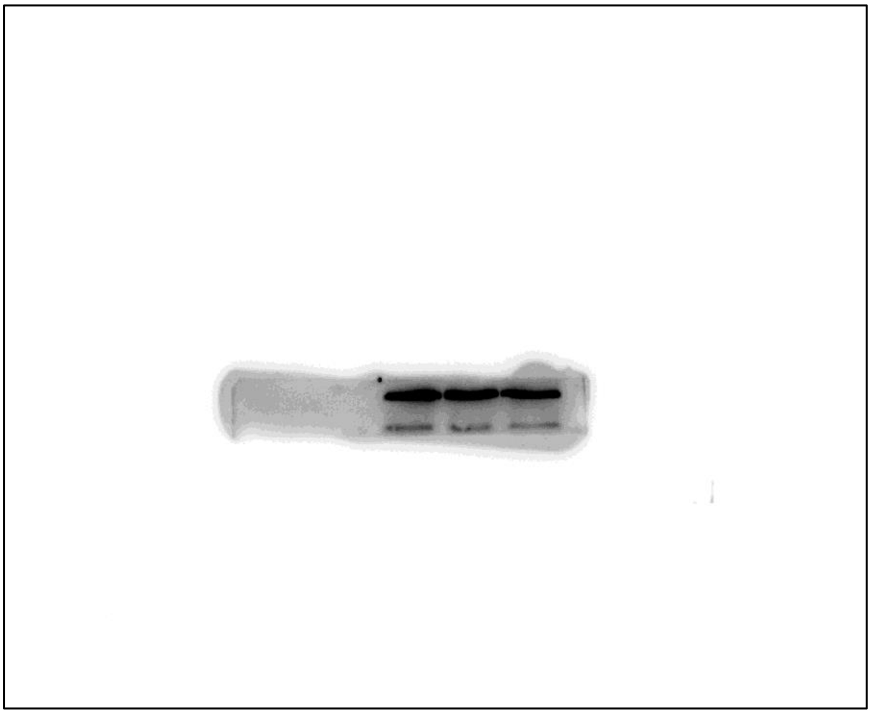

Fig 7I ZO-1

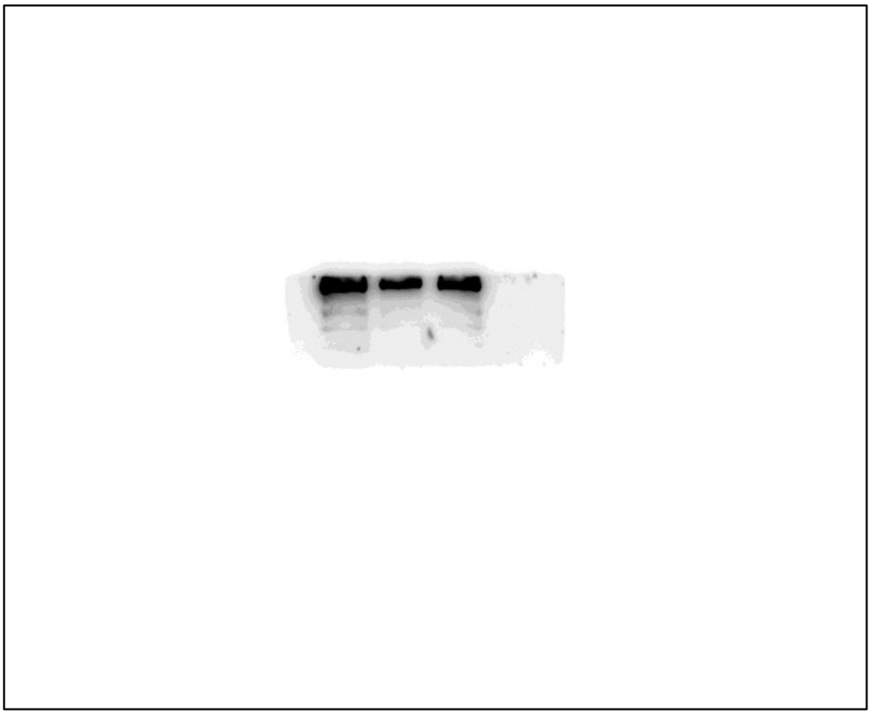

Fig 7I Occludin

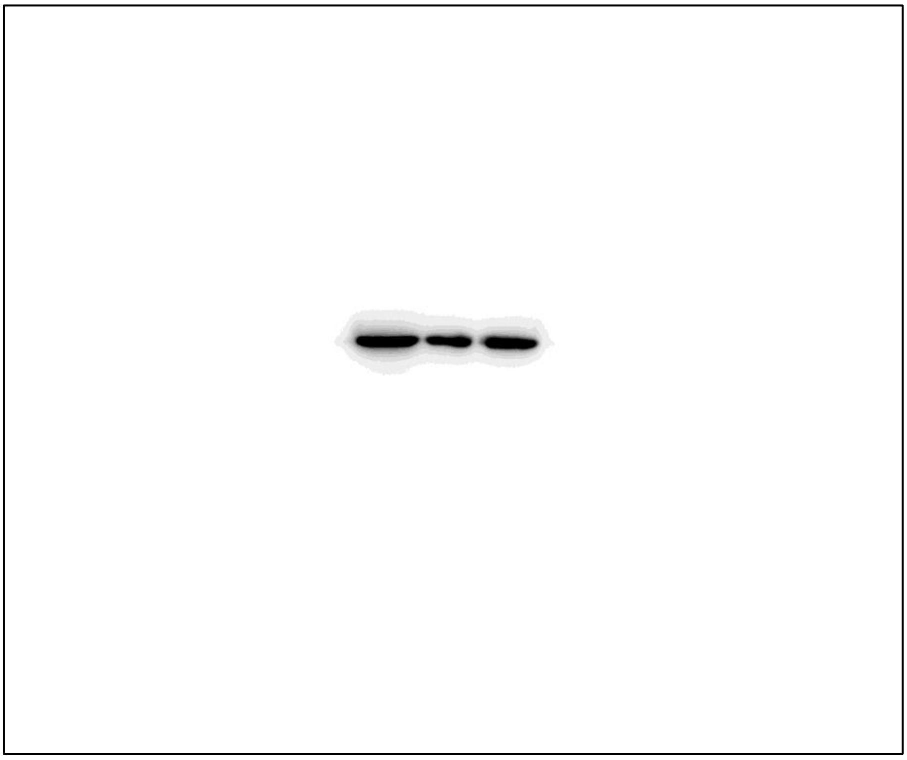

Fig 7I Claudin-3

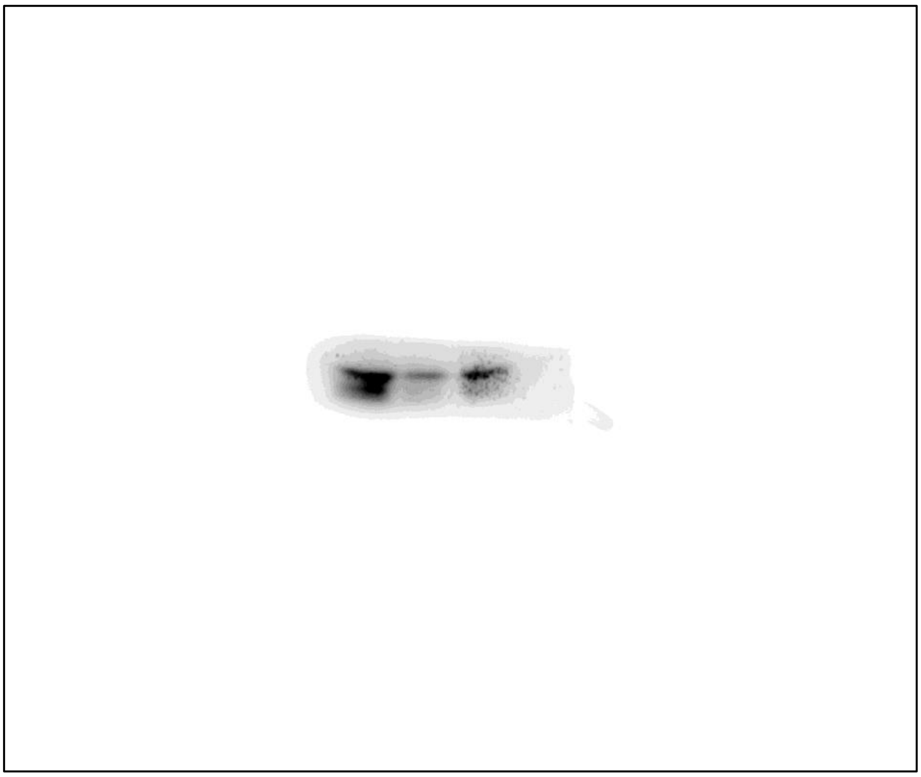

Fig 7I actin

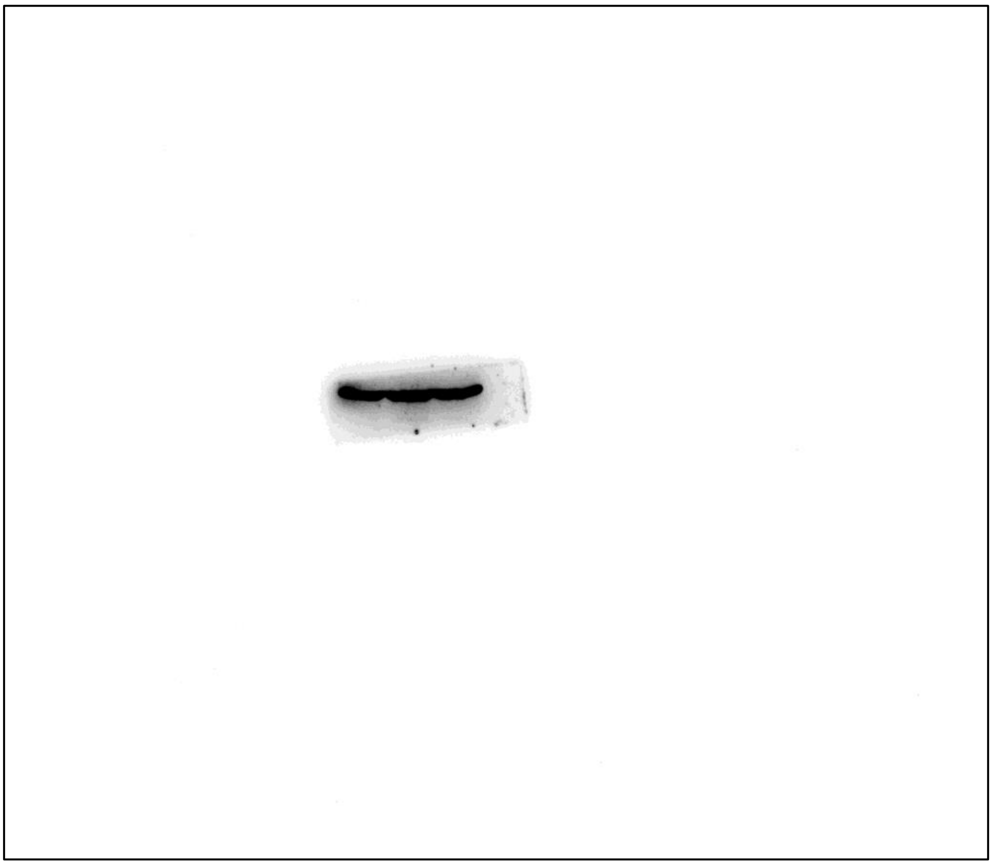

Fig 7M p-p65

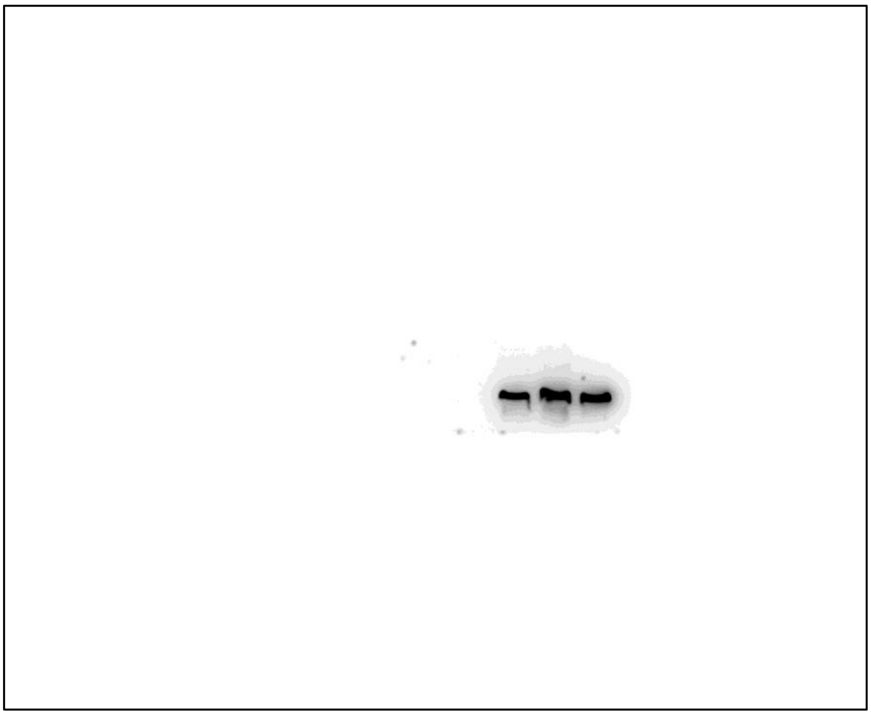

Fig 7M p65

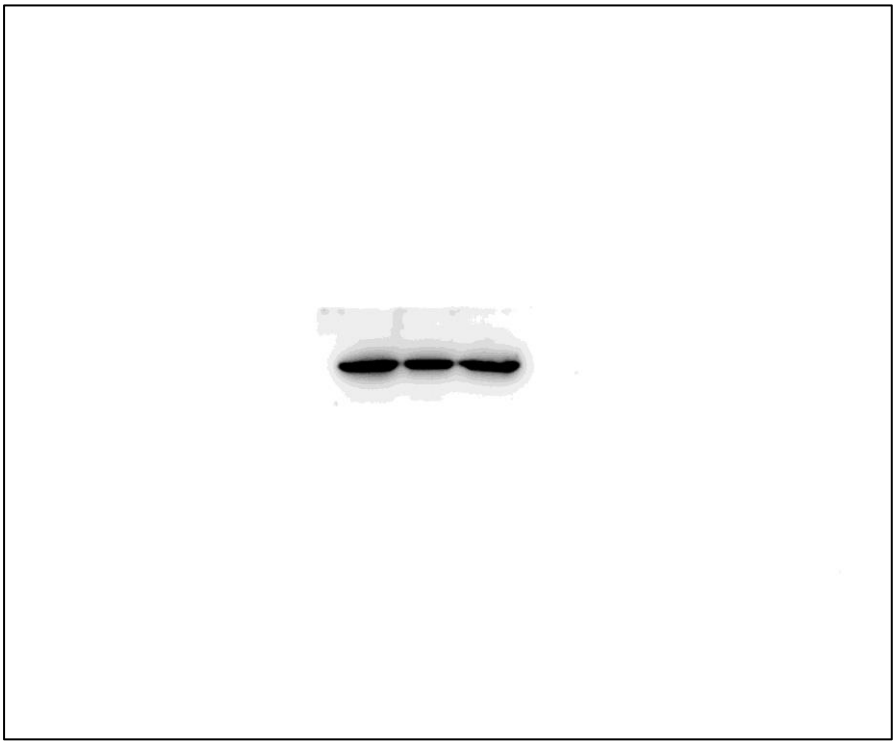

Fig 7M NLRP3

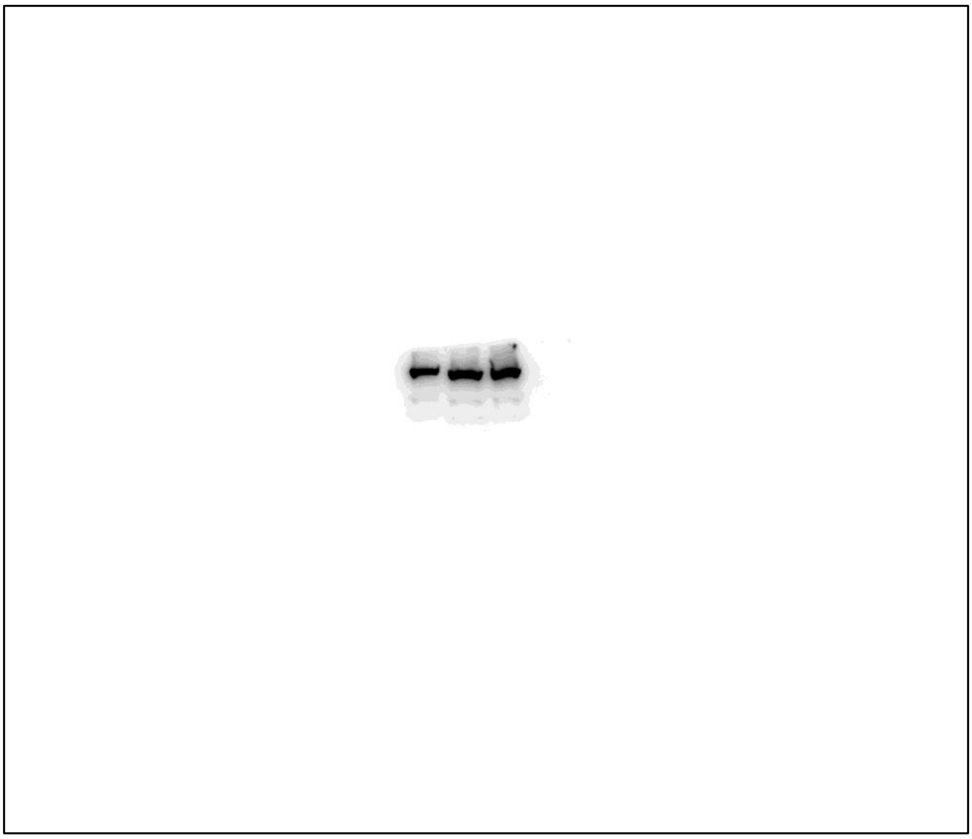

Fig 7M ASC

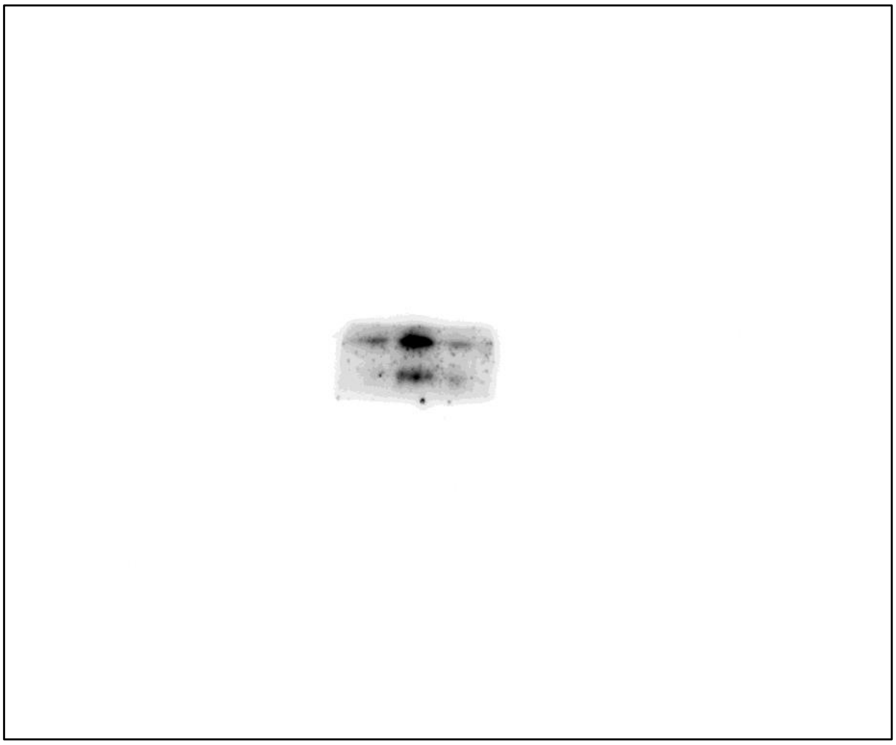

Fig 7M IL-1β

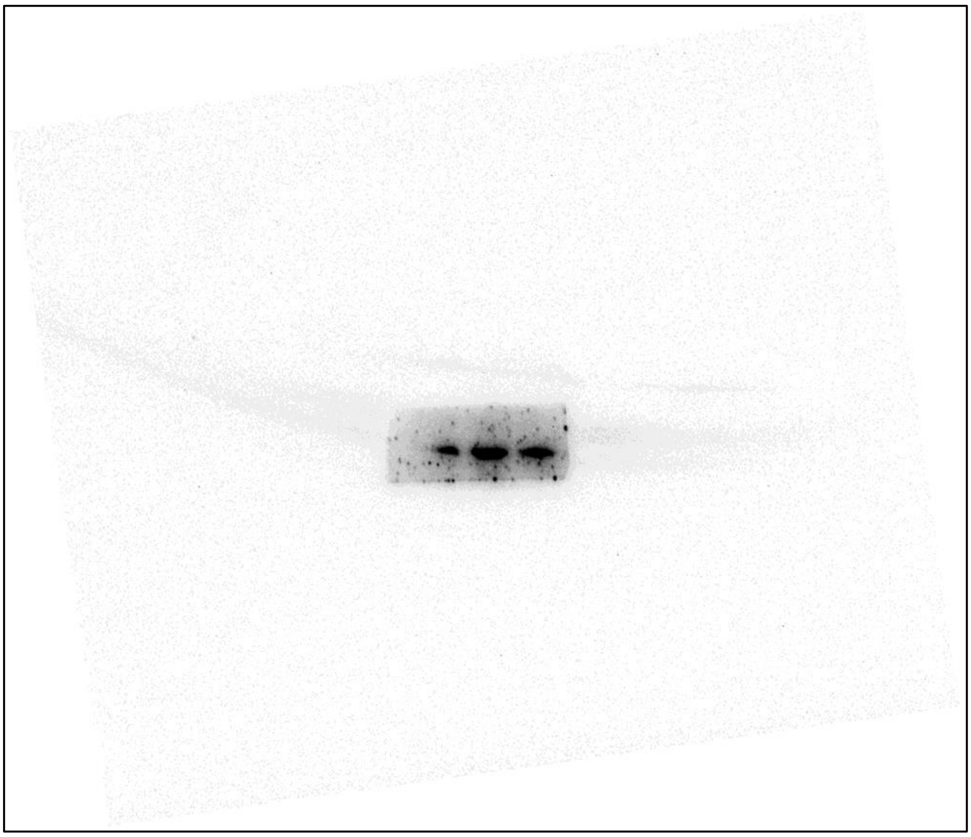

Fig 7M actin

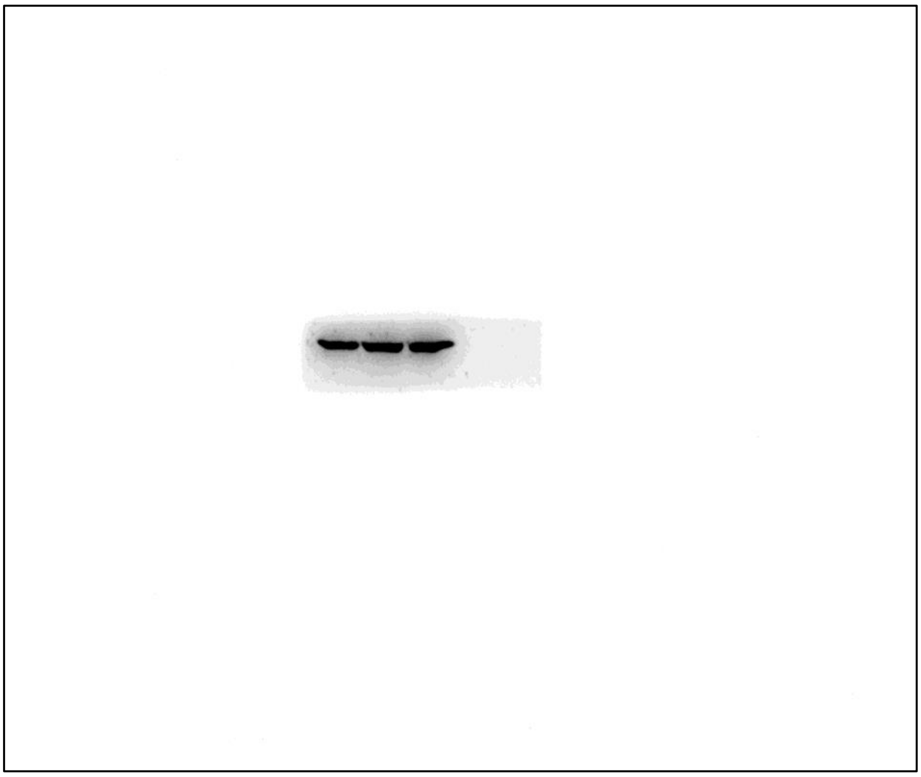

Supplement: Supplementary file 1 — Supplementary information [file 41522_2023_374_MOESM1_ESM.pdf]
