# Supplementary material for: Gut microbiota-mediated secondary bile acid alleviates Staphylococcus aureus-induced mastitis through the TGR5-cAMP-PKA-NF-κB/NLRP3 pathways in mice
Source: NPJ Biofilms Microbiomes. 2023 Feb 8;9:8. doi: 10.1038/s41522-023-00374-8 (PMC9908919; doi:10.1038/s41522-023-00374-8)
Supplement: Supplementary file 2 — Reporting Summary [file 41522_2023_374_MOESM2_ESM.pdf]

nature portfolio

Corresponding author(s): Yunhe Fu and Xiaoyu Hu

Last updated by author(s): Dec 22, 2022

Reporting Summary

Nature Portfolio wishes to improve the reproducibility of the work that we publish. This form provides structure for consistency and transparency in reporting. For further information on Nature Portfolio policies, see our Editorial Policies and the Editorial Policy Checklist.

Statistics

For all statistical analyses, confirm that the following items are present in the figure legend, table legend, main text, or Methods section.

n/a	Confirmed	
☐	☒	The exact sample size (*n*) for each experimental group/condition, given as a discrete number and unit of measurement
☐	☒	A statement on whether measurements were taken from distinct samples or whether the same sample was measured repeatedly
☐	☒	The statistical test(s) used AND whether they are one- or two-sided<br>*Only common tests should be described solely by name; describe more complex techniques in the Methods section.*
☒	☐	A description of all covariates tested
☒	☐	A description of any assumptions or corrections, such as tests of normality and adjustment for multiple comparisons
☐	☒	A full description of the statistical parameters including central tendency (e.g. means) or other basic estimates (e.g. regression coefficient) AND variation (e.g. standard deviation) or associated estimates of uncertainty (e.g. confidence intervals)
☒	☐	For null hypothesis testing, the test statistic (e.g. *F*, *t*, *r*) with confidence intervals, effect sizes, degrees of freedom and *P* value noted<br>*Give P values as exact values whenever suitable.*
☒	☐	For Bayesian analysis, information on the choice of priors and Markov chain Monte Carlo settings
☒	☐	For hierarchical and complex designs, identification of the appropriate level for tests and full reporting of outcomes
☒	☐	Estimates of effect sizes (e.g. Cohen's *d*, Pearson's *r*), indicating how they were calculated

Our web collection on statistics for biologists contains articles on many of the points above.

Software and code

Policy information about availability of computer code

Data collection	no software was used
Data analysis	GraphPad Prism 8

For manuscripts utilizing custom algorithms or software that are central to the research but not yet described in published literature, software must be made available to editors and reviewers. We strongly encourage code deposition in a community repository (e.g. GitHub). See the Nature Portfolio guidelines for submitting code & software for further information.

Data

Policy information about availability of data

All manuscripts must include a data availability statement. This statement should provide the following information, where applicable:

- Accession codes, unique identifiers, or web links for publicly available datasets
- A description of any restrictions on data availability
- For clinical datasets or third party data, please ensure that the statement adheres to our policy

The 16S rRNA gene sequencing data are available in NCBI Sequence Read Archive (SRA) repository under accession number PRJNA892063

Human research participants

Policy information about studies involving human research participants and Sex and Gender in Research.

Reporting on sex and gender	n/a
Population characteristics	n/a
Recruitment	n/a
Ethics oversight	n/a

Note that full information on the approval of the study protocol must also be provided in the manuscript.

Field-specific reporting

Please select the one below that is the best fit for your research. If you are not sure, read the appropriate sections before making your selection.

☒ Life sciences ☐ Behavioural & social sciences ☐ Ecological, evolutionary & environmental sciences

For a reference copy of the document with all sections, see nature.com/documents/nr-reporting-summary-flat.pdf

Life sciences study design

All studies must disclose on these points even when the disclosure is negative.

Sample size	The number of animals and group sizes were calculated via a priori power analysis using G*Power software (version 3.1, Universität Kiel, Kiel, Germany) and indicated in the figure legends. The effect size f, as defined by Cohen (1988), was determined using the population mean, α (error probability) was 0.05 and power (1-β error probability) was set to 0.8.
Data exclusions	no data were excluded
Replication	The results are presented as random one of three replicates
Randomization	The animals were randomly divided into groups via group randomization to ensure that each experiment had an equal sample size at all time points.
Blinding	In all experiments, investigators were group-blinded for all parameters including all data acquisition, sample processing, and data analysis.

Reporting for specific materials, systems and methods

We require information from authors about some types of materials, experimental systems and methods used in many studies. Here, indicate whether each material, system or method listed is relevant to your study. If you are not sure if a list item applies to your research, read the appropriate section before selecting a response.

Materials & experimental systems

n/a	Involved in the study	
☐	☒	Antibodies
☐	☒	Eukaryotic cell lines
☒	☐	Palaeontology and archaeology
☐	☒	Animals and other organisms
☒	☐	Clinical data
☒	☐	Dual use research of concern

Methods

n/a	Involved in the study	
☒	☐	ChIP-seq
☒	☐	Flow cytometry
☒	☐	MRI-based neuroimaging

Antibodies

Antibodies used	phosphorylation-p65 (p-p65, #AF2006; RRID: AB_2834435), p-65 (#AF5006; RRID: AB_2834847), p-IκB (#AF2002; RRID: AB_2834433), IκB (#AF5002; RRID: AB_2834792), Occludin (#DF7504; RRID: AB_2841004), ZO-1 (#AF5145; RRID: AB_2837631), Claudin-3 (#AF0129; RRID: AB_2833313) and β-actin (#AF7018; RRID: AB_2839420) were obtained from Affinity Biosciences (OH, USA). NLRP3 (#15101), ASC (#67824) and IL-1β (#12242) were bought from Cell Signaling Technology (CST, Boston, USA). Goat anti-rabbit or Rabbit anti-mouse secondary antibodies were bought from ImmunoWay Biotechnology Company.

Validation	All antibodies have been validation.

Eukaryotic cell lines

Policy information about cell lines and Sex and Gender in Research

Cell line source(s)	HC11 cells were obtained from the American Type Culture Collection (ATCC, CRL-3062)
Authentication	none of the cell lines were authenticated
Mycoplasma contamination	All cell lines tested negative for mycoplasma contamination
Commonly misidentified lines (See ICLAC register)	n/a

Animals and other research organisms

Policy information about studies involving animals; ARRIVE guidelines recommended for reporting animal research, and Sex and Gender in Research

Laboratory animals	BABL/c mice (6-8weeks, 22-24 g)
Wild animals	the study did not involve wild animals
Reporting on sex	Findings apply to only female.
Field-collected samples	The mice were raised with enough food and water in SPF grade feeding conditions with 12 h light and 12 h dark daily for a week. After adapting to the feeding environment, these mice were mixed at a ratio of three females to one male in separated cages with the same feeding conditions. After confirming pregnancy by the observation of vaginal spermatozoa, the male mice were removed.
Ethics oversight	All animal experiments were subject to approval by the Institutional Animal Care and Use Committee (IACUC) of Jilin University (China). The full proposal was considered by the IACUC ethics committee, which approved the animal care and use permit license. All experiments complied with the manual of the care and use of laboratory animals published by the US National Institutes of Health.

Note that full information on the approval of the study protocol must also be provided in the manuscript.
